# Dose finding study for on-demand HIV pre-exposure prophylaxis for insertive sex in sub-Saharan Africa: results from the CHAPS open label randomised controlled trial

**DOI:** 10.1016/j.ebiom.2023.104648

**Published:** 2023-06-14

**Authors:** Carolina Herrera, Jennifer Serwanga, Laura Else, Lebina Limakatso, Daniel Opoka, Andrew S. Ssemata, Azure-Dee Pillay, Patricia Namubiru, Thabiso B. Seiphetlo, Geoffrey Odoch, Susan Mugaba, Portia Seatlholo, Amara Alieu, Sujan Dilly Penchala, Richard Muhumuza, Berenice Alinde, Stefan Petkov, Kyle O'Hagan, Christian Callebaut, Janet Seeley, Helen Weiss, Saye Khoo, Francesca Chiodi, Clive M. Gray, Pontiano Kaleebu, Emily L. Webb, Neil Martinson, Julie Fox, CHAPS, Nadia Ahmed, Berenice Alinde, Amara Alieu, Millicent Atujuna, Esther Awino, Linda-Gail Bekker, Christian Callebaut, Francesca Chiodi, Mike Chirenje, Janan Dietrich, Jeffrey Dorfman, Laura Else, Julie Fox, Clive Gray, Christian Holm Hansen, Carolina Herrera, Stefanie Hornschuh, Ayoub Kakande, Pontiano Kaleebu, Charles Kelly, Saye Khoo, Mamkiri Khunwane, Limaktso Lebina, Joseph Makhura, Nomvuyo Mangxilana, Neil Martinson, Susan Mugaba, Richard Muhumuza, Freddie Mukasa Kibengo, Gertrude Mutonyi, Lucia Mungate, Winnie Nabukeera, Rehema Nagawa, Phiona Nalubega, Patricia Namubiru, Stephen Nash, Denis Ndekezi, Teacler Nematadzira, Lumka Nobula, Kyle O'Hagan, Geoffrey Odoch, Daniel Opoka, Sujan Dilly Penchala, Stefan Petkov, Azure-Dee Pillay, Jim Rooney, Elzette Rousseau, Eugene Ruzagira, Alison Sango, Ntombexolo Seatlholo, Janet Seeley, Thabiso Seiphetlo, Jennifer Serwanga, Robin Shattock, Andrew S. Ssemata, Lynda Stranix-Chibanda, Gugulethu Tshabalala, Emily Webb, Helen Weiss

**Affiliations:** a Department of Infectious Disease, Faculty of Medicine, Imperial College London, Norfolk Place, W2 1PG, London, UK; b Medical Research Council/Uganda Virus Research Institute, and London School of Hygiene and Tropical Medicine, Uganda Research Unit, 51-59 Nakiwogo Road, Entebbe, Uganda; c Department of Molecular and Clinical Pharmacology, William Henry Duncan Building, University of Liverpool, 6 West Derby Street, Liverpool, L7 8TX, UK; d Perinatal HIV Research Unit, University of the Witwatersrand, Johannesburg, South Africa; e Africa Health Research Unit, Durban, South Africa; f Department of Global Health and Development, London School of Hygiene & Tropical Medicine, 15-17 Tavistock Place, London, WC1H 9SH, UK; g Division of Immunology, University of Cape Town, South Africa based at Respiratory and Meningeal Pathogens Research Unit (RMPRU). Chris Hani Baragwanath Hospital, 30 Chris Hani Road, Diepkloof, Soweto, 1862, South Africa; h Division of Immunology, Institute of Infectious Disease and Molecular Medicine, Department of Pathology, University of Cape Town, Cape Town, South Africa; i Department of Microbiology, Tumor and Cell Biology, Karolinska Institutet, Solnavägen 9, 171 65 Solna, Sweden; j Gilead Sciences, Foster City, CA 94404, USA; k Department of Global Health and Development, London School of Hygiene & Tropical Medicine, 15-17 Tavistock Place, London, WC1H 9SH, UK; l Medical Research Council/Uganda Virus Research Institute, and London School of Hygiene and Tropical Medicine, Uganda Research Unit, 51-59 Nakiwogo Road, Entebbe, Uganda; m MRC International Statistics and Epidemiology Group, London School of Hygiene & Tropical Medicine, Keppel Street, London, WC1E 7HT, UK; n Division of Molecular Biology and Human Genetics, Department of Biomedical Sciences, Biomedical Research Institute, Stellenbosch University (Tygerberg Campus), Francie van Zijl Drive, Tygerberg, Cape Town, South Africa; o Johns Hopkins University Center for TB Research, Baltimore, MD, USA; p Infection and Immunity, Borough Wing, Guys and St Thomas' NHS Foundation Trust and King's College London, St. Thomas Street, SE1 9RS, London, UK

**Keywords:** HIV-1, Foreskin, Oral PrEP, Adolescents, PK/PD, Tissue explants

## Abstract

**Background:**

The efficacy of on-demand HIV pre-exposure prophylaxis (PrEP) for men in sub-Saharan Africa has not been evaluated, and the on-demand PrEP dosing requirement for insertive sex remains unknown.

**Methods:**

HIV-negative males 13–24 years, requesting voluntary medical male circumcision (VMMC), were enrolled into an open-label randomised controlled trial (NCT03986970), and randomised 1:1:1:1:1:1:1:1:1 to control arm or one of eight arms receiving emtricitabine-tenofovir disoproxil fumarate (F/TDF) or emtricitabine-tenofovir alafenamide (F/TAF) over one or two days, and circumcised 5 or 21 h thereafter. The primary outcome was foreskin p24 concentrations following *ex vivo* HIV-1_BaL_ challenge. Secondary outcomes included peripheral blood mononuclear cell (PBMC) p24 concentration, and drug concentrations in foreskin tissue, PBMCs, plasma and foreskin CD4+/CD4-cells. In the control arm, post-exposure prophylaxis (PEP) activity of non-formulated tenofovir-emtricitabine (TFV-FTC) or TAF-FTC was assessed with *ex vivo* dosing 1, 24, 48 or 72 h post-HIV-1 challenge.

**Findings:**

144 participants were analysed. PrEP with F/TDF or F/TAF prevented *ex vivo* infection of foreskins and PBMCs both 5 and 21 h after PrEP dosing. There was no difference between F/TDF and F/TAF (p24_day15_ geometric mean ratio 1.06, 95% confidence interval: 0.65–1.74). Additional *ex vivo* dosing did not further increase inhibition. In the control arm, PEP *ex vivo* dosing was effective up to 48 post-exposure diminishing thereafter, with TAF-FTC showing prolonged protection compared to TFV-FTC. Participants receiving F/TAF had higher TFV-DP concentrations in foreskin tissue and PBMCs compared with F/TDF, irrespective of dose and sampling interval; but F/TAF did not confer preferential TFV-DP distribution into foreskin HIV target cells. FTC-TP concentrations with both drug regimens were equivalent and ∼1 log higher than TFV-DP in foreskin.

**Interpretation:**

A double dose of either F/TDF or F/TAF given once either 5 or 21 h before *ex vivo* HIV-challenge provided protection across foreskin tissue. Further clinical evaluation of pre-coital PrEP for insertive sex is warranted.

**Funding:**

EDCTP2, 10.13039/100005564Gilead Sciences, 10.13039/501100004359Vetenskapsrådet.

**Research in context**

**Evidence before this study**

We searched PubMed and Clinicaltrials.gov for combinations of the terms “tenofovir disoproxil fumarate and emtricitabine”, “tenofovir alafenamide and emtricitabine”, “foreskin”, “male genital tract”, “peripheral blood mononuclear cells”, “HIV pre-exposure prophylaxis”, and “HIV post-exposure prophylaxis”, with no restrictions on language or publication date. Seventy-one clinical trials on F/TDF or F/TAF have been completed or are ongoing, but none has tested efficacy against HIV-1 infection in foreskin tissue of on-demand PrEP in men in sub-Saharan Africa (SSA). Drug concentrations in human male genital tract have only been evaluated in secretions which do not precisely reflect tissue drug concentrations. No clinical trial comparing the efficacy and time interval from HIV-1 exposure to PEP initiation for F/TDF and F/TAF was identified.

**Added value of this study**

This clinical trial uses *ex vivo* challenge methods evaluating dosing requirements for on-demand PrEP in insertive sex using F/TDF and F/TAF. We show that a double dose of either F/TDF or F/TAF PrEP given 5 or 21 h before exposure, is sufficient to prevent HIV acquisition across both foreskin tissue and PBMCs. There was no difference in protection from HIV-1 between F/TDF and F/TAF, however, F/TAF produced higher intracellular drug concentrations in both PBMCs and target cells within foreskin tissue.

Both drugs showed significant PEP activity in foreskin explants however, protection was limited after 48 h, with TAF-FTC providing more durable protection compared to TFV-FTC.

**Implications of all the available evidence**

Understanding dosing requirements for insertive sex is necessary to provide guidance for optimal use. The high concentration of protection found in this study of foreskins from men in Uganda and South Africa provides for the data for insertive sex in men from SSA. Our data suggest that on-demand PrEP could be simplified to a double dose of F/TDF or F/TAF given 5–21 h prior to intercourse. The limited efficacy of TFV-FTC or TAF-FTC as PEP when initiated 48 h after HIV exposure supports evidence from rectal tissue that PEP initiation should be within 48 h of potential exposure to HIV.

## Introduction

Despite cheap and effective HIV testing and prevention, approximately 320,000 men were newly infected in sub-Saharan Africa (SSA) in 2021.^1^ HIV prevention efforts in SSA are primarily female-focused, leaving a gap for men gap in HIV services.^2^ Men fare worse in HIV testing uptake, ART initiation,^3^ engagement and retention in HIV prevention/treatment programmes.^4^, ^5^, ^6^

Oral HIV PrEP has achieved tremendous impact in reducing HIV incidence in Europe and Americas but less in Africa, where PrEP availability is variable.^7^ Daily and on-demand PrEP are highly effective and recommended by WHO for use in men.^8^ Clinical efficacy of on-demand PrEP has only been evaluated in men who have sex with men (MSM),^9^ with no data in other populations including young men in SSA. Hence, on-demand PrEP is not included in SSA guidelines, despite men from the region preferring on-demand PrEP, specifically pre-coital PrEP regimes over daily tablets.^10^^,^^11^ Regimen simplification for on-demand PrEP is desirable to increase adherence and could be appropriate for insertive sex, which is associated with a 10-fold lower risk of HIV acquisition compared to receptive sex.^12^ On-demand PrEP has advantages over daily PrEP: lower number of tablets required; lower risk of drug resistance selection; and fewer adverse events.

F/TAF is licenced for daily PrEP but has not been evaluated for on-demand PrEP. TAF is known to achieve up to 4.1-fold higher concentrations of the intracellular active phosphorylated moiety (tenofovir diphosphate; TFV-DP) in PBMCs,^13^ and has a more favourable renal and bone toxicity profile than TDF.^14^ Understanding how rapidly HIV protection occurs following PrEP dosing is vital for both daily and on-demand PrEP. Whilst the IPERGAY study provides insight for MSM in Europe initiating F/TDF,^9^ in other settings and for those initiating F/TAF, data for insertive sex are not known. PEP remains a useful additional strategy to prevent HIV acquisition.^12^ However, there is no clinical data guiding how soon after sex PEP must be started for insertive sex and no comparison between TAF and TDF.

*Ex vivo* challenge models provide a platform to define markers of biological efficacy and have been used to prioritize HIV-1 prevention candidates for Phase 3 trials.^15^ Voluntary medical male circumcision (VMMC) has been scaled-up in many high HIV-prevalence settings in SSA as part of prevention efforts. Many young men choose to become circumcised in Africa each year providing a potential research opportunity to utilise foreskins which otherwise would be discarded. We conducted a clinical trial comparing efficacy of F/TDF *versus* F/TAF dosing prior to VMMC on HIV-infection of foreskin using an *ex vivo* challenge model. We used foreskin tissue obtained to ascertain dosing schedules, duration of protection against *ex vivo* challenge, and additional protection afforded by treatment after *ex vivo* exposure to HIV. This trial simulated the relative protection that could be attained for insertive sex by pre- and post-exposure regimens.

## Methods

### Study design and participants

HIV negative males aged 13–24 years were recruited from VMMC clinics at Chris Hani Baragwanath Academic Hospital, Soweto, South Africa or Entebbe General Hospital, Entebbe, Uganda. Eligibility criteria included being clinically eligible for VMMC, weighing >35 kg, and being able to give written informed consent. Full eligibility criteria are described in the protocol (appendix: study protocol). This was a randomised controlled trial with nine trial arms: one control arm and eight treated arms that received PrEP prior to VMMC (Supplementary Fig. S1).^16^ The eight treated arms varied combinations of three binary conditions:

i. Drug: F/TDF or F/TAF (Gilead, Foster City, CA, USA)
ii. Dose: double dose on day one, or double dose on day one and single dose on day 2 (2 + 1).
iii. Time from last PrEP dose to VMMC: 5 h or 21 h

### Randomization and masking

Participants were randomised in a 1:1:1:1:1:1:1:1:1 ratio to control arm or one of the eight treated arms. Random allocation sequence was generated by an independent statistician using Stata, stratified by country and using block size 9. Participants who were not circumcised following randomisation were excluded according to protocol, and an additional set of randomisation codes generated using the same approach, to ensure a target sample size of 16 evaluable participants per trial arm was attained. Sequentially numbered opaque envelopes labelled with unique randomisation identifier and containing the allocated intervention arm were prepared by two administrators otherwise uninvolved in the study. At time of randomisation, clinical staff opened the sequential envelope and scheduled the participant to receive PrEP (if applicable) and VMMC, as per randomisation arm. Participants and care providers were not blinded to trial arm. Laboratory outcome assessors were blinded until all measurements were completed.

### Ethical approvals

Written informed consent was obtained from all participants aged ≥18 years and emancipated minors (in Uganda); for those <18 years and not emancipated minors, assent with parental consent was obtained. The trial was conducted in accordance with the principles of the Declaration of Helsinki and Good Clinical Practice and approved in the South African Health Products Regulatory Authority (20181004). Ethical approval was granted from University of Cape Town (290/2018), University of the Witwatersrand (180906 B, M1811148, 180108), Uganda Virus Research Institute research ethics committee (GC/127/18/12/680), Uganda National Council of Science and Technology (HS2534), Uganda National Drug Authority (618/NDA/DPS/09/2019), and London School of Hygiene and Tropical Medicine research ethics committee (17403). An independent Data Safety and Monitoring Board evaluated trial progress and safety data twice during recruitment; no interim analyses were done.

### Outcomes

The primary outcome was HIV-1 p24 concentration in participants' foreskin tissue up to day 15 following *ex vivo* HIV-challenge and evaluated as p24 concentration at day 15 (p24_d15_), area-under-the-curve of p24 concentrations between days 3–15 (p24_AUC_) following challenge, and slope of the curve fitted to p24 concentrations between days 3–15 (p24_slope_). Reduction of p24_d15_, p24_AUC_ and negative p24_slope_ were indicative of protection, i.e. lack of productive HIV-1 infection.

The secondary outcomes included p24 concentrations in PBMCs; drug concentration in foreskin tissue, foreskin CD4+/CD4-cells, blood (plasma and PBMCs); additional effect of *ex vivo* dosing with the same oral PrEP drug 20 h post-challenge; and *ex vivo* PEP efficacy in foreskin from participants in the control arm.

### Sampling

Study samples were collected during VMMC visit 5 h or 21 h ± 40 min after the last PrEP dosing. Blood and foreskin tissue, including inner and outer, were collected. VMMC was performed using the dorsal slit method according to local guidelines. Tissue was placed immediately in cold culture media DMEM. Samples were immediately transported to the laboratory on ice (median transit time 30 min). Processing of samples was performed immediately upon arrival to the laboratory.

### Isolation of peripheral blood mononuclear cells

PBMC were isolated by density-gradient centrifugation using Lymphoprep™ (Stem Cell Technologies, Vancouver, Canada) followed by erythrocyte lysis (ACK Lysing buffer, Gibco, Waltham, MA, USA). For pharmacodynamic (PD) analysis, cells were resuspended in RPMI 1640 supplemented with 10% fetal bovine serum (FBS), 2 mM l-glutamine and antibiotics (100 U of penicillin/mL, 100 μg of streptomycin/mL) (Sigma, St. Louis, MO, USA). Alternatively, for pharmacokinetic (PK) assessments, isolated PBMCs were lysed with ice cold chelating solution (methanol:20 mM EDTA-20 mM EGTA 70:30 v/v).

### Ex vivo challenge of foreskin tissue and PBMCs

Foreskin was cut into 2 mm^2^ explants^17^ and cultured in DMEM supplemented with 10% FBS, 2 mM l-glutamine, 2.5 μg of amphotericin B/mL, 100 U of penicillin/mL, 100 μg of streptomycin/mL, and 80 μg of gentamicin/mL (Sigma, St. Louis, MO, USA). Explants were immediately mock treated or challenged with HIV-1_BaL_ at a high titre (HVT) (10^4^ TCID_50_/mL [median tissue culture infective dose/mL]) generally used for *ex vivo* challenge or at a more biologically relevant low titre (LVT) (2 × 10^2^ TCID_50_/mL). After 20 h, explants from treated arms were further dosed *ex vivo* for 2 h, or not for controls cases, with non-formulated TFV-FTC or TAF-FTC (Gilead, Foster City, CA, USA) matching the drug used for oral dosing, to mimic PrEP regimes including dosing after sexual intercourse, and with the concentrations previously determined in foreskin explants.^18^ *Ex vivo* dosing of explants was performed in a non-polarized manner as described previously^18^ at concentrations previously defined in our laboratory.^17^^,^^18^ Following drug exposure, tissue explants were washed with PBS, and cultured in complete media in the absence of drug.

Explants from the control arm were used as baseline of infection. Additional explants from the control arm were dosed *ex vivo* with TFV-FTC or TAF-FTC for 2 h at different times post-viral exposure: 1, 24, 48 or 72 h to assess the PEP potency of these drugs. Isolated PBMCs were challenged or not with HIV-1_BaL_ at a HVT (10^3^ TCID_50_/mL) or a LVT (2 × 10^2^ TCID_50_/mL), and after 20 h, dosed or not *ex vivo* with the same drug used for oral dosing. PBMCs from the control arm were not further dosed *ex vivo*. All PBMCs were then activated with PHA 5 μg/mL and IL-2 20U/mL for 48 h. Foreskin explants and PBMCs were cultured for 15 days with two-thirds of culture supernatant harvested at days 3, 7, 11 and 15, and cultures refed with fresh medium. The extent of virus replication was determined by measuring p24 concentration in supernatants at each harvest time point following manufacturer's instructions (Innotest HIV antigen mAb ELISA, catalogue number: 80563, Fujirebio Europe, Belgium). Non-cumulative p24 values were extrapolated using a sigmoidal dose–response (Prism, GraphPad) and reported as p24_d15_ and p24_AUC_. The p24_slope_ was calculated during the 15 days of culture. The lower limit of quantification (LLQ) for the assay was 0.02998. p24 concentrations that were below the LLQ of each assay were expressed as half-LLQ values.

### Isolation of CD4+ and CD4-cells from foreskin

Foreskin tissue was cut into 4 mm^2^ explants, dissociated in a GentleMACS tissue dissociator with human whole skin dissociation kit following manufacturer's instructions (catalogue number: 130-101-540, Miltenyi Biotec, Bergisch Gladbach, Germany). CD4+ and CD4-cells were isolated with Dynabeads CD4 Positive Isolation Kit (catalogue number: 11331D, Invitrogen, Waltham, MA, USA), counted prior to lysis with ice cold chelating solution, and stored at −80 °C.

### Bioanalytical methods

Concentrations (denoted as [drug or metabolite]) of TFV, FTC, and the pro-drug TAF, were measured in plasma and foreskin tissue. Concentrations of the active phosphorylated intracellular metabolites – tenofovir-diphosphate (TFV-DP) and emtricitabine-triphosphate (FTC-TP) were determined in PBMCs, foreskin tissue, and isolated foreskin CD4+ and CD4-cells. Analyte quantification was performed using a SCIEX 4500 or 5500 triple quadrupole mass spectrometer (AB Sciex UK Limited; Warrington, UK). Data acquisition and processing were performed using FDA CFR Part 11 compliant SCIEX Analyst and Multiquant software platforms. LC-MS assays were validated in accordance with FDA Bioanalytical Method Validation Guidelines.^19^ Relevant bioanalytical method instrumentation and assay validation parameters are summarised in Supplementary Tables S1 and S2 Drug and metabolite concentrations in foreskin were quantified using a ng/sample or pmol/sample calibration curve and values normalised to ng/g or pmol/g of tissue. Intracellular concentrations of phosphorylated metabolites in PBMCs and CD4± cells were expressed as mol/10^6^ cells. Concentrations below the assay LLQ were expressed as half-LLQ values. Concentrations below the assay lower limit of detection (with no visible chromatographic signal) were excluded.

### Statistical analysis

The planned sample size of 144 participants was based on feasibility of conducting this number of experiments based on previous *ex* vivo challenge models. Analysis was done on a per protocol basis. Participant characteristics were summarised by trial arm, using frequency and proportion for categorical variables and mean and standard deviation for continuous variables. All PD and PK outcomes were positively skewed and therefore log-transformed for analysis; log-transformed variables were approximately normally distributed. Outcomes were summarised using geometric means and 95% confidence interval (CI). The relative effect of trial interventions on primary and secondary outcomes was assessed through the following *a-priori* defined comparisons: (i) any PrEP *versus* control arm, (ii) FTC-TAF *versus* FTC-TDF, (iii) 2 + 1 tablets *versus* 2 tablets, (iv) 21 h between PrEP and VMMC *versus* 5 h. Further comparisons also assessed the effect of dosage separately for each drug, and the effect of interval, separately for each drug and dosage. Linear regression was used to determine the mean difference and 95% CI for each comparison, with parameters back-transformed to geometric mean ratios (GMR), and p-values determined by likelihood ratio tests. We assessed assumptions underlying linear regression; homoscedasticity was assessed through plotting fitted values against residuals and normality of residuals was assessed through *QQ plots.* PBMC metabolite concentrations from this study were related to previously defined protective thresholds of greater than 16 fmol/10^6^ cells (TFV-DP) and 3.7 pmol/10^6^ cells (FTC-TP) which were associated with a 90% reduction in risk of HIV-1 acquisition.^20^ Additional effect of *ex vivo* dosing on p24 concentrations in tissue and PBMCs was assessed using paired t-tests, comparing p24 in tissue/PBMCs that received *ex vivo* dosing with p24 in tissue/PBMC that did not receive *ex vivo* dosing, with the comparison made within each participant. *Ex vivo* efficacy of PEP was compared between trial arms using independent t-tests. As a post-hoc analysis, we calculated the percentage reduction in p24 at day 15 in both tissue and PBMCs among participants receiving PrEP compared to participants in the control arm.

### Role of the funding source

Funders had no role in the data collection, data analysis or data interpretation. In addition, EDCTP2 and Vetenskapsrådet had no role in study design or writing of the report. All authors had full access to all study data and had final responsibility for the decision to submit for publication.

## Results

**Fig. 1:**
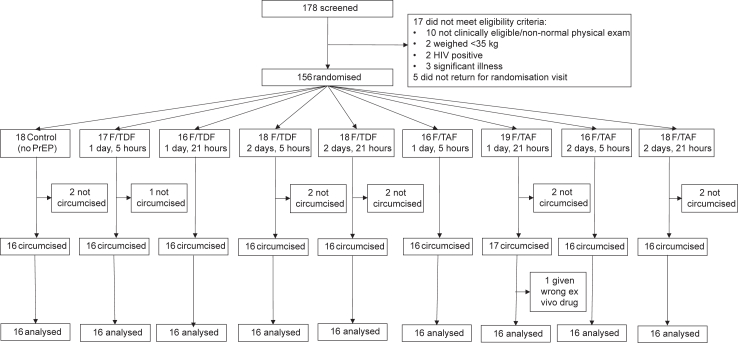
**Participant recruitment and follow-up**.

Between 19th October 2019 and 5th March 2021, 178 young men were screened, 17 did not meet eligibility criteria and five did not return for the randomisation visit, leaving 156 randomised participants. Of these, 11 were not circumcised and foreskin from one circumcised participant was given the wrong *ex vivo* drug in error; these all were judged non-evaluable and excluded from analyses. A total of 144 participants, 16 per trial arm, were analysed (Fig. 1). Characteristics of included participants were similar between trial arms (Table 1). Twenty-one adverse events were reported among 19 participants (Supplementary Table S3) with no serious adverse events.

**Table 1:** Baseline characteristics of CHAPS trial participants.

Characteristic	Control (n = 16)	F/TDF, 2 tabs (5 h) (n = 16)	F/TDF, 2 tabs (21 h) (n = 16)	F/TDF, 2 + 1 tabs (5 h) (n = 16)	F/TDF, 2 + 1 tabs (21 h) (n = 16)	F/TAF, 2 tabs (5 h) (n = 16)	F/TAF, 2 tabs (21 h) (n = 16)	F/TAF, 2 + 1 tabs (5 h) (n = 16)	F/TAF, 2 + 1 tabs (21 h) (n = 16)
Country									
South Africa	8	8	8	8	8	8	8	8	8
Uganda	8 (50%)	8 (50%)	8 (50%)	8 (50%)	8 (50%)	8 (50%)	8 (50%)	8 (50%)	8 (50%)
Age, mean (SD)	17.9 (3.7)	17.9 (3.4)	19.2 (3.1)	18.1 (3.5)	19.2 (2.8)	19.2 (2.4)	19.7 (2.5)	18.8 (3.2)	18.6 (4.0)
Weight (kg), mean (SD)	53.4 (10.1)	54.6 (10.9)	54.5 (6.2)	54.4 (10.5)	59.0 (8.4)	58.0 (8.9)	61.6 (8.1)	62.9 (12.0)	56.7 (10.3)
Height (cm), mean (SD)	165.3 (11.8)	162.9 (12.0)	164.6 (6.6)	165.1 (9.2)	163.4 (9.8)	166.6 (8.7)	169.3 (6.6)	172.2 (11.9)	164.4 (8.2)
Currently studying									
No	10	11	7	9	9	6	7	7	11
Yes	6 (38%)	5 (31%)	9 (56%)	7 (44%)	7 (44%)	10 (63%)	9 (56%)	9 (56%)	5 (31%)
Current relationship status^a^									
Single	9 (56%)	8 (53%)	8 (50%)	9 (57%)	8 (50%)	10 (63%)	6 (38%)	11 (69%)	9 (56%)
Boyfriend/girlfriend	7 (44%)	6 (40%)	8 (50%)	7 (44%)	7 (44%)	6 (38%)	10 (63%)	5 (31%)	7 (44%)
Other	0 (0%)	1 (7%)	0 (0%)	0 (0%)	1 (6%)	0 (0%)	0 (0%)	0 (0%)	0 (0%)
Ever had sex^b^									
No	8	4	12	10	11	10	12	13	8
Yes	8 (50%)	11 (73%)	4 (25%)	6 (38%)	5 (31%)	6 (38%)	4 (25%)	3 (19%)	7 (47%)
Chlamydia test result									
Negative	16	15	16	16	15	16	14	14	16
Positive	0 (0%)	1 (6%)	0 (0%)	0 (0%)	1 (6%)	0 (0%)	2 (13%)	2 (13%)	0 (0%)
Heard of PrEP for HIV prevention									
No	13	12	12	12	13	16	12	11	9
Yes	3 (19%)	3 (20%)	4 (25%)	4 (25%)	3 (19%)	0 (0%)	4 (25%)	5 (31%)	7 (44%)

a 1 participant preferred not to say.

b 2 participants preferred not to say.

**Fig. 2:**
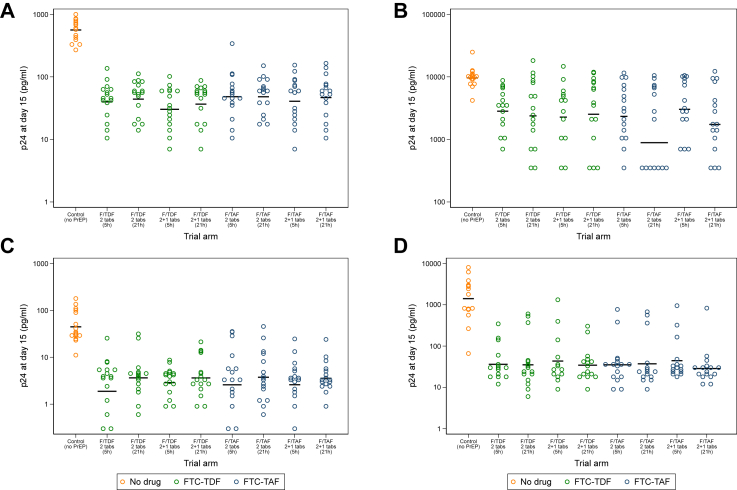
**Levels of p24 at day 15 in tissue and PBMCs following: (A) high titre challenge in tissue, (B) high titre challenge in PBMCs, (C) low titre challenge in tissue, (D) low titre challenge in PBMCs. Data are from n** = **144 with each condition tested in biological triplicates**.

Compared to the control arm, oral dosing with F/TDF or F/TAF, significantly decreased p24_d15_ in foreskin explant culture supernatants post-*ex vivo* challenge with HIV-1_BaL_ at both HVT and LVT [GMR (95% CI): 0.07 (0.05–0.11), p < 0.0001 and 0.07 (0.03–0.14), p < 0.0001 for HVT and LVT challenge, respectively; Fig. 2A,C; Supplementary Table S4] (p-values determined by likelihood ratio tests). There was no evidence of a difference in p24_d15_ by drug received [GMR (95% CI) comparing F/TAF with F/TDF 1.23 (0.93–1.61) and 1.06 (0.65–1.74) for HVT and LVT challenge respectively], dosing schedule [0.85 (0.65–1.11) and 1.10 (0.67–1.80) comparing 2 days to 1 day] or time to VMMC after the final dose [1.11 (0.85–1.46) and 1.48 (0.91–2.43), comparing 21 h–5 h]. Similarly, analysis of viral growth curves in foreskin explants showed significant reductions in both p24_AUC_ and p24_slope_ in all treated arms compared to the control arm: GMR 0.21 (0.17–0.28) and 0.21 (0.14, 0.31) for HVT and LVT challenge, respectively for p24_AUC_, and adjusted mean difference −20.2 (−29.0, −11.3) and −24.2 (−47.1, −1.3) with HVT and LVT challenge, respectively for p24_slope_) (Supplementary Table S4). Calculation of the percentage reduction in p24 among participants receiving PrEP compared to those in the control arm demonstrates that nearly all had >75% reduction in p24_d15_ (Supplementary Fig. S2A and C). Additional *ex vivo* dosing 20 h post-exposure to virus did not significantly increase the level of inhibition reached with oral dosing (Supplementary Fig. S2A and C; Supplementary Table S5). Furthermore, analysis of the viral replication curves suggested no evidence that additional *ex vivo* dosing further reduced p24 concentration in foreskin tissue observed with oral dosing (Supplementary Table S5).

In *ex vivo* challenged PBMCs, all oral PrEP arms significantly reduced p24 concentration compared to the control arm, with no evidence of differences between drug type, dosing or timing interval (Fig. 2B,D; Supplementary Fig. S2B and D; Supplementary Table S4). In contrast to foreskin, *ex vivo* dosing 20 h post-challenge further reduced p24 in PBMCs challenged with HVT and LVT (Fig. 2B,D; Supplementary Fig. S2B and D; Supplementary Table S5). This reduction in p24 production was seen regardless of regimen, dosing, or interval to VMMC (Supplementary Table S5).

**Fig. 3:**
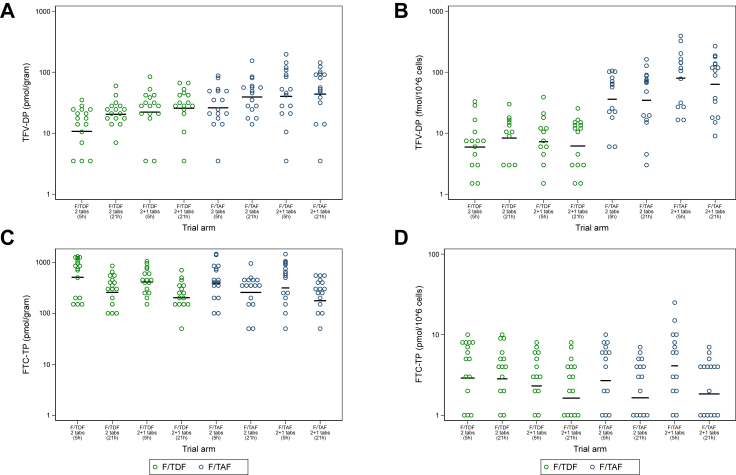
**Levels of tenofovir and emtricitabine active metabolites in tissue and PBMCs: (A) TFV-DP in tissue, (B) TFV-DP in PBMCs, (C) FTC-TP in tissue, (D) FTC-TP in PBMCs. Data are from n** = **144 with each condition tested in biological triplicates**.

Intracellular [TFV-DP] in PBMCs were 7.5-fold higher (95% CI: 5.0–11.2) with F/TAF [50.0 fmol/10^6^ cells (36.9–67.6)] *versus* F/TDF dosing [6.7 fmol/10^6^ cells (5.1–8.9)] (Fig. 3B, Supplementary Table S6). For participants receiving F/TAF, administration of a 2 + 1 dose resulted in 2-fold higher [TFV-DP] [70.8 fmol/10^6^ cells (47.0–106)], compared with 2 tablets [35.3 fmol/10^6^ cells (22.8–54.5)] (GMR: 2.01 (1.12, 3.60)). There was no evidence of TFV-DP accumulation in PBMCs over the 21 h sampling interval– [TFV-DP] were similar at 5 and 21 h post-dose (GMR 0.95 (0.63, 1.42)). Intracellular PBMC [FTC-TP] were in the pmol range (∼2.4 pmol/10^6^ cells) and were similar irrespective of regimen or dose (Fig. 3D, Supplementary Table S6), but lower for participants circumcised at 21 h than at 5 h post-dose (GMR 0.65 (0.43, 0.99)). [TFV-DP] and [FTC-TP] in PBMCs above 16 fmol/10^6^ cells (95% CI: 3–28) and 3.7 pmol/10^6^ cells (95% CI: 1.2–6.1) have been associated with a 90% reduction in the risk of HIV-1 acquisition.^20^ In our study, [TFV-DP] >16 fmol/10^6^ cells were obtained in 78% participants (72% 2 tablets; 84% 2 + 1) receiving on-demand F/TAF; whereas only 16% of participants receiving F/TDF achieved this threshold with a double dose, and 13% after a 2 + 1 dose. [FTC-TP] in PBMCs were in the range of previous studies^21^ and there was no evidence that they were influenced by the concomitant TFV pro-drug. [FTC-TP] at 5 and 21 h post-dosing were >3.7 pmol/10^6^ cells in 48% and 27% of participants.

Phosphorylated metabolites, TFV-DP and FTC-TP, were quantifiable in >90% of foreskin samples over 21 h post-dose (Fig. 3A). Participants receiving F/TAF had approximately 2-fold higher [TFV-DP] [36.6 pmol/g (28.7–46.6)] in foreskin compared with those receiving F/TDF [18.5 pmol/g (14.9–23.4)], irrespective of the dose and time of sampling (GMR 1.95 (1.41, 2.68); Supplementary Table S6). The [TFV-DP] in foreskin increased following a 2 + 1 dose, but this was only significant for F/TDF. [FTC-TP] in foreskin tissue (Fig. 3C) were approximately 1 log higher than [TFV-DP] and there was no evidence of a difference between the two regimens [F/TDF = 326 pmol/g (262–405); F/TAF = 273 pmol/g (200–374); Supplementary Table S6]. Overall, foreskin [FTC-TP] were highest at the 5 h sampling interval (GMR for 21 h versus 5 h: 0.55 (0.38, 0.79)), whereas TFV-DP appeared to accumulate later, at 21 h post-dose (GMR 1.38 (1.00, 1.90)).

Plasma [TFV] were ∼94% lower (GMR 0.06 (0.05, 0.07), p < 0.0001) (p-value determined by likelihood ratio tests) in participants that received F/TAF (4.8 ng/mL (4.2–5.4)) compared to F/TDF (74.9 ng/mL (64.8–86.4)); whereas there was no evidence of a difference in plasma [FTC] (Supplementary Table S7). TAF was quantifiable in only 19% of plasma samples from participants receiving F/TAF [4.1 ng/mL (1.7–9.9) – both doses] and could not be detected at 21 h post-dose (<0.5 ng/mL). In foreskin, drugs were quantifiable in only 19% (TFV) and 62% (FTC) of samples. Among those that were detectable, no evidence of difference in drug tissue concentrations between the two regimens was observed (Supplementary Table S7).

p24 concentrations in foreskin explants were positively correlated with concentrations in PBMCs (Pearson's correlation coefficient, 0.53 and 0.46 for HVT and LVT challenge, respectively). Excluding control arm participants, the correlation remained for HVT challenge but was no longer seen for p24 concentrations following LVT challenge (0.46 and 0.12, respectively). We examined correlations between drug concentrations and inhibitory activity. Presence of drug was associated with decreased p24 concentrations in the treated arms compared to the control arm; however, among participants receiving PrEP, there was no evidence of correlation between p24 and drug concentrations in any compartment.

**Fig. 4:**
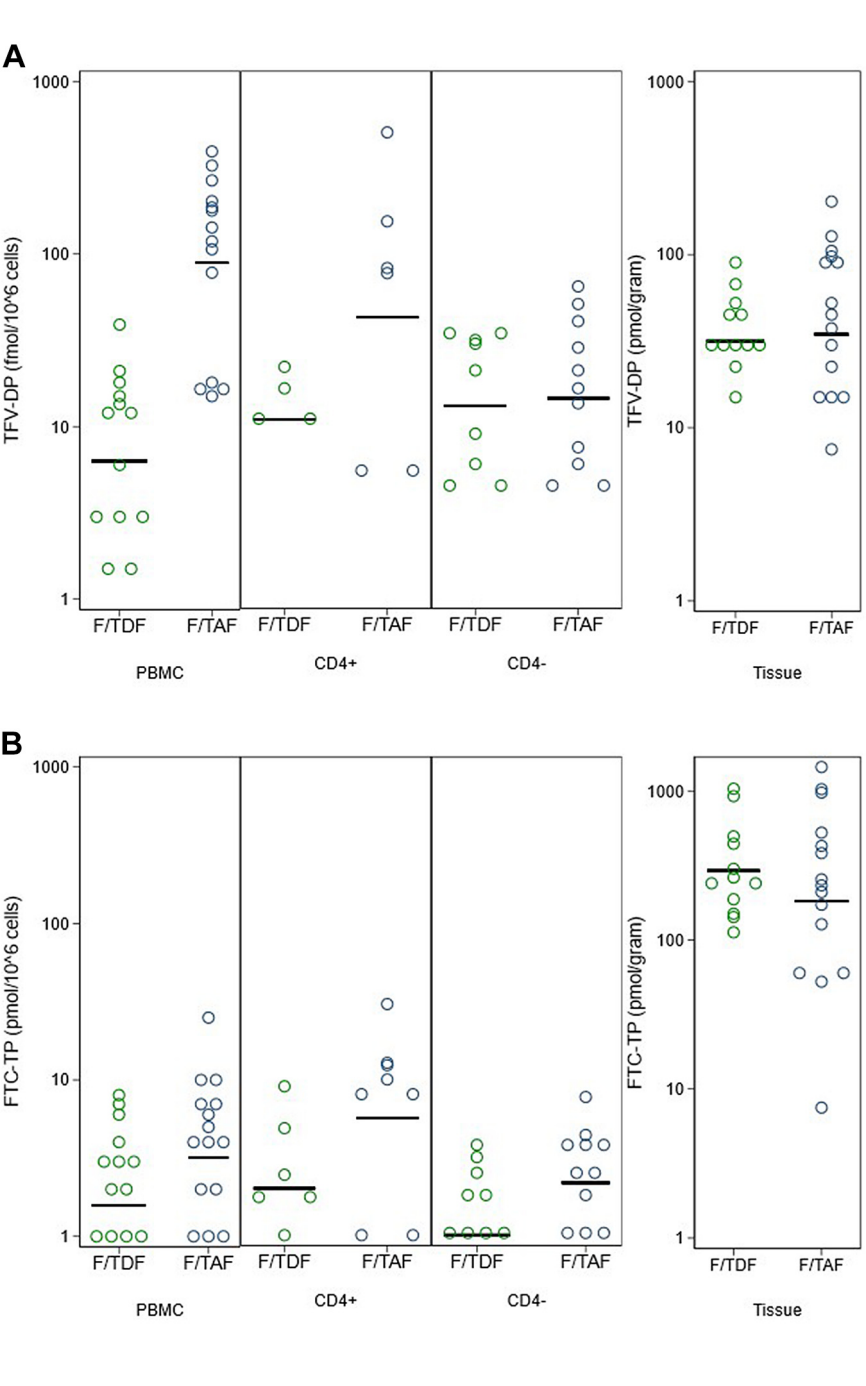
**Levels of (A) TFV-DP and (B) FTC-TP in PBMCs, CD4+ and CD4-cells, and tissue, among 28 participants who had CD4 cells isolated, stratified by trial arm. Data are from n** = **144 with each condition tested in biological triplicates**.

To assess the drug distribution within CD4+ HIV-target cells in foreskin, we conducted a case study with 28 foreskins from participants in the 2 + 1 dosing arms (Supplementary Table S8; Fig. 4A) (p-values determined by likelihood ratio tests). TFV-DP was detected in a total of 54% of CD4± samples analysed (36% CD4+; 71% CD4-). For those with detectable [TFV-DP] in CD4± fractions were similar between the TDF and TAF-based regimens, which reflected a lack of difference in [TFV-DP] seen in total foreskin tissue for this sub-set of individuals (p = 0.82; Fig. 4A). There was no evidence of a difference in the amount of TFV-DP/10^6^ CD4 cells in isolated CD4+ and CD4-fractions (CD4+ = 24.7 fmol/10^6^ cells; CD4- = 13.8 fmol/10^6^; p = 0.24). [TFV-DP] in CD4+ cells were significantly higher than in PBMC, but only for those dosed with F/TDF (p = 0.005). [FTC-TP] was highest in CD4+ cells (CD4+ = FTC-TP: 0.97 pmol/10^6^ cells; CD4- = FTC-TP: 0.32 pmol/10^6^ cells; p = 0.03), but CD4+ concentrations were 2.3-fold (95% CI: 1.5–3.3, p = 0.001) lower than observed in PBMCs in this sub-set of individuals. [TFV-DP] and [FTC-TP] in PBMCs and CD4-cells were strongly positively correlated (P < 0.01), whereas [TFV-DP] in isolated CD4+ fractions only correlated with levels observed in total foreskin tissue (P = 0.006) (Supplementary Table S9).

**Fig. 5:**
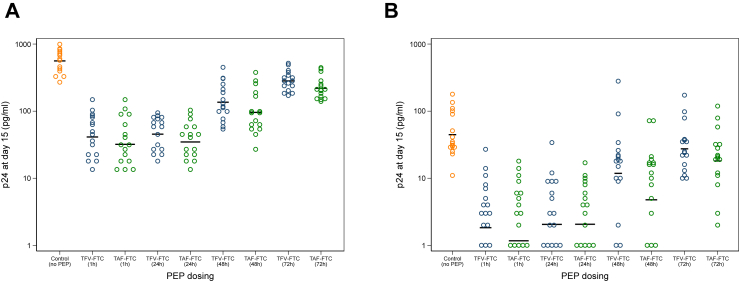
**TFV-FTC and TAF-FTC PEP activity against HIV-1**_**BaL**_**in foreskin explants, following (A) high titre challenge, (B) low titre challenge. Data are from n** = **16 with each condition tested in biological triplicates**.

Evaluation of TFV-FTC and TAF-FTC PEP activity in foreskin was assessed in additional explants cut from control arm foreskins. A significant reduction in p24 concentrations was measured in cultures dosed 1, 24, 48 or 72 h post-*ex vivo* challenge with HVT and LVT titres (all p ≤ 0.001; Fig. 5; Supplementary Table S10) (p-values determined by independent t-tests) with the effect of PEP reduced somewhat for dosing 48 h after exposure, and more dramatically for dosing 72 h after exposure. TFV-FTC was more potent than TAF-FTC if given at 1 h post exposure (GMR 1.29 (1.06–1.57)). For all other PEP times tested, TAF-FTC was more potent than TFV-FTC against HVT challenge (0.77 (0.59–1.00) for 24 h; 0.70 (0.56–0.89) for 48 h; 0.78 (0.69–0.87) for 72 h) (Supplementary Table S11).

## Discussion

CHAPS is a trial of on-demand PrEP in young men in Uganda and South Africa, evaluating F/TAF for PrEP and PEP timing and dosing to prevent HIV acquisition after *ex vivo* challenge of foreskin tissue with HIV-1_BaL_. We aimed to mimic a scenario for insertive sex. This trial measured drug concentrations in foreskin tissue and also HIV-target cells in foreskin.

The aim of the study was to provide guidance for dosing requirements and time to protection following starting PrEP. Our primary outcome demonstrated that an on-demand double dose regimen of F/TDF or F/TAF, given on a single occasion, is protective between 5 and 21 h prior to insertive intercourse, and that post-coital dosing conferred additional protection in PBMCs but not tissue. Our data did not show clear superiority of TAF to TDF in protecting foreskins against HIV infections, however the higher TFV-DP intracellular concentrations we report in those who received TAF suggest that protection may be of longer duration, or that the dosing requirements for on-demand F/TAF are somewhat lower than for F/TDF. The results of this study align with findings from the social science arm of CHAPS, which clearly showed that young men in Uganda, Zimbabwe and South Africa favoured on-demand PrEP and if possible, a pre-coital only PrEP regime.^10^

Both F/TDF and F/TAF were well tolerated and highly effective against *ex vivo* challenge of foreskin tissue and PBMCs with HIV-1_BaL_. The added value in protection provided by post-exposure challenge in PBMCs but not in foreskin tissue is highly relevant in the discussion of whether pre-coital dosing is sufficient for insertive sex. The higher p24 concentrations observed in PBMCs compared to those in foreskin explants reflects the greater susceptibility of the systemic compartment to HIV-1 but the similar protection observed between the compartments is reassuring. As similar protection was observed at both time points, we were unable to ascertain whether protection occurs first in PBMCs or foreskin tissue. The range of p24 concentrations we report in foreskin tissue fell between those previously reported in rectal and vaginal explant cultures.^22^ Our *ex vivo* challenge data suggest that the 2 + 1 PrEP regimen confers similar protection from HIV-1 infection as double dose PrEP and that the protection is sustained to at least 21 h after ingestion of the last PrEP dose. Our data are reassuring for young men who may not be able to predict the timing of sexual intercourse but would rather take a double dose of PrEP up to 21 h before having insertive sex.

PBMC metabolite exposures achieved in the F/TDF arms were comparable to those reported in the IPERGAY single dose pharmacokinetic study.^21^ By contrast, due to its preferential PBMC loading, 2 and 2 + 1 doses of F/TAF delivered high levels of TFV-DP that were both protective (>16 fmol/10^6^ cells) and similar to TFV-DP exposures achieved with three (33%) and five (67%) daily doses per week of F/TDF.^23^ [TFV-DP] and [FTC-TP] in foreskin were, in relation to other anatomical sites of HIV transmission, comparable to exposures previously reported in cervical and vaginal tissues.^24^^,^^25^ Increased TFV-DP loading in PBMCs with F/TAF is well established,^25^ and was replicated in this study. Interestingly, F/TAF also achieved higher [TFV-DP] in foreskin tissue than F/TDF; a finding that differs from the low TFV-DP detection rate previously seen in cervicovaginal tissues in participants treated with a single (25 mg) dose of TAF.^24^^,^^25^ This suggests that distribution of TAF and its conversion to TFV-DP in foreskin cannot be inferred from the vaginal compartment, re-enforcing the need to evaluate drug kinetics within distinct anatomical sites and patient populations of interest.

Intracellular TFV-DP accumulated in foreskin, which is potentially attributed to its long intracellular half-life in mucosal tissues (34–53 h in cervicovaginal tissues^26^). By contrast, FTC-TP exhibited a more rapid distribution in foreskin tissue but declined more rapidly. FTC-TP has previously been shown to decline in cervicovaginal tissues at a faster rate than TFV-DP.^27^ There was an added pharmacokinetic benefit of giving an extra dose the following day as participants receiving a 2 + 1 regimen had higher [TFV-DP] in foreskin, although mucosal FTC-TP levels were unchanged. Cottrell and colleagues found no evidence of dose proportionality, for either parent drug or metabolite, in the female genital tract and in colorectal tissues after giving escalating single doses of TDF and FTC; and in PBMC, TFV-DP concentrations increased proportionally but FTC-TP did not.^28^

This study has successfully measured phosphorylated antiretroviral nucleosides in CD4± cells isolated from foreskin tissue. Data from this small sub-study revealed that, unlike the PBMCs compartment, dosing with TAF did not confer preferential distribution of TFV-DP into foreskin HIV target cells. The lack of correlation between PBMCs and foreskin CD4+ fractions, further supports the notion that CD4+ cells are a distinct cellular sub-population. However, the small sample size, low detection rate, and high inter-subject variation mean that these preliminary data should be interpreted with caution.

A key strength of the trial design is the ability to assess several important PrEP variables including PrEP drug, PrEP dose and timing, and link tissue and cellular pharmacokinetics to pharmacodynamics. Unlike studies with HIV incidence as an outcome, no participants were exposed to additional risk of HIV due to group allocation.

Our study had some limitations. Firstly, this was an open-label study with blinding of arm allocation to laboratory staff but not participants or clinical personnel. Second, one foreskin specimen and two PBMC specimens from the control arm were not infected following *ex vivo* challenge with LVT, which affected the baseline control of infection; however, this reflects the donor-to-donor variability of *in vivo* susceptibility to HIV transmission. Third, HVTs routinely used for *ex vivo* challenge of foreskin explants, could not be used in PBMCs. Fourth, our sample size was relatively small, therefore we cannot rule out the possibility that there were differences between the PrEP trial arms that we were not powered to detect. Fifth, the study was not designed as a drug titration, prohibiting a more in-depth PK-PD correlation analysis among treated arms. The lack of PK-PD correlation could be due to activity of FTC which could be masking differences between TFV and TAF. The tissue explant model cannot appropriately metabolize the formulated version of TFV for oral dosing, hence, *ex vivo* dosing is performed with the base compound. Furthermore, tissue explants have limited capacity to demonstrate sterilizing protection, and progressively lose their architecture; however, CD4:CD8 T cell ratios and sufficient viability are maintained during culture to sustain viral replication.^29^ Finally, the location of the VMMC clinics in relation to the central processing laboratories meant it was not feasible to immediately section and “snap freeze” foreskin tissue at time of collection; instead, the entire foreskin was transported in media to the laboratory. This potentially accounts for the large proportion of tissue samples that had undetectable drug (81% TFV; 38% FTC), having previously demonstrated that drug loss from a foreskin tissue explant during culture in media is extensive.^18^

In an era when efficacy studies for HIV protection are large and prohibitively expensive, evidence from phase II studies of this nature will maximise the chance of identifying the most efficacious dosing strategy and filling data gaps around onset and offset of protection. This factorial design using the p24 protein as a surrogate endpoint can be used to efficiently select drug combinations and dosing regimens for testing in phase III, while obviating the unnecessary costs of futile regimens. The results from this study and future *ex vivo* HIV-challenge trials could inform future trials drug, dose and schedules that could offer the best protection against HIV.

## Contributors

CH and JS contributed equally to conceptualisation, formal analysis, investigation, methodology, resources, supervision, validation, writing – original draft; LE: conceptualisation, formal analysis, investigation, methodology, resources, supervision, validation, writing – original draft; LL: project administration, resources, supervision; DO, PS and RM: resources; ASS: project administration, resources; ADP, PN, TBS, GO, SM, AA, SDP and SP: investigation; BA: project administration, resources; KOH and CC: methodology; JS and HW: conceptualisation; SK: conceptualisation, supervision, validation, writing – original draft; FC: conceptualisation, resources, supervision, validation, writing – original draft; CMG: conceptualisation, resources, supervision; PK: conceptualisation, supervision; ELW: data curation, formal analysis, supervision, validation, writing – original draft; NM and JF contributed equally to conceptualisation, funding acquisition, resources, supervision, writing – original draft. All authors contributed to the writing– review & editing. All authors read and approved the final version of the manuscript. Participants enrolled in the CHAPS cohort provided their time and consented for the donation of resected foreskin to the study.`

## Data sharing statement

Deidentified participant data and a corresponding data dictionary will be available together with the study protocol, with publication and upon request to the corresponding author. This will be made available on LSHTM Data Compass repository. The Trial Management Group will approve data sharing requests.

## Declaration of interests

CH has received research grants from EDCTP, Vetenskapsrådet and Gilead Sciences. LE has received research grants from EDCTP, and Gilead Sciences. LL has received research grants from EDCTP, Gilead Sciences, Roche Diagnostic, DO has received research grants from EDCTP, AS has received research grants from EDCTP, AP has received research grants from EDCTP, PN has received research grants from EDCTP, PS has received research grants from EDCTP, DS has received research grants from EDCTP, RM has received research grants from EDCTP, BA has received research grants from EDCTP, SP has received research grants from EDCTP, CC is an employee of Gilead Sciences, JS has received research grants from EDCTP, HW has received research grants from EDCTP, SK has received research funding, speaker honoraria and consulting fees from EDCTP, Gilead Sciences, ViiV, Merck, GSK, and Ridgeback. FC has received research grants from EDCTP and Vetenskapsrådet. ELW has received grants from EDCTP, MRC, and NIH. CG has received research grants from EDCTP. PK has received research grants from EDCTP. EW has received research grants from EDCTP, NIH and MRC. NM has received research grants from EDCTP, and Gilead Sciences and provided unpaid advice and leadership in the DSMB and Setshaba boards. CC is a full-time employee of Gilead Sciences. All other authors declare no competing interests aside from the research grant received for this study by EDCTP.

## Appendix A Supplementary data

Supplementary material

Protocol The CHAPS Trial Version 1.2

CHAPS Study Team list
