# Supplementary material for: Dose finding study for on-demand HIV pre-exposure prophylaxis for insertive sex in sub-Saharan Africa: results from the CHAPS open label randomised controlled trial
Source: eBioMedicine. 2023 Jun 14;93:104648. doi: 10.1016/j.ebiom.2023.104648 (PMC10275696; doi:10.1016/j.ebiom.2023.104648)
Supplement: Supplementary material [file mmc1.docx]

**Supplementary Table 1. LC-MS method parameters for assays utilised in CHAPS clinical trial.**

| **Matrix** | **Analyte** | **Internal Standard (IS)** | **Specimen (volume)** | **Extraction Procedure** | **Chromatography** | **Calibration range** | **Equation** |
| --- | --- | --- | --- | --- | --- | --- | --- |
| Plasma | TAF  TFV  FTC | TAF-d5  TFV-d6  FTC-^13^C_15_N_2_ | EDTA plasma (100µL) | SPE  [SOLA SCX; 10mg/mL; Thermo Scientific] | Reverse phase (C_18_ column; Synergi Phenomenex ) | 0·5-500 ng/mL (TAF)  1-1000 ng/mL (TFV)  5-5000 ng/mL (FTC) | Linear 1/X^2^ |
| Foreskin tissue^1^ | TAF  TFV  FTC | TAF-d5  TFV-d6  FTC-^13^C_15_N_2_ | Pre-weighed tissue biopsy in methanol:water 20mM EDTA\|EGTA (70:30 v/v) (100µL) | SPE  [SOLA SCX; 10mg/mL; Thermo Scientific] | Reverse phase (C_18_ column; Synergi Phenomenex ) | 0·05-50 ng/sample (TAF)  0·1-100 ng/sample (TFV)  0·5-500 ng/sample (FTC) | Linear 1/X^2^ |
| Foreskin tissue | TFV-DP  FTC-TP | ^13^C-TDF-DP | Pre-weighed tissue biopsy in methanol:water 20mM EDTA\|EGTA (70:30 v/v) (100µL) | Acetonitrile:formic acid (98:2 v/v) followed by WAX SPE (Oasis WAX 30 mg/mL; Waters) | Weak Anion Exchange  (BioBasic AX; Thermo) | 0·036-25 pmol/sample | Linear 1/X^2^ |
| Cellular fractions (PBMC/CD4) | TFV-DP  FTC-TP^2^ | ^13^C-TDF-DP | Lysed cells in methanol:water 20mM EDTA\|EGTA (70:30 v/v) (100µL) | Acetonitrile | Weak Anion Exchange  (BioBasic AX; Thermo) | 0·03-12 pmol/sample (TFV-DP)  0·6-600 pmol/sample (FTC-TP) | Linear 1/X^2^ |

^1^ plasma calibrators and QC used as surrogate for tissue; ^2^ ^13^C-TDF-DP used as an IS for FTC-TP, a stable isotope labelled FTC-TP was not commercially available;

EDTA = Ethylenediaminetetraacetic Acid; EGTA = ethylene glycol-bis(β-aminoethyl ether)-N,N,N′,N′-tetraacetic acid; SPE = Solid Phase Extraction, WAX = Weak Anion Exchange

**Supplementary Table 2.** **Method validation parameters for LC-MS assays utilised in CHAPS clinical trial.**

| **Matrix** | **Analytes** | **Nominal level** | | **Inter-assay (%CV)** | | **Intra-assay (%CV)** | | **% Recovery & Matrix Effect** | | | |
| --- | --- | --- | --- | --- | --- | --- | --- | --- | --- | --- | --- |
|  |  |  |  | **Precision (%CV)** | **Accuracy**  **(%Bias)** | **Precision (%CV)** | **Accuracy**  **(%Bias)** | **PE [C:A]**  **(%CV)** | **Ext RE [C:B]**  **(%CV)** | **ME [B:A]**  **(%CV)** | **Analysis RE [C2:B2]**  **(%CV)** |
| Plasma  Foreskin tissue^1^ | TAF | 0·5 ng/mL | LLQ | 5·352 | 2·111 | 4·346 | -1·667 | - | - | - | - |
|  |  | 1·3 ng/mL | LQC | 14·588 | 12·003 | 1·108 | 7·543 | 97·237 | 97·907 | 99·316 | 116·161 |
|  |  | 42 ng/mL | MQC | 11·219 | 3·858 | 2·642 | 4·495 | 96·128 | 97·726 | 98·365 | 120·771 |
|  |  | 420 ng/mL | HQC | 4·813 | 6·567 | 4·779 | 7·136 | 104·028 | 110·520 | 94·126 | 123·480 |
|  | TFV | 1 ng/mL | LLQ | 13·815 | -5·9375 | 12·744 | -4·800 | - | - | - | - |
|  |  | 2·8 ng/mL | LQC | 12·993 | -3·034 | 12·798 | -0·604 | 45·599 | 49·995 | 91·206 | 86·934 |
|  |  | 85 ng/mL | MQC | 11·097 | -11·153 | 1·768 | -10·721 | 42·297 | 47·114 | 89·777 | 80·268 |
|  |  | 850 ng/mL | HQC | 5·573 | -7·052 | 7·465 | -8·213 | 50·423 | 56·964 | 88·516 | 82·404 |
|  | FTC | 5 ng/mL | LLQ | 12·782 | -5·577 | 7·126 | -0·400 | - | - | - | - |
|  |  | 13 ng/mL | LQC | 9·639 | -5·317 | 2·714 | -4·205 | 49·287 | 53·739 | 91·714 | 82·035 |
|  |  | 420 ng/mL | MQC | 10·514 | -4·368 | 4·336 | -2·691 | 52·016 | 56·454 | 92·139 | 83·719 |
|  |  | 4200 ng/mL | HQC | 5·663 | -4·695 | 8·280 | -2·434 | 62·777 | 68·395 | 91·785 | 83·036 |
| Foreskin tissue | TFV-DP | 0·04 pmol/sample | LLQ | 20·810 | 20·139 | 12·111 | 15·741 | - | - | - | - |
|  |  | 0·09 pmol/sample | LQC | 9·108 | 5·556 | 5·833 | 4·630 | 92·344 | 88·995 | 103·763 | 92·907 |
|  |  | 1·5 pmol/sample | MQC | 3·345 | -4·796 | 3·354 | -3·789 | 102·537 | 94·108 | 108·958 | 96·926 |
|  |  | 20 pmol/sample | HQC | 4·843 | 3·093 | 3·855 | 5·713 | 99·371 | 92·730 | 107·161 | 93·460 |
|  | FTC-TP | 0·04 pmol/sample | LLQ | 20·262 | 11·921 | 4·711 | -3·704 | - | - | - | - |
|  |  | 0·09 pmol/sample | LQC | 8·432 | 5·556 | 12·255 | -0·556 | 95·310 | 92·431 | 103·115 | 96·375 |
|  |  | 1·5 pmol/sample | MQC | 3·531 | -3·552 | 2·275 | -2·656 | 111·317 | 103·637 | 107·411 | 106·761 |
|  |  | 20 pmol/sample | HQC | 4·793 | 2·019 | 3·876 | 6·025 | 109·144 | 99·651 | 109·52 | 100·440 |
| Cellular fractions (PBMC/CD4) | TFV-DP | 1·8 pmol/sample | LQC | 4·920 | 3·880 | 2·720 | 1·240 | 101·953 | 97·530 | 104·535 | 100·404 |
|  |  | 3·6 pmol/sample | MQC | 3·340 | 0·700 | 2·030 | -1·140 | 108·260 | 99·913 | 108·354 | 100·108 |
|  |  | 9 pmol/sample | HQC | 3·350 | -1·870 | 2·770 | -0·250 | 103·010 | 99·915 | 103·098 | 101·498 |
|  | FTC-TP | 4·5 pmol/sample | LQC | 2·500 | -2·730 | 1·960 | -2·080 | 101·565 | 96·399 | 105·359 | 99·363 |
|  |  | 180 pmol/sample | MQC | 3·240 | 0·270 | 1·570 | -1·180 | 106·683 | 101·873 | 104·721 | 102·184 |
|  |  | 450 pmol/sample | HQC | 2·420 | -1·770 | 2·750 | -1·160 | 103·455 | 100·928 | 102·504 | 101·973 |

A: Peak area of aqueous mobile phase solutions without matrix and without extraction; Analysis RE: Analysis recovery (internal standard [IS]-normalized) ratio of the mean peak area ratio of the analyte spiked prior to extraction (C2) to the mean peak area ratio of the analyte spiked after extraction (B2) × 100; B: Peak area of analyte spiked after extraction; B2: Ratio of the peak area of analyte and IS spiked after extraction; C: Peak area of analyte spiked prior to extraction; C2: Ratio of the peak area of analyte and IS spiked prior to extraction; Ext RE: Extraction yield calculated as the ratio of the mean peak area of the analyte spiked prior to extraction (C) to the mean peak area of the analyte spiked after extraction (B) × 100; ME: Matrix effect expressed as the ratio of the mean peak area of the analyte spiked after extraction (B) to the mean peak area of an equivalent concentration of analyte in mobile phase (A) × 100; PE: Process efficiency expressed as the ratio of the mean peak area of the analyte spiked prior to extraction (C) to the mean peak area of the same analyte standard (A) × 100.

LLQ: lower limit of quantification; LQC: low quality control; MQC: medium quality control; HQC: high quality control.

**Supplementary Table 3. Listing of adverse events.**

| **Type of adverse event** | **Total number of adverse events** | **Number of adverse events classified as mild** | **Number of adverse events classified as moderate** | **Number of adverse events classified as severe** |
| --- | --- | --- | --- | --- |
| Sexually transmitted infection | 9 | 9 | 0 | 0 |
| Bleeding during circumcision | 3 | 3 | 0 | 0 |
| Bleeding after circumcision | 1 | 0 | 0 | 1 |
| Pain during circumcision | 2 | 1 | 1 | 0 |
| Pain after circumcision | 3 | 3 | 0 | 0 |
| Post-operative infection | 2 | 2 | 0 | 0 |
| Syncope | 1 | 0 | 1 | 0 |
| **Total** | **21** | **18** | **2** | **1** |

**Supplementary Table 4. p24 outcomes in tissue and PBMCs, comparisons between trial arms.**

|  |  | **High titre challenge** | | | | **Low titre challenge** | | | |
| --- | --- | --- | --- | --- | --- | --- | --- | --- | --- |
| **p24 at day 15, tissue** | | | | | | | | | |
| **Reference group** | **Comparator group** | **GM^1^ (95% CI), ref group** | **GM^1^ (95% CI), comparator** | **GMR^2^ (95% CI)** | **p-value** | **GM^1^ (95% CI), ref group** | **GM^1^ (95% CI), comparator** | **GMR^2^ (95% CI)** | **p-value** |
| Effect of any PrEP *versus* no PrEP | | | | | | | | | |
| Control arm | Any PrEP | 560 (454, 691) | 41·3 (36·1, 47·4) | 0·07 (0·05, 0·11) | <0·0001 | 44·6 (29·6, 67·0) | 2·99 (2·34, 3·83) | 0·07 (0·03, 0·14) | <0·0001 |
| Overall effects of drug, dosage and interval | | | | | | | | | |
| F/TDF (all) | F/TAF (all) | 37·3 (30·9, 45·1) | 45·8 (37·6, 55·8) | 1·23 (0·93, 1·61) | 0·1 | 2·91 (2·09, 4·05) | 3·08 (2·12, 4·47) | 1·06 (0·65, 1·74) | 0·8 |
| 2 tabs (both drugs) | 2+1 tabs (both drugs) | 44·9 (37·5, 53·7) | 38·1 (30·9, 46·9) | 0·85 (0·65, 1·11) | 0·2 | 2·85 (1·87, 4·34) | 3·14 (2·39, 4·11) | 1·10 (0·67, 1·80) | 0·7 |
| 5 hours (all regimens) | 21 hours (all regimens) | 39·2 (32·0, 48·0) | 43·6 (36·2, 52·6) | 1·11 (0·85, 1·46) | 0·4 | 2·45 (1·60, 3·76) | 3·65 (2·82, 4·69) | 1·48 (0·91, 2·43) | 0·1 |
| Effects of dosage, separately for each drug | | | | | | | | | |
| F/TDF, 2 tabs | F/TDF, 2 + 1 tabs | 41·9 (32·6, 53·8) | 33·3 (24·8, 44·7) | 0·80 (0·54, 1·16) | 0·2 | 2·61 (1·43, 4·77) | 3·23 (2·35, 4·45) | 1·24 (0·63, 2·41) | 0·5 |
| F/TAF, 2 tabs | F/TAF, 2 +1 tabs | 48·1 (36·7, 63·0) | 43·6 (32·1, 59·1) | 0·91 (0·61, 1·35) | 0·6 | 3·11 (1·68, 5·78) | 3·04 (1·93, 4·80) | 0·98 (0·46, 2·08) | 0·9 |
| Effects of interval, separately for each drug and dosage | | | | | | | | | |
| F/TDF, 2 tabs (5h) | F/TDF, 2 tabs (21h) | 39·8 (27·1, 58·3) | 44·1 (30·5, 63·6) | 1·11 (0·67, 1·84) | 0·7 | 1·88 (0·61, 5·77) | 3·64 (2·09, 6·35) | 1·94 (0·58, 6·43) | 0·3 |
| F/TDF, 2+1 tabs (5h) | F/TDF, 2+1 tabs (21h) | 30·2 (19·7, 46·6) | 36·7 (23·5, 57·3) | 1·21 (0·67, 2·20) | 0·5 | 2·88 (1·91, 4·35) | 3·63 (2·13, 6·19) | 1·26 (0·66, 2·40) | 0·5 |
| F/TAF, 2 tabs (5h) | F/TAF, 2 tabs (21h) | 48·2 (30·8, 75·3) | 48·1 (33·5, 68·9) | 0·98 (0·58, 1·73) | 1·0 | 2·59 (0·84, 7·97) | 3·75 (1·93, 7·27) | 1·45 (0·41, 5·06) | 0·6 |
| F/TAF, 2+1 tabs (5h) | F/TAF, 2+1 tabs (21h) | 40·7 (25·3, 65·4) | 46·7 (30·1, 72·3) | 1·15 (0·62, 2·13) | 0·6 | 2·59 (1·10, 6·11) | 3·57 (2·32, 5·50) | 1·38 (0·55, 3·45) | 0·5 |
| **p24 AUC, tissue** | | | | | | | | | |
| Effect of any PrEP *versus* no PrEP | | | | | | | | | |
| Control arm | Any PrEP | 4731 (3838, 5832) | 1015 (930, 1108) | 0·21 (0·17, 0·28) | <0·0001 | 350 (239, 513) | 73·2 (64·3, 83·3) | 0·21 (0·14, 0·31) | <0·0001 |
| Overall effects of drug, dosage and interval | | | | | | | | | |
| F/TDF(all) | F/TAF (all) | 971 (866, 1089) | 1061 (927, 1213) | 1·09 (0·92, 1·30) | 0·3 | 72·3 (60·3, 86·7) | 74·1 (61·3, 89·6) | 1·02 (0·79, 1·33) | 0·9 |
| 2 tabs (both drugs) | 2+1 tabs (both drugs) | 1031 (908, 1171) | 1000 (884, 1131) | 0·97 (0·81, 1·16) | 0·7 | 73·3 (60·0, 89·5) | 73·2 (61·7, 86·8) | 1·00 (0·77, 1·30) | 1·0 |
| 5 hours (all regimens) | 21 hours (all regimens) | 1047 (929, 1180) | 984 (864, 1122) | 0·94 (0·79, 1·12) | 0·5 | 71·9 (60·1, 85·9) | 74·6 (61·5, 90·4) | 1·04 (0·80, 1·35) | 0·8 |
| Effects of dosage, separately for each drug | | | | | | | | | |
| F/TDF, 2 tabs | F/TDF, 2 + 1 tabs | 1027 (873, 1208) | 919 (777, 1087) | 0·89 (0·71, 1·13) | 0·3 | 68·3 (52·0, 89·8) | 76·6 (59·5, 98·6) | 1·12 (0·78, 1·61) | 0·5 |
| F/TAF, 2 tabs | F/TAF, 2 +1 tabs | 1035 (843, 1271) | 1087 (904, 1308) | 1·05 (0·80, 1·38) | 0·7 | 78·5 (57·9, 107) | 69·9 (54·9, 89·1) | 0·89 (0·61, 1·30) | 0·6 |
| Effects of interval, separately for each drug and dosage | | | | | | | | | |
| F/TDF, 2 tabs (5h) | F/TDF, 2 tabs (21h) | 1057 (856, 1306) | 998 (759, 1310) | 0·94 (0·68, 1·31) | 0·7 | 66·0 (45·3, 96·0) | 70·7 (45·5, 110) | 1·07 (0·62, 1·87) | 0·8 |
| F/TDF, 2+1 tabs (5h) | F/TDF, 2+1 tabs (21h) | 870 (679, 1116) | 970 (753, 1250) | 1·12 (0·79, 1·57) | 0·5 | 74·2 (54·8, 100) | 79·0 (50·8, 123) | 1·07 (0·64, 1·78) | 0·8 |
| F/TAF, 2 tabs (5h) | F/TAF, 2 tabs (21h) | 1115 (847, 1468) | 960 (688, 1340) | 0·86 (0·57, 1·30) | 0·5 | 78·3 (49·9, 123) | 78·8 (49·4, 126) | 1·01 (0·54, 1·88) | 1·0 |
| F/TAF, 2+1 tabs (5h) | F/TAF, 2+1 tabs (21h) | 1170 (888, 1541) | 1010 (768, 1329) | 0·86 (0·60, 1·25) | 0·4 | 69·7 (46·3, 105) | 70·2 (51·4, 95·8) | 1·01 (0·62, 1·65) | 1·0 |
| **p24 slope, tissue** | | | | | | | | | |
| Effect of any PrEP *versus* no PrEP | | | | | | | | | |
| Control arm | Any PrEP | 10·7 (1·5, 19·9) | -9·5 (-12·4, -6·5) | -20·2 (-29·0, -11·3) | <0·0001 | 6·6 (0·6, 12·5) | -17·7 (-25·7, -9·6) | -24·2 (-47·1, -1·3) | 0·04 |
| Overall effects of drug, dosage and interval | | | | | | | | | |
| F/TDF(all) | F/TAF (all) | -9·6 (-12·4, -6·5) | -9·3 (-12·7, -5·9) | 0·3 (-5·7, 6·2) | 0·9 | -22·5 (-36·4, -8·6) | -12·8 (-21·2, -4·5) | 9·7 (-6·4, 25·8) | 0·2 |
| 2 tabs (both drugs) | 2+1 tabs (both drugs) | -11·6 (-16·6, -6·5) | -7·4 (-10·4, -4·3) | -4·2 (-1·6, 10·0) | 0·2 | -17·5 (-29·6, -5·3) | -17·9 (-28·7, -7·0) | -0·4 (-16·5, 15·7) | 0·9 |
| 5 hours (all regimens) | 21 hours (all regimens) | -6·7 (-9·8, -3·6) | -12·2 (-17·3, -7·2) | -5·5 (-11·4, 0·3) | 0·06 | -21·7 (-35·0, -8·3) | -13·6 (-22·9, -4·4) | 8·0 (-8·1, 24·2) | 0·3 |
| Effects of dosage, separately for each drug | | | | | | | | | |
| F/TDF, 2 tabs | F/TDF, 2 + 1 tabs | -14·2 (-23·7, -4·8) | -5·0 (-7·7, -2·2) | 9·3 (-0·4, 18·9) | 0·06 | -24·7 (-43·8, -5·7) | -20·3 (-41·7, 1·1) | 4·5 (-23·6, 32·5) | 0·8 |
| F/TAF, 2 tabs | F/TAF, 2 +1 tabs | -8·9 (-13·1, -4·7) | -9·8 (-15·4, -4·2) | -0·9 (-7·7, 6·0) | 0·8 | -10·2 (-26·1, 5·8) | -15·4 (-21·8, -9·0) | -5·3 (-22·1, 11·5) | 0·5 |
| Effects of interval, separately for each drug and dosage | | | | | | | | | |
| F/TDF, 2 tabs (5h) | F/TDF, 2 tabs (21h) | -7·9 (-13·7, -2·2) | -20·6 (-39·2, -1·9) | -12·7 (-31·3, 6·0) | 0·2 | -24·5 (-45·7, -3·4) | -24·9 (-59·4, 9·5) | -0·4 (-39·1, 38·3) | 1·0 |
| F/TDF, 2+1 tabs (5h) | F/TDF, 2+1 tabs (21h) | -5·1 (-9·4, -0·8) | -4·8 (-8·7, -1·0) | 0·3 (-5·3, 5·8) | 0·9 | -27·6 (-72·2, 17·0) | -12·9 (-19·7, -6·1) | 14·7 (-28·5, 57·9) | 0·5 |
| F/TAF, 2 tabs (5h) | F/TAF, 2 tabs (21h) | -4·8 (-9·9, 0·3) | -13·0 (-19·5, -6·5) | -8·2 (-16·2, -0·3) | 0·04 | -20·6 (-50·2, 9·0) | 0·2 (-13·8, 14·3) | 20·9 (-10·6, 52·3) | 0·2 |
| F/TAF, 2+1 tabs (5h) | F/TAF, 2+1 tabs (21h) | -9·0 (-19·1, 1·1) | -10·5 (-16·8, -4·3) | -1·5 (-12·9, 9·9) | 0·8 | -13·9 (-22·2, -5·5) | -17·0 (-27·7, -6·3) | -3·1 (-16·1, 9·9) | 0·6 |
| **p24 at day 15, PBMCs** | | | | | | | | | |
| Effect of any PrEP *versus* no PrEP | | | | | | | | | |
| Control arm | Any PrEP | 9579 (7876, 11650) | 2124 (1646, 2740) | 0·22 (0·11, 0·45) | <0·0001 | 1402 (724, 2717) | 36·3 (29·3, 44·9) | 0·03 (0·01, 0·05) | <0·0001 |
| Overall effects of drug, dosage and interval | | | | | | | | | |
| F/TDF(all) | F/TAF (all) | 2495 (1790, 3479) | 1821 (1234, 2689) | 0·73 (0·44, 1·21) | 0·2 | 36·9 (27·0, 50·4) | 35·7 (26·4, 48·3) | 0·97 (0·63, 1·49) | 0·9 |
| 2 tabs (both drugs) | 2+1 tabs (both drugs) | 1943 (1330, 2839) | 2331 (1645, 3303) | 1·22 (0·74, 2·03) | 0·4 | 35·9 (26·1, 49·3) | 36·7 (27·3, 49·3) | 1·03 (0·67, 1·58) | 0·9 |
| 5 hours (all regimens) | 21 hours (all regimens) | 2600 (1925, 3513) | 1751 (1163, 2638) | 0·66 (0·40, 1·10) | 0·1 | 39·3 (28·9, 53·5) | 33·6 (24·8, 45·6) | 0·85 (0·55, 1·32) | 0·5 |
| Effects of dosage, separately for each drug | | | | | | | | | |
| F/TDF, 2 tabs | F/TDF, 2 + 1 tabs | 2584 (1696, 3937) | 2400 (1382, 4168) | 0·93 (0·47, 1·82) | 0·8 | 35·6 (22·8, 55·7) | 38·4 (24·2, 60·9) | 1·08 (0·57, 2·02) | 0·8 |
| F/TAF, 2 tabs | F/TAF, 2 +1 tabs | 1461 (770, 2772) | 2270 (1421, 3627) | 1·55 (0·71, 3·38) | 0·3 | 36·2 (22·5, 58·1) | 35·3 (23·6, 52·8) | 0·98 (0·53, 1·80) | 0·9 |
| Effects of interval, separately for each drug and dosage | | | | | | | | | |
| F/TDF, 2 tabs (5h) | F/TDF, 2 tabs (21h) | 2822 (1758, 4532) | 2380 (1133, 4996) | 0·84 (0·36, 1·98) | 0·7 | 36·1 (20·9, 62·3) | 35·1 (16·3, 75·6) | 0·97 (0·39, 2·42) | 1·0 |
| F/TDF, 2+1 tabs (5h) | F/TDF, 2+1 tabs (21h) | 2267 (998, 5150) | 2521 (1084, 5862) | 1·11 (0·36, 3·44) | 0·8 | 43·4 (18·1, 104) | 34·5 (20·2, 58·8) | 0·79 (0·31, 2·04) | 0·6 |
| F/TAF, 2 tabs (5h) | F/TAF, 2 tabs (21h) | 2334 (1168, 4667) | 886 (285, 2759) | 0·38 (0·11, 1·33) | 0·1 | 35·2 (18·3, 67·6) | 37·2 (17·1, 81·2) | 1·06 (0·40, 2·79) | 0·9 |
| F/TAF, 2+1 tabs (5h) | F/TAF, 2+1 tabs (21h) | 2037 (1594, 5747) | 1734 (840, 3580) | 0·57 (0·23, 1·45) | 0·2 | 44·2 (23·1, 84·7) | 28·5 (16·7, 48·9) | 0·65 (0·29, 1·44) | 0·3 |
| **p24 AUC, PBMCs** | | | | | | | | | |
| Effect of any PrEP *versus* no PrEP | | | | | | | | | |
| Control arm | Any PrEP | 94149 (79362, 111690) | 42315 (35714, 50136) | 0·45 (0·28, 0·72) | 0·001 | 11844 (7266, 19306) | 1800 (1638, 1978) | 0·15 (0·11, 0·21) | <0·0001 |
| Overall effects of drug, dosage and interval | | | | | | | | | |
| F/TDF(all) | F/TAF (all) | 48222 (38713, 60067) | 37363 (28842, 48401) | 0·77 (0·55, 1·09) | 0·1 | 1837 (1597, 2114) | 1765 (1549, 2011) | 0·96 (0·79, 1·16) | 0·7 |
| 2 tabs (both drugs) | 2+1 tabs (both drugs) | 41349 (32615, 52422) | 43353 (33780, 55639) | 1·05 (0·75, 1·48) | 0·8 | 1810 (1553, 2108) | 1790 (1599, 2003) | 0·99 (0·82, 1·20) | 0·9 |
| 5 hours (all regimens) | 21 hours (all regimens) | 47319 (38288, 58480) | 38042 (29164, 49621) | 0·80 (0·57, 1·12) | 0·2 | 1889 (1639, 2179) | 1718 (1513, 1952) | 0·91 (0·75, 1·10) | 0·3 |
| Effects of dosage, separately for each drug | | | | | | | | | |
| F/TDF, 2 tabs | F/TDF, 2 + 1 tabs | 54097 (41986, 69701) | 42645 (29256, 62163) | 0·79 (0·55, 1·22) | 0·2 | 1857 (1456, 2369) | 1816 (1568, 2103) | 0·98 (0·74, 1·30) | 0·9 |
| F/TAF, 2 tabs | F/TAF, 2 +1 tabs | 31872 (21575, 47082) | 44026 (30979, 62567) | 1·38 (0·82, 2·31) | 0·2 | 1765 (1444, 2157) | 1765 (1478, 2109) | 1·00 (0·77, 1·30) | 1·0 |
| Effects of interval, separately for each drug and dosage | | | | | | | | | |
| F/TDF, 2 tabs (5h) | F/TDF, 2 tabs (21h) | 53376 (43394, 65654) | 54782 (33773, 88859) | 1·03 (0·61, 1·72) | 0·9 | 1964 (1381, 2792) | 1763 (1211, 2566) | 0·90 (0·55, 1·47) | 0·7 |
| F/TDF, 2+1 tabs (5h) | F/TDF, 2+1 tabs (21h) | 45229 (25124, 81420) | 40366 (23242, 70107) | 0·89 (0·41, 1·92) | 0·8 | 1774 (1393, 2261) | 1856 (1516, 2272) | 1·05 (0·78, 1·41) | 0·8 |
| F/TAF, 2 tabs (5h) | F/TAF, 2 tabs (21h) | 42702 (26736, 68204) | 23788 (12479, 45345) | 0·56 (0·26, 1·20) | 0·1 | 1866 (1369, 2543) | 1669 (1249, 2230) | 0·89 (0·60, 1·34) | 0·6 |
| F/TAF, 2+1 tabs (5h) | F/TAF, 2+1 tabs (21h) | 48819 (29107, 81879) | 39961 (23444, 68115) | 0·82 (0·40, 1·67) | 0·6 | 1954 (1411, 2705) | 1605 (1327, 1941) | 0·82 (0·5, 1·17) | 0·3 |
| **p24 slope, PBMCs** | | | | | | | | | |
| Effect of any PrEP *versus* no PrEP | | | | | | | | | |
| Control arm | Any PrEP | 7·9 (4·7, 11·1) | -2·6 (-11·1, 6·0) | -10·5 (-34·3, 13·3) | 0·4 | 3·8 (-9·7, 17·2) | -3·6 (-5·9, -1·3) | -7·4 (-15·2, 0·4) | 0·06 |
| Overall effects of drug, dosage and interval | | | | | | | | | |
| F/TDF(all) | F/TAF (all) | -3·5 (-11·5, 4·5) | -1·7 (-16·8, 13·4) | 1·8 (-15·4, 18·9) | 0·8 | -3·7 (-7·6, 0·2) | -3·5 (-6·2, -0·7) | 0·3 (-4·4, 4·9) | 0·9 |
| 2 tabs (both drugs) | 2+1 tabs (both drugs) | 3·0 (-13·1, 19·1) | -8·5 (-13·5, -3·5) | -11·6 (-28·7, 5·6) | 0·2 | -3·0 (-6·6, 0·5) | -4·2 (-7·3, -1·1) | -1·2 (-5·9, 3·5) | 0·6 |
| 5 hours (all regimens) | 21 hours (all regimens) | -6·5 (-12·2, -0·8) | 1·1 (-14·8, 17·1) | 7·7 (-9·4, 24·9) | 0·4 | -4·6 (-7·6, -1·6) | -2·6 (-6·2, 1·0) | 2·0 (-2·7, 6·7) | 0·4 |
| Effects of dosage, separately for each drug | | | | | | | | | |
| F/TDF, 2 tabs | F/TDF, 2 + 1 tabs | 2·6 (-11·4, 16·7) | -10·0 (-17·2, -2·8) | -12·6 (-28·4, 3·2) | 0·1 | -2·0 (-7·8, 3·7) | -5·6 (-11·0, -0·1) | -3·5 (-11·3, 4·3) | 0·4 |
| F/TAF, 2 tabs | F/TAF, 2 +1 tabs | 3·4 (-26·2, 33·1) | -7·0 (-14·4, 0·3) | -10·5 (-40·9, 19·9) | 0·5 | -4·0 (-8·5, 0·5) | -2·9 (-6·2, 0·4) | 1·1 (-4·4, 6·6) | 0·7 |
| Effects of interval, separately for each drug and dosage | | | | | | | | | |
| F/TDF, 2 tabs (5h) | F/TDF, 2 tabs (21h) | -1·5 (-15·1, 12·0) | 6·5 (-19·3, 32·3) | 8·0 (-20·5, 36·5) | 0·6 | -5·4 (-8·8, -2·1) | 1·2 (-9·9, 12·3) | 6·6 (-4·8, 18·1) | 0·3 |
| F/TDF, 2+1 tabs (5h) | F/TDF, 2+1 tabs (21h) | -7·3 (-20·9, 6·3) | -12·5 (-20·1, -4·9) | -5·2 (-19·7, 9·4) | 0·5 | -4·2 (-15·6, 7·2) | -6·8 (-10·9, -2·7) | -2·6 (-13·8, 8·6) | 0·6 |
| F/TAF, 2 tabs (5h) | F/TAF, 2 tabs (21h) | -14·1 (-21·8, -6·4) | 20·9 (-40·1, 82·0) | 35·1 (-23·9, 94·0) | 0·2 | -6·7 (-10·0, -3·3) | -1·3 (-10·1, 7·4) | 5·3 (-3·6, 14·3) | 0·2 |
| F/TAF, 2+1 tabs (5h) | F/TAF, 2+1 tabs (21h) | -2·5 (-16·4, 11·4) | -11·3 (-18·3, -4·2) | -8·7 (-23·4, 5·9) | 0·2 | -2·1 (-8·1, 4·0) | -3·7 (-7·5, 0·1) | -1·7 (-8·4, 5·1) | 0·6 |

^1^For slope outcomes, figures shown are arithmetic means; ^2^For slope outcomes, figures shown are arithmetic mean difference.

**Supplementary Table 5. Effect of additional *ex vivo* dosing^a^ on p24 outcomes in tissue and PBMCs, overall and stratified by trial arm.**

|  | **High titre challenge** | | | | **Low titre challenge** | | | |
| --- | --- | --- | --- | --- | --- | --- | --- | --- |
| **Tissue, p24 at day 15** | | | | | | | | |
| **Analysis group** | **No *ex vivo* dose,**  **GM^1^ (95% CI)** | ***Ex vivo* dose,**  **GM^1^ (95% CI)** | **GMR^2^**  **(95% CI)** | **p-value** | **No ex vivo dose, GM^1^ (95% CI)** | ***Ex vivo* dose,**  **GM^1^ (95% CI)** | **GMR^2^**  **(95% CI)** | **p-value** |
| All trial arms combined | 41·3 (36·1, 47·4) | 37·9 (32·8, 43·8) | 0·92 (0·83, 1·01) | 0·09 | 3·0 (2·3, 3·8) | 2·9 (2·3, 3·8) | 0·98 (0·91, 1·07) | 0·7 |
| F/TDF, 2 tabs (5h) | 39·8 (27·1, 58·3) | 37·5 (22·9, 61·5) | 0·94 (0·67, 1·32) | 0·7 | 1·9 (0·6, 5·8) | 2·1 (0·7, 6·2) | 1·10 (0·78, 1·56) | 0·6 |
| F/TDF, 2 tabs (21h) | 44·1 (30·5, 63·6) | 41·5 (29·6, 58·1) | 0·94 (0·75, 1·19) | 0·6 | 3·6 (2·1, 6·3) | 3·9 (2·1, 7·0) | 1·06 (0·88, 1·29) | 0·5 |
| F/TDF, 2+1 tabs (5h) | 30·2 (19·7, 46·6) | 28·3 (18·2, 44·1) | 0·94 (0·78, 1·13) | 0·5 | 2·9 (1·9, 4·3) | 2·5 (1·6, 3·9) | 0·88 (0·69, 1·12) | 0·3 |
| F/TDF, 2+1 tabs (21h) | 46·7 (23·5, 57·3) | 31·5 (20·9, 47·6) | 0·86 (0·63, 1·18) | 0·3 | 3·6 (2·1, 6·2) | 3·5 (1·8, 6·7) | 0·97 (0·75, 1·24) | 0·8 |
| F/TAF, 2 tabs (5h) | 48·2 (30·8, 75·3) | 44·2 (26·7, 73·2) | 0·92 (0·73, 1·15) | 0·4 | 2·6 (0·8, 8·0) | 2·2 (0·7, 6·8) | 0·84 (0·63, 1·12) | 0·2 |
| F/TAF, 2 tabs (21h) | 48·1 (33·5, 68·9) | 39·4 (23·4, 66·3) | 0·82 (0·46, 1·45) | 0·5 | 3·7 (1·9, 7·3) | 3·5 (1·7, 7·2) | 0·93 (0·73, 1·18) | 0·5 |
| F/TAF, 2+1 tabs (5h) | 40·7 (25·3, 65·4) | 38·2 (25·8, 56·4) | 0·94 (0·77, 1·15) | 0·5 | 2·6 (1·1, 6·1) | 2·7 (1·1, 6·5) | 1·04 (0·93, 1·16) | 0·4 |
| F/TAF, 2+1 tabs (21h) | 46·7 (30·1, 72·3) | 46·3 (30·6, 69·9) | 0·99 (0·80, 1·23) | 0·9 | 3·6 (2·3, 5·5) | 3·9 (2·3, 6·6) | 1·08 (0·83, 1·41) | 0·6 |
| **Tissue, p24 area under the curve** | | | | | | | | |
| All trial arms combined | 1015 (930, 1107) | 1048 (964, 1141) | 1·03 (0·97, 1·10) | 0·3 | 73·2 (64·3, 83·3) | 75·4 (66·0, 86·1) | 1·03 (0·96, 1·11) | 0·4 |
| F/TDF, 2 tabs (5h) | 1057 (856, 1306) | 1149 (948, 1391) | 1·09 (0·91, 1·29) | 0·3 | 66·0 (45·3, 96·0) | 67·3 (43·5, 104) | 1·02 (0·85, 1·22) | 0·8 |
| F/TDF, 2 tabs (21h) | 998 (759, 1310) | 1119 (923, 1359) | 1·12 (0·98, 1·29) | 0·1 | 70·7 (45·5, 110) | 77·9 (51·4, 118) | 1·10 (1·00, 1·21) | 0·05 |
| F/TDF, 2+1 tabs (5h) | 870 (679, 1116) | 900 (698, 1161) | 1·03 (0·83, 1·29) | 0·8 | 74·2 (54·8, 100) | 57·8 (45·6, 73·2) | 0·78 (0·57, 1·06) | 0·1 |
| F/TDF, 2+1 tabs (21h) | 970 (753, 1250) | 969 (638, 1472) | 1·00 (0·82, 1·21) | 1·0 | 79·0 (50·8, 123) | 95·6 (57·9, 158) | 1·21 (1·02, 1·44) | 0·03 |
| F/TAF, 2 tabs (5h) | 1115 (847, 1468) | 1024 (782, 1341) | 0·92 (0·81, 1·04) | 0·2 | 78·3 (49·9, 123) | 76·0 (51·9, 111) | 0·97 (0·83, 1·13) | 0·7 |
| F/TAF, 2 tabs (21h) | 960 (688, 1340) | 1046 (824, 1327) | 1·09 (0·83, 1·42) | 0·5 | 78·8 (49·4, 126) | 75·3 (44·9, 126) | 0·95 (0·73, 1·25) | 0·7 |
| F/TAF, 2+1 tabs (5h) | 1170 (888, 1541) | 1090 (850, 1397) | 0·93 (0·81, 1·07) | 0·3 | 69·7 (46·3, 105) | 74·7 (52·1, 107) | 1·07 (0·98, 1·18) | 0·1 |
| F/TAF, 2+1 tabs (21h) | 1010 (768, 1329) | 1117 (919, 1356) | 1·11 (0·93, 1·31) | 0·2 | 70·2 (51·4, 95·8) | 84·1 (59·1, 120) | 1·20 (0·87, 1·65) | 0·3 |
| **Tissue, p24 slope** | | | | | | | | |
| All trial arms combined | -9·5 (-12·4, -6·5) | -5·0 (-6·8, -3·1) | 4·5 (1·4, 7·6) | 0·005 | -17·7 (-25·7, -9·6) | -9·6 (-13·9, -5·4) | 8·0 (-0·05, 16·1) | 0·05 |
| F/TDF, 2 tabs (5h) | -7·9 (13·7, -2·2) | -4·4 (-8·2, -0·6) | 3·5 (-0·8, 7·8) | 0·1 | -24·5 (-45·7, -3·4) | -15·3 (-25·5, -5·1) | 9·3 (-9·5, 28·1) | 0·3 |
| F/TDF, 2 tabs (21h) | -20·6 (-39·2, -1·9) | -6·8 (-11·4, -2·2) | 13·8 (-5·5, 33·0) | 0·2 | -24·9 (-59·4, 9·5) | -5·0 (-19·3, 9·3) | 19·9 (-13·7, 53·5) | 0·2 |
| F/TDF, 2+1 tabs (5h) | -5·1 (-9·4, -0·8) | -5·7 (-11·5, 0·2) | -0·6 (-7·8, 6·6) | 0·9 | -27·6 (-72·2,17·0) | -8·6 (-20·7, 3·4) | 19·0 (-22·5, 60·5) | 0·3 |
| F/TDF, 2+1 tabs (21h) | -4·8 (-8·7, -1·0) | -0·3 (-4·3, 3·7) | 4·5 (-1·5, 10·6) | 0·1 | -12·9 (-19·7, -6·1) | -9·6 (-16·6, -2·5) | 3·3 (-7·3, 13·9) | 0·5 |
| F/TAF, 2 tabs (5h) | -4·8 (-9·9, 0·3) | -4·5 (-13·3, 4·4) | 0·3 (-6·5, 7·2) | 0·9 | -20·6 (-50·2, 9·0) | -6·5 (-16·1, 3·1) | 14·1 (-12·9, 41·1) | 0·3 |
| F/TAF, 2 tabs (21h) | -13·0 (-19·5, -6·5) | -6·5 (-9·5, -3·4) | 6·6 (-0·8, 13·9) | 0·08 | 0·2 (-13·8, 14·3) | -8·3 (-20·8, 4·3) | -8·5 (-20·0, 2·9) | 0·1 |
| F/TAF, 2+1 tabs (5h) | -9·0 (-19·1, 1·1) | -7·9 (-14·9, -0·9) | 1·1 (-7·3, 9·5) | 0·8 | -13·9 (-22·2, -5·5) | -21·5 (-42·5, -0·5) | -7·6 (-27·8, 12·6) | 0·4 |
| F/TAF, 2+1 tabs (21h) | -10·5 (-16·8, -4·3) | -3·6 (-8·9, 1·6) | 6·9 (-1·6, 15·4) | 0·1 | -17·0 (-27·7, -6·3) | -2·3 (-14·9, 10·3) | 14·7 (-1·6, 31·4) | 0·08 |
| **PBMCs, p24 at day 15** | | | | | | | | |
| All trial arms combined | 2532 (1991, 3219) | 1241 (1016, 1515) | 0·58 (0·50, 0·67) | <0·0001 | 55·6 (41·9, 73·8) | 26·6 (22·9, 31·0) | 0·73 (0·61, 0·88) | 0·0009 |
| F/TDF, 2 tabs (5h) | 2822 (1758, 4532) | 2023 (1303, 3142) | 0·72 (0·43, 1·19) | 0·2 | 36·1 (20·9, 62·3) | 28·8 (18·4, 45·0) | 0·80 (0·56, 1·13) | 0·2 |
| F/TDF, 2 tabs (21h) | 2380 (1133, 4996) | 1454 (834, 2535) | 0·61 (0·39, 0·96) | 0·03 | 35·1 (16·3, 75·6) | 23·7 (14·1, 40·0) | 0·68 (0·35 1·29) | 0·2 |
| F/TDF, 2+1 tabs (5h) | 2267 (998, 5150) | 1065 (521, 2179) | 0·47 (0·33, 0·68) | 0·0008 | 43·4 (18·1, 104) | 19·7 (14·2, 27·2) | 0·45 (0·22, 0·93) | 0·03 |
| F/TDF, 2+1 tabs (21h) | 2521 (1084, 5862) | 1397 (724, 2697) | 0·55 (0·39, 0·79) | 0·003 | 34·5 (20·2, 58·8) | 34·5 (21·1, 56·5) | 1·00 (0·83, 1·21) | 1·0 |
| F/TAF, 2 tabs (5h) | 2334 (1168, 4667) | 1369 (772, 2426) | 0·59 (0·38, 0·90) | 0·02 | 35·2 (18·3, 67·6) | 25·8 (16·3, 40·9) | 0·73 (0·45, 1·19) | 0·2 |
| F/TAF, 2 tabs (21h) | 886 (285, 2759) | 654 (294, 1459) | 0·67 (0·45, 0·99) | 0·05 | 37·2 (17·1, 81·2) | 30·3 (16·8, 54·6) | 0·80 (0·50, 1·28) | 0·3 |
| F/TAF, 2+1 tabs (5h) | 3027 (1594, 5747) | 1293 (775, 2157) | 0·43 (0·25, 0·73) | 0·005 | 44·2 (23·1, 84·7) | 32·9 (19·8, 54·6) | 0·74 (0·31, 1·80) | 0·5 |
| F/TAF, 2+1 tabs (21h) | 1734 (840, 3580) | 1119 (667, 1879) | 0·65 (0·44, 0·94) | 0·03 | 28·5 (16·7, 48·9) | 20·9 (16·5, 26·4) | 0·73 (0·45, 1·18) | 0·2 |
| **PBMCs, area under the curve** | | | | | | | | |
| All trial arms combined | 46395 (39659, 54285) | 36911 (31336, 43479) | 0·87 (0·82, 0·92) | <0·0001 | 2236 (1943, 2473) | 1660 (1536, 1794) | 0·92 (0·86, 0·98) | 0·01 |
| F/TDF, 2 tabs (5h) | 53376 (43394, 65652) | 54286 (38649, 76250) | 1·02 (0·76, 1·36) | 0·9 | 1964 (1381, 2792) | 1797 (1310, 2464) | 0·92 (0·71, 1·19) | 0·5 |
| F/TDF, 2 tabs (21h) | 54782 (3373, 88859) | 44309 (28329, 69303) | 0·81 (0·69, 0·95) | 0·01 | 1763 (1211, 2566) | 1668 (1212, 2294) | 0·95 (0·82, 1·10) | 0·4 |
| F/TDF, 2+1 tabs (5h) | 45229 (25124, 81420) | 40536 (22637, 72588) | 0·90 (0·79, 1·02) | 0·08 | 1774 (1393, 2261) | 1462 (1278, 1673) | 0·82 (0·66, 1·03) | 0·08 |
| F/TDF, 2+1 tabs (21h) | 40366 (23242, 70107) | 35786 (20788, 61605) | 0·89 (0·82, 0·96) | 0·007 | 1856 (1516, 2272) | 1873 (1505, 2331) | 1·01 (0·95, 1·07) | 0·8 |
| F/TAF, 2 tabs (5h) | 42702 (26736, 68204) | 38715 (24213, 61903) | 0·91 (0·82, 1·01) | 0·07 | 1866 (1369, 2543) | 1736 (1361, 2213) | 0·93 (0·78, 1·11) | 0·4 |
| F/TAF, 2 tabs (21h) | 23788 (12479, 45345) | 21367 (12025, 37967) | 0·90 (0·78, 1·03) | 0·1 | 1669 (1249, 2230) | 1618 (1293, 2024) | 0·97 (0·82, 1·15) | 0·7 |
| F/TAF, 2+1 tabs (5h) | 48819 (29107, 81879) | 35595 (21700, 58388) | 0·73 (0·57, 0·93) | 0·01 | 1954 (1411, 2705) | 1678 (1343, 2096) | 0·86 (0·66, 1·12) | 0·2 |
| F/TAF, 2+1 tabs (21h) | 39961 (23444, 68115) | 34614 (21464, 55823) | 0·87 (0·78, 0·96) | 0·01 | 1605 (1327, 1941) | 1487 (1258, 1759) | 0·93 (0·74, 1·16) | 0·5 |
| **PBMCs, p24 slope** | | | | | | | | |
| All trial arms combined | -1·8 (-9·0, 6·2) | -6·0 (-8·1, -4·0) | -3·4 (-12·2, 5·4) | 0·4 | -2·7 (-5·3, -0·2) | -4·8 (-5·8, -3·7) | -1·2 (-3·6, 1·2) | 0·3 |
| F/TDF, 2 tabs (5h) | -1·5 (-115·1, 12·0) | -5·4 (-14·6, 3·8) | -3·9 (-24·0, 16·2) | 0·7 | -5·4 (-8·8, -2·1) | -4·7 (-7·2, -2·2) | 0·7 (-3·6, 5·1) | 0·7 |
| F/TDF, 2 tabs (21h) | 6·5 (-19·3, 32·3) | -2·5 (-9·1, 4·0) | -9·0 (-35·6, 17·7) | 0·5 | 1·2 (-9·9, 12·3) | -6·5 (-10·1, -3·0) | -7·7 (-18·0, 2·5) | 0·1 |
| F/TDF, 2+1 tabs (5h) | -7·3 (-20·9, 6·2) | -4·6 (-8·5, -0·7) | 2·7 (-11·0, 16·4) | 0·7 | -4·2 (-15·6, 7·2) | -3·2 (-3·8, -2·5) | 1·0 (-10·9, 12·9) | 0·9 |
| F/TDF, 2+1 tabs (21h) | -12·5 (-20·1, -4·9) | -9·2 (-16·9, -1·5) | 3·3 (-5·0, 11·6) | 0·4 | -6·8 (-10·9, -2·7) | -6·1 (-9·2, -3·0) | 0·8 (-1·6, 3·1) | 0·5 |
| F/TAF, 2 tabs (5h) | -14·1 (-21·8, -6·4) | -6·4 (-11·8, -1·1) | 7·7 (-1·7, 17·0) | 0·1 | -6·7 (-10·0, -3·3) | -3·6 (-4·4, -2·8) | 3·0 (-0·1, 6·2) | 0·06 |
| F/TAF, 2 tabs (21h) | 21·0 (-40·1, 82·0) | -5·4 (-8·7, -2·1) | -26·4 (-87·1, 34·4) | 0·4 | -1·3 (-10·1, 7·4) | -2·7 (-6·2, 0·8) | -1·4 (-9·8, 7·1) | 0·7 |
| F/TAF, 2+1 tabs (5h) | -2·5 (-16·4, 11·4) | -5·4 (-10·3, -0·4) | -2·8 (-17·9, 12·2) | 0·7 | -2·1 (-8·1, 4·0) | -7·9 (-14·1, -1·7) | -5·8 (-14·4, 2·8) | 0·2 |
| F/TAF, 2+1 tabs (21h) | -11·3 (-18·3, -4·2) | -9·1 (-16·5, -1·7) | 2·2 (-11·0, 15·3) | 0·7 | -3·7 (-7·5, 0·1) | -3·7 (-4·4, -3·0) | 0·0 (-3·4, 3·4) | 1·0 |

^a^*Ex* *vivo* dosing was performed with non-formulated TFV-FTC for samples from participants dosed with F/TDF, and with non-formulated TAF-FTC for those dosed with F/TAF.

^1^For slope outcomes, figures shown are arithmetic means; ^2^For slope outcomes, figures shown are arithmetic mean difference.

**Supplementary Table 6. Levels of TFV-DP and FTC-TP in tissue and PBMCs: comparisons between PrEP trial arms.**

|  |  | **Outcome = TFV-DP** | | | | **Outcome = FTC-TP** | | | |
| --- | --- | --- | --- | --- | --- | --- | --- | --- | --- |
| **Reference group** | **Comparator group** | **GM (95% CI), ref group** | **GM (95% CI), comparator** | **GMR (95% CI)** | **p-value** | **GM (95% CI), ref group** | **GM (95% CI), comparator** | **GMR (95% CI)** | **p-value** |
| **Compartment: tissue, units: pmol/gram for TFV-DP and FTC-TP** | | | | | | | | | |
| Overall effects of drug, dosage and interval, adjusted for each other | | | | | | | | | |
| F/TDF (all) | F/TAF (all) | 18·5 (14·9, 23·4) | 36·6 (28·7, 46·6) | 1·95 (1·41, 2·68) | 0·0001 | 326 (262, 405) | 273 (200, 374) | 0·85 (0·59, 1·22) | 0·4 |
| 2 tabs (both drugs) | 2+1 tabs (both drugs) | 21·7 (17·4, 27·2) | 31·7 (24·3, 41·3) | 1·45 (1·06, 2·00) | 0·02 | 339 (271, 424) | 262 (192, 356) | 0·77 (0·54, 1·11) | 0·2 |
| 5 hours (all regimens) | 21 hours (all regimens) | 22·3 (17·0, 29·2) | 30·8 (24·7, 38·4) | 1·38 (1·00, 1·90) | 0·05 | 401 (307, 524) | 221 (172, 284) | 0·55 (0·38, 0·79) | 0·001 |
| Effects of dosage, separately for each drug | | | | | | | | | |
| F/TDF, 2 tabs | F/TDF, 2 + 1 tabs | 14·8 (10·9, 20·1) | 23·8 (17·1, 33·1) | 1·61 (1·03, 2·50) | 0·04 | 362 (260, 504) | 292 (217, 392) | 0·81 (0·52, 1·25) | 0·3 |
| F/TAF, 2 tabs | F/TAF, 2 +1 tabs | 32·0 (24·1, 42·3) | 41·8 (28·9, 62·9) | 1·31 (0·81, 2·13) | 0·3 | 317 (230, 437) | 235 (136, 409) | 0·74 (0·40, 1·39) | 0·3 |
| Effects of interval, separately for each drug and dosage | | | | | | | | | |
| F/TDF, 2 tabs (5h) | F/TDF, 2 tabs (21h) | 10·7 (6·2, 18·4) | 20·4 (15·7, 26·5) | 1·90 (1·07, 3·40) | 0·03 | 508 (307, 841) | 257 (170, 390) | 0·51 (0·27, 0·95) | 0·03 |
| F/TDF, 2+1 tabs (5h) | F/TDF, 2+1 tabs (21h) | 22·1 (13·7, 35·7) | 25·6 (15·4, 42·8) | 1·16 (0·59, 2·26) | 0·7 | 411 (300, 565) | 203 (126, 326) | 0·49 (0·29, 0·84) | 0·01 |
| F/TAF, 2 tabs (5h) | F/TAF, 2 tabs (21h) | 26·1 (16·7, 40·8) | 39·2 (27·4, 56·1) | 1·50 (0·87, 2·60) | 0·1 | 392 (239, 642) | 257 (165, 399) | 0·65 (0·35, 1·23) | 0·2 |
| F/TAF, 2+1 tabs (5h) | F/TAF, 2+1 tabs (21h) | 40·1 (21·4, 75·1) | 43·7 (24·1, 79·1) | 1·09 (0·48, 2·49) | 0·8 | 315 (133, 745) | 176 (82, 378) | 0·56 (0·19, 1·69) | 0·3 |
| **Compartment: PBMCs, units: fmol/10^6^ cells for TFV-DP, pmol/10^6^ cells for FTC-TP^1^** | | | | | | | | | |
| Overall effects of drug, dosage and interval, adjusted for each other | | | | | | | | | |
| F/TDF (all) | F/TAF (all) | 6·7 (5·1, 8·9) | 50·0 (36·9, 67·6) | 7·47 (4·96, 11·2) | <0·0001 | 2·4 (1·7, 3·2) | 2·4 (1·8, 3·2) | 1·01 (0·67, 1·54) | 0·9 |
| 2 tabs (both drugs) | 2+1 tabs (both drugs) | 16·5 (11·5, 23·8) | 22·6 (14·7, 34·6) | 1·42 (0·94, 2·14) | 0·09 | 2·4 (1·8, 3·3) | 2·3 (1·7, 3·0) | 0·95 (0·62, 1·43) | 0·8 |
| 5 hours (all regimens) | 21 hours (all regimens) | 19·4 (12·8, 29·3) | 19·4 (13·1, 28·6) | 0·95 (0·63, 1·42) | 0·8 | 2·9 (2·1, 4·0) | 1·9 (1·5, 2·5) | 0·65 (0·43, 0·99) | 0·04 |
| Effects of dosage, separately for each drug | | | | | | | | | |
| F/TDF, 2 tabs | F/TDF, 2 + 1 tabs | 6·9 (4·6, 10·2) | 6·6 (4·4, 10·0) | 0·96 (0·55, 1·69) | 0·9 | 2·9 (1·9, 4·4) | 1·9 (1·2, 3·0) | 0·67 (0·37, 1·22) | 0·2 |
| F/TAF, 2 tabs | F/TAF, 2 +1 tabs | 35·3 (22·8, 54·5) | 70·8 (47·0, 106) | 2·01 (1·12, 3·60) | 0·02 | 2·1 (1·3, 3·3) | 2·7 (1·8, 4·0) | 1·28 (0·71, 2·32) | 0·4 |
| Effects of interval, separately for each drug and dosage | | | | | | | | | |
| F/TDF, 2 tabs (5h) | F/TDF, 2 tabs (21h) | 5·9 (3·3, 10·6) | 8·3 (4·4, 15·5) | 1·40 (0·62, 3·15) | 0·4 | 2·9 (1·4, 6·0) | 2·8 (1·6, 4·9) | 0·98 (0·41, 2·36) | 1·0 |
| F/TDF, 2+1 tabs (5h) | F/TDF, 2+1 tabs (21h) | 7·2 (3·6, 14·6) | 6·2 (3·5, 10·9) | 0·85 (0·36, 1·99) | 0·7 | 2·3 (1·1, 4·8) | 1·6 (0·9, 3·0) | 0·71 (0·29, 1·72) | 0·4 |
| F/TAF, 2 tabs (5h) | F/TAF, 2 tabs (21h) | 36·0 (19·5, 66·5) | 34·6 (17·2, 69·4) | 0·96 (0·39, 2·34) | 0·9 | 2·7 (1·4, 5·3) | 1·6 (0·8, 3·2) | 0·61 (0·24, 1·53) | 0·3 |
| F/TAF, 2+1 tabs (5h) | F/TAF, 2+1 tabs (21h) | 79·8 (42·6, 149) | 63·3 (34·7, 115) | 0·79 (0·35, 1·82) | 0·6 | 4·1 (2·2, 7·6) | 1·8 (1·1, 3·0) | 0·45 (0·21, 0·95) | 0·04 |

^1^15 participants had undetectable TFV-DP in PBMCs [2 in F/TDF 2 tabs (5h), 4 in F/TDF 2 tabs (21h), 3 in F/TDF 2+1 tabs (5h), 1 in F/TDF 2+1 tabs (21h), 2 in F/TAF 2 tabs (5h), 1 in F/TAF 2 tabs (21h), 1 in F/TAF 2+1 tabs (5h), 1 in F/TAF 2+1 tabs (21h)]; 9 participants had undetectable FTC-TP in PBMCs [1 in F/TDF 2 tabs (5h), 1 in F/TDF 2 tabs (21h), 2 in F/TDF 2+1 tabs (5h), 1 in F/TDF 2+1 tabs (21h), 2 in F/TAF 2 tabs (5h), 1 in F/TAF 2 tabs (21h), 1 in F/TAF 2+1 tabs (5h), 0 in F/TAF 2+1 tabs (21h)]

**Supplementary Table 7. Levels of TFV and FTC in plasma and tissue: comparisons between PrEP trial arms.**

|  |  | **Outcome = TFV** | | | | **Outcome = FTC** | | | |
| --- | --- | --- | --- | --- | --- | --- | --- | --- | --- |
| **Reference group** | **Comparator group** | **GM (95% CI), ref group** | **GM (95% CI), comparator** | **GMR (95% CI)** | **p-value** | **GM (95% CI), ref group** | **GM (95% CI), comparator** | **GMR (95% CI)** | **p-value** |
| **Compartment: plasma, units: ng/mL for TFV and FTC** | | | | | | | | | |
| Overall effects of drug, dosage and interval, adjusted for each other | | | | | | | | | |
| F/TDF (all) | F/TAF (all) | 74·9 (64·8, 86·4) | 4·8 (4·2, 5·4) | 0·06 (0·05, 0·07) | <0·0001 | 375 (263, 534) | 347 (246, 489) | 0·91 (0·81, 1·03) | 0·1 |
| 2 tabs (both drugs) | 2+1 tabs (both drugs) | 19·5 (13·2, 28·9) | 17·2 (12·0, 24·6) | 0·95 (0·81, 1·10) | 0·5 | 435 (302, 627) | 295 (214, 407) | 0·70 (0·62, 0·78) | <0·0001 |
| 5 hours (all regimens) | 21 hours (all regimens) | 25·1 (17·0, 37·3) | 13·4 (9·5, 18·8) | 0·52 (0·45, 0·61) | <0·0001 | 1455 (1209, 1519) | 97·7 (90·6, 105) | 0·07 (0·06, 0·08) | <0·0001 |
| Effects of dosage, separately for each drug | | | | | | | | | |
| F/TDF, 2 tabs | F/TDF, 2 + 1 tabs | 81·1 (64·7, 102) | 68·6 (57·2, 82·1) | 0·85 (0·63, 1·13) | 0·3 | 453 (262, 784) | 304 (192, 480) | 0·69 (0·35, 1·37) | 0·3 |
| F/TAF, 2 tabs | F/TAF, 2 +1 tabs | 4·7 (3·8, 5·8) | 4·9 (4·2, 5·7) | 1·04 (0·80, 1·36) | 0·8 | 417 (250, 698) | 288 (179, 462) | 0·67 (0·33, 1·36) | 0·3 |
| Effects of interval, separately for each drug and dosage | | | | | | | | | |
| F/TDF, 2 tabs (5h) | F/TDF, 2 tabs (21h) | 136 (109, 170) | 48·3 (42·3, 55·2) | 0·36 (0·28, 0·46) | <0·0001 | 1947 (1592, 2383) | 105 (89·2, 125) | 0·05 (0·04, 0·07) | <0·0001 |
| F/TDF, 2+1 tabs (5h) | F/TDF, 2+1 tabs (21h) | 94·6 (73·1, 122) | 50·8 (44·1, 58·5) | 0·54 (0·41, 0·71) | 0·0001 | 993 (820, 1202) | 101 (86·0, 118) | 0·10 (0·08, 0·13) | <0·0001 |
| F/TAF, 2 tabs (5h) | F/TAF, 2 tabs (21h) | 6·4 (5·2, 7·9) | 3·4 (2·5, 4·8) | 0·53 (0·37, 0·77) | 0·002 | 1663 (1391, 1988) | 105 (96·7, 114) | 0·06 (0·05, 0·08) | <0·0001 |
| F/TAF, 2+1 tabs (5h) | F/TAF, 2+1 tabs (21h) | 5·7 (4·3, 7·6) | 4·2 (3·7, 4·7) | 0·73 (0·54, 0·98) | 0·04 | 1008 (853, 1190) | 82·2 (67·7, 99·8) | 0·08 (0·06, 0·10) | <0·0001 |
| **Compartment: tissue, units: ng/g for TFV and FTC^1^** | | | | | | | | | |
| Overall effects of drug, dosage and interval, adjusted for each other | | | | | | | | | |
| F/TDF (all) | F/TAF (all) | 38·3 (23·6, 62·2) | 25·4 (17·8, 36·2) | 0·58 (0·29, 1·14) | 0·1 | 218 (168, 281) | 191 (145, 251) | 0·89 (0·62, 1·29) | 0·5 |
| 2 tabs (both drugs) | 2+1 tabs (both drugs) | 38·3 (20·0, 73·1) | 28·2 (22·4, 35·4) | 0·64 (0·33, 1·24) | 0·2 | 228 (176, 297) | 180 (138, 234) | 0·79 (0·55, 1·15) | 0·2 |
| 5 hours (all regimens) | 21 hours (all regimens) | 29·9 (22·7, 39·3) | 36·1 (19·0, 68·4) | 1·26 (0·67, 2·39) | 0·4 | 217 (172, 275) | 176 (130, 239) | 0·81 (0·55, 1·20) | 0·3 |

^1^104 participants had undetectable TFV in tissue [13 in F/TDF 2 tabs (5h), 13 in F/TDF 2 tabs (21h), 11 in F/TDF 2+1 tabs (5h), 12 in F/TDF 2+1 tabs (21h), 13 in F/TAF 2 tabs (5h), 13 in F/TAF 2 tabs (21h), 15 in F/TAF 2+1 tabs (5h), 14 in F/TAF 2+1 tabs (21h)]; 49 participants had undetectable FTC in tissue [2 in F/TDF 2 tabs (5h), 9 in F/TDF 2 tabs (21h), 4 in F/TDF 2+1 tabs (5h), 11 in F/TDF 2+1 tabs (21h), 2 in F/TAF 2 tabs (5h), 10 in F/TAF 2 tabs (21h), 2 in F/TAF 2+1 tabs (5h), 9 in F/TAF 2+1 tabs (21h)].

**Supplementary Table 8. Listing of TFV-DP and FTC-TP levels by compartment in foreskin CD4 sub-study participants.**

|  | TFV-DP | | | | FTC-TP | | | | p24 inhibition at day 15 compared to control |
| --- | --- | --- | --- | --- | --- | --- | --- | --- | --- |
| Study ID | CD4+ (fmol/  10^6^ cells) | CD4- (fmol/  10^6^ cells) | PBMC (fmol/  10^6^ cells) | Tissue (pmol/ gram) | CD4+ (pmol/  10^6^ cells) | CD4- (pmol/  10^6^ cells) | PBMC (pmol/  10^6^ cells) | Tissue (pmol/ gram) |  |
| CJ002 | ND | ND | 391·6 | 18·37 | ND | ND | 24·66 | 122·45 | 84% |
| CJ007 | ND | 4·11 | ND | 13·80 | ND | 0·17 | 3·57 | 53·01 | 89% |
| CJ011 | ND | 3·05 | 118·22 | 11·40 | ND | 0·13 | 1·55 | 51·64 | 92% |
| CJ013 | ND | 3·44 | 38·64 | 27·62 | ND | 0·08 | 6·54 | 149·71 | 87% |
| CJ016 | ND | ND | 201·18 | 7·52 | ND | ND | 9·03 | 54·30 | 94% |
| CJ021 | ND | 5·75 | 17·95 | 1·79 | ND | 0·16 | 0·61 | 1·79 | 87% |
| CJ027 | ND | 27·34 | 15·43 | 25·51 | ND | 0·83 | 1·22 | 168·37 | 88% |
| CJ029 | 4·57 | ND | 14·85 | 48·83 | 0·08 | ND | 0·41 | 248·22 | 90% |
| CJ032 | ND | 7·94 | 1·29 | 25·13 | ND | 0·03 | 0·32 | 141·54 | 96% |
| CJ051 | ND | 5·30 | 2·4 | 26·92 | ND | 0·11 | 0·44 | 489·74 | 99% |
| CJ054 | ND | 3·69 | 12·2 | 9·27 | ND | 0·03 | 2·71 | 109·74 | 87% |
| CJ063 | 16·79 | ND | 4·77 | 17·70 | 0·44 | ND | 2·52 | 296·46 | 99% |
| CJ067 | 6·02 | ND | 1·24 | No sample | 0·09 | ND | 0·34 | No sample | 87% |
| CJ072 | 3·10 | ND | 16·17 | 43·14 | 0·15 | ND | 0·87 | 232·02 | 97% |
| CJ073 | 13·27 | ND | 1·57 | 38·82 | 0·21 | ND | 0·34 | 239·13 | 97% |
| CJ079 | 10·42 | ND | 2·91 | 26·37 | 0·34 | ND | 1·06 | 1028·4 | 97% |
| EB040 | ND | 28·78 | 20·2 | 49·16 | ND | 0·30 | 7·43 | 234·55 | 90% |
| EB042 | ND | 12·95 | 105·08 | 122·08 | ND | 0·51 | 3·19 | 425·49 | 90% |
| EB045 | ND | 34·38 | 13·73 | 63·15 | 1·28 | 0·78 | 3·06 | 261·91 | 92% |
| EB051 | 71·59 | 63·25 | 178·14 | 35·64 | 4·43 | 2·21 | 3·17 | 209·90 | 97% |
| EB052 | ND | 15·33 | 77·06 | 88·94 | 2·53 | 1·06 | 5·43 | 1025·24 | 97% |
| EB056 | ND | 33·22 | 17·49 | 83·83 | 2·91 | 0·84 | 5·20 | 922·08 | 96% |
| EB060 | ND | 20·49 | 11·7 | 44·58 | ND | 0·40 | 2·27 | 440·96 | 88% |
| EB061 | 148·94 | 49·55 | 324·64 | 195·82 | 3·30 | 0·84 | 6·93 | 1443·00 | 83% |
| EB065 | ND | 30·40 | 11·3 | 26·57 | ND | 0·57 | 1·81 | 181·12 | 90% |
| EB068 | ND | 19·86 | 266·67 | 99·22 | 2·41 | 0·49 | 6·48 | 521·74 | 84% |
| EB071 | 504·07 | 6·15 | 141·78 | 83·51 | 13·40 | 0·36 | 9·37 | 967·88 | 78% |
| EB075 | 80·00 | 39·27 | 184·62 | 91·43 | 4·32 | 0·96 | 4·72 | 380·00 | 90% |
| Summary statistics, overall and by trial arm; comparisons by drug | | | | | | | | | |
| GM (95% CI), overall | 24·7  (7·5, 81·9) | 13·8  (8·7, 22·0) | 24·9  (12·3, 50·4) | 33·2  (22·3, 49·3) | 0·97  (0·38, 2·52) | 0·32  (0·19, 0·57) | 2·30  (1·46, 3·63) | 224·4  (132·8, 379·3) | - |
| GM (95% CI), F/TDF trial arms | 10·9  (5·4, 21·9) | 13·0  (6·1, 27·8) | 6·3  (3·1, 12·7) | 31·5  (21·7, 45·8) | 0·47  (0·13, 1·74) | 0·19  (0·07, 0·53) | 1·58  (0·79, 3·17) | 291·4  (185·2, 458·6) | - |
| GM (95% CI), F/TAF trial arms | 42·7  (5·2, 352·0) | 14·5  (7·2, 29·1) | 89·2  (44·5, 178·8) | 34·6  (17·2, 69·4) | 1·69  (0·39, 7·35) | 0·50  (0·27, 0·92) | 3·19  (1·70, 5·98) | 182·1  (72·5, 457·3) | - |
| GMR (95% CI), F/TAF *versus* F/TDF | 3·93  (0·36, 43·26)  p=0·22 | 1·11  (0·43, 2·90)  p=0·82 | 14·13  (5·53, 36·13)  p<0·0001 | 1·10  (0·49, 2·47)  P=0·82 | 3·60  (0·57, 22·77) p=0·16 | 2·60  (0·91, 7·42)  p=0·07 | 2·02  (0·83, 4·93)  p=0·12 | 0·62  (0·22, 1·81)  p=0·37 | - |
| % detectable | 36 | 71 | 96 | 100 | 50 | 71 | 100 | 100 | - |

ND = not detectable

**Supplementary Table 9. Correlations between TFV-DP and FTC-TP levels in PBMCs, CD4+ cells, CD4- cells and tissue, among participants in the CD4 sub-study.**

| **Parameter** | **TFV-DP in PBMCs** | **TFV-DP in CD4+ cells** | **TFV-DP in CD4- cells** | **TFV-DP in foreskin tissue** |
| --- | --- | --- | --- | --- |
| TFV-DP in PBMCs | 1 |  |  |  |
| TFV-DP in CD4+ cells | 0·06 (r=0·25, p=0·2, n=26) | 1 |  |  |
| TFV-DP in CD4- cells | 0·57 (r=0·76, p=0·01, n=10) | 0·35 (r=0·59, p=0·09, n=9) | 1 |  |
| TFV-DP in foreskin tissue | 0·07 (r=0·27, p=0·3, n=19) | 0·35 (r=0·59, p=0·006, n=20) | 0·86 (r=-0·93, p=0·07, n=4) | 1 |
| **Parameter** | **FTC-TP in PBMCs** | **FTC-TP in CD4+ cells** | **FTC-TP in CD4- cells** | **FTC-TP in foreskin tissue** |
| FTC-TP in PBMCs | 1 |  |  |  |
| FTC-TP in CD4+ cells | 0·05 (r=0·23, p=0·3, n=27) | 1 |  |  |
| FTC-TP in CD4- cells | 0·87 (r=0·93, p<0·001, n=14) | 0·25 (r=0·50, p=0·08, n=13) | 1 |  |
| FTC-TP in foreskin tissue | 0·22 (r=0·47, p=0·04, n=20) | 0·18 (r=0·43, p=0·06, n=20) | 0·07 (r=-0·26, p=0·5, n=8) | 1 |

Figures shown are r^2^ between log-transformed values with corresponding Pearson’s correlation coefficient, p-value and sample size in parentheses.

**Supplementary Table 10. Effect of *ex vivo* dosing (PEP) at specific time points on p24 at day 15 in tissue, following *ex vivo* challenge in control arm participants.**

|  | **High titre challenge** | | | | **Low titre challenge** | | | |
| --- | --- | --- | --- | --- | --- | --- | --- | --- |
| **Time of dosing** | **TFV-FTC,**  **GMR (95% CI)** | **p-value** | **TAF-FTC,**  **GMR (95% CI)** | **p-value** | **TFV-FTC,**  **GMR (95% CI)** | **p-value** | **TAF-FTC,**  **GMR (95% CI)** | **p-value** |
| Dosed 1h after *ex vivo* challenge | 0·07 (0·05, 0·11) | <0·0001 | 0·06 (0·04, 0·09) | <0·0001 | 0·04 (0·01, 0·13) | <0·0001 | 0·03 (0·01, 0·09) | <0·0001 |
| Dosed 24h after *ex vivo* challenge | 0·08 (0·06, 0·11) | <0·0001 | 0·06 (0·04, 0·09) | <0·0001 | 0·05 (0·02, 0·12) | <0·0001 | 0·05 (0·02, 0·10) | <0·0001 |
| Dosed 48h after *ex vivo* challenge | 0·24 (0·17, 0·34) | <0·0001 | 0·17 (0·11, 0·26) | <0·0001 | 0·27 (0·13, 0·55) | 0·001 | 0·11 (0·03, 0·34) | 0·0008 |
| Dosed 72h after *ex vivo* challenge | 0·50 (0·41, 0·62) | <0·0001 | 0·39 (0·32, 0·48) | <0·0001 | 0·62 (0·49, 0·77) | 0·0003 | 0·41 (0·29, 0·58) | 0·0001 |

Figures shown are GMR (95% CI) comparing p24 levels in control group samples that received PEP *versus* control group samples who received no PEP.

**Supplementary Table 11. Comparison of *ex vivo* dosing at specific time points with TAF-FTC *versus* TFV-FTC on p24 at day 15 in tissue, following *ex vivo* challenge in control arm participants.**

|  | **High titre challenge** | | | | **Low titre challenge** | | | |
| --- | --- | --- | --- | --- | --- | --- | --- | --- |
| **Time of dosing** | **TFV-FTC,**  **GM (95% CI)** | **TAF-FTC,**  **GM (95% CI)** | **GMR**  **(95% CI)** | **p-value** | **TFV-FTC,**  **GM (95% CI)** | **TAF-FTC,**  **GM (95% CI)** | **GMR**  **(95% CI)** | **p-value** |
| Dosed 1h after *ex vivo* challenge | 27 (41-62) | 32 (20-51) | 1·29 (1·06-1·57) | 0·02 | 1·8 (0·6-5·9) | 1·1 (0·3-4·5) | 0·64 (0·34-1·21) | 0·2 |
| Dosed 24h after *ex vivo* challenge | 45 (33-62) | 35 (24-49) | 0·77 (0·59-1·00) | 0·05 | 2·1 (0·7-5·7) | 2·1 (0·9-4·9) | 1·00 (0·66-1·53) | 1·0 |
| Dosed 48h after *ex vivo* challenge | 136 (96-191) | 96 (64-143) | 0·70 (0·56-0·89) | 0·005 | 11·8 (4·7-30·1) | 4·8 (1·2-18·9) | 0·40 (0·17-0·97) | 0·04 |
| Dosed 72h after *ex vivo* challenge | 282 (235-338) | 219 (179-269) | 0·78 (0·69-0·87) | 0·0003 | 27·5 (17·5-43·0) | 18·1 (9·9-33·2) | 0·66 (0·47-0·92) | 0·02 |

GMR (95% CI) for p24 at day 15 in control arm samples that did not receive PEP were 1560 (454-690) and 45 (30-67) for high and low titre challenge, respectively

**Supplementary Figure 1. Schematic of the randomisation groups and foreskin ex vivo challenge**

**
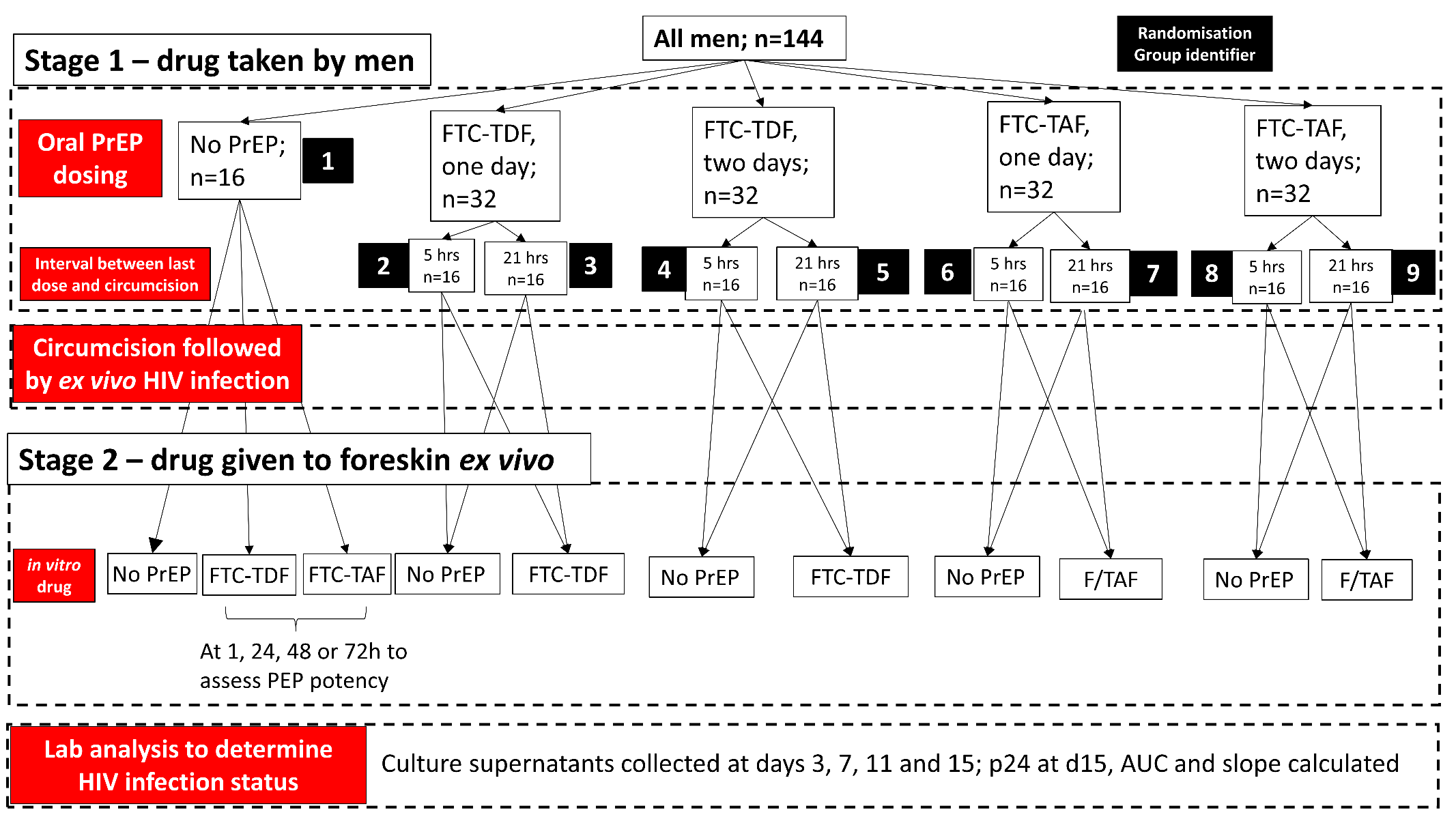
**


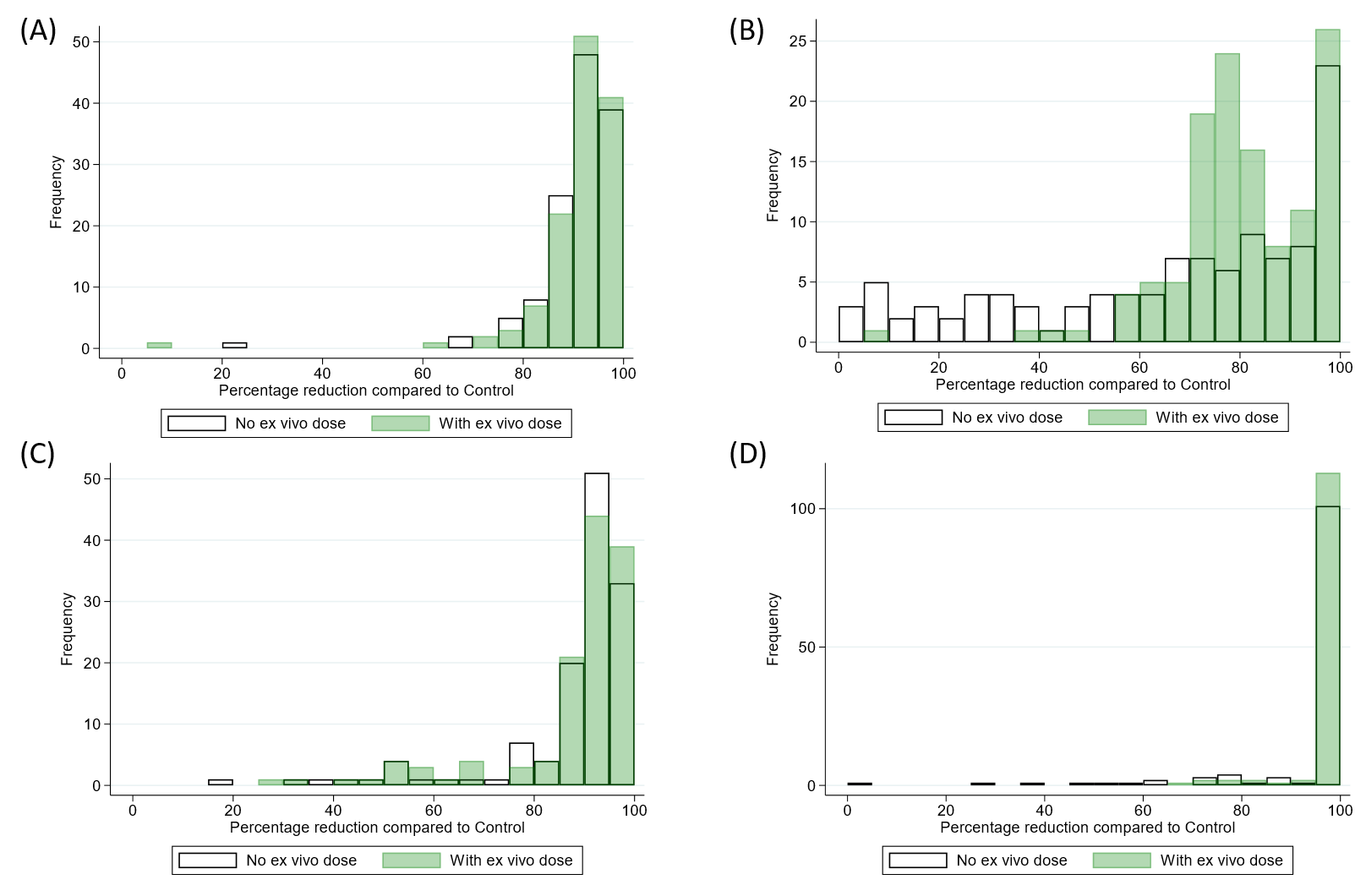
**Supplementary Figure 2. Percentage reduction in p24 at day 15 among participants receiving PrEP compared to control, with and without ex vivo dosing following: (A) high titre challenge in tissue, (B) high titre challenge in PBMCs, (C) low titre challenge in tissue, (D) low titre challenge in PBMCs.** Clear bars show percentage reduction for samples which did not receive ex vivo dosing, green bars show percentage reduction for samples which received ex vivo dosing.

APPENDIX: STUDY PROTOCOL

Combined HIV Adolescent PrEP and Prevention: On demand Pre-
exposure prophylaxis to provide protection from HIV in men - using

foreskin tissue to estimate protection (Phase II)

The CHAPS Trial

Trial Identifiers:

EudraCT Number - REC Number -

Version 1.2

Date 20 April 2021

Trial Administration

PRINCIPAL INVESTIGATOR

Dr Neil Martinson

Perinatal HIV Research Unit, A Division of the Wits Health Consortium (Pty) Ltd,

Chris Hani Baragwanath Academic Hospital, Soweto, South Africa, P.O Box 114, Diepkloof 1864 Tel: (011) 989 9836

Email: [CHAPS@phru.co.za](mailto:CHAPS@phru.co.za)

CO-ORDINATIING CENTRE

| Perinatal HIV Research Unit | |
| --- | --- |
| A Division of the Wits Health Consortium (Pty) Ltd, | New Nurses Home, West Wing |
| Chris Hani Baragwanath Academic Hospital, Soweto | , South Africa, P.O Box 114, Diepkloof |
| Tel: (011) 989 9836 [CHAPS@phru.co.za](mailto:CHAPS@phru.co.za) |  |
| Trial Physician South Africa: Dr Neil Martinson | [martinson@phru.co.za](mailto:martinson@phru.co.za) |
| Trial Physician Uganda: Professor Pontiano Kaleebu | [pontiano.kaleebu@mrcuganda.org](mailto:pontiano.kaleebu@mrcuganda.org) |
| Trial Statistician: Stephen Nash | [stephen.nash@lshtm.ac.uk](mailto:stephen.nash@lshtm.ac.uk) |
| Clinical Project Manager: Dr Limakatso Lebina | [lebinal@phru.co.za](mailto:lebinal@phru.co.za) |
| CHAPS lead: Dr Julie Fox | [julie.fox@kcl.ac.uk](mailto:julie.fox@kcl.ac.uk) |

Co-Investigators

| Professor Clive Gray | University of Cape Town, Cape Town, South Africa |
| --- | --- |
| Professor Saye Khoo | University of Liverpool, UK |
| Dr Carolina Herrera | Imperial College London, UK |
| Dr Jennifer Serwanga | MRC/UVRI & LSHTM Uganda Research Unit |
| Professor Francesca Chiodi | Karolinska Institutet, Stockholm, Sweden |
| Professor Janet Seeley | MRC/UVRI & LSHTM Uganda Research Unit |
| Professor Helen Weiss | LSHTM |
| Professor Pontiano Kaleebu | MRC/UVRI & LSHTM Uganda Research Unit |
| Dr Julie Fox | Kings College London, UK |


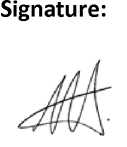


**Neil Martinson**

**Date: 20 April 2021**

**Principal Investigator**

1. Study Synopsis

| **Title of clinical trial** | Combined HIV Adolescent PrEP and Prevention: On demand Pre­exposure prophylaxis (PrEP) to provide protection from HIV in men  - using foreskin tissue to estimate protection |
| --- | --- |
| **Protocol Short Title** | CHAPS |
| **Study Phase** | Phase 2 |
| **Sponsor name** | Perinatal HIV Research Unit, A Division of the Wits Health  Consortium (Pty) Ltd, South Africa. |
| **Purpose of clinical trial** | Using foreskin tissue in an explant model, investigate the optimum drug,  dose and schedule for on-demand PrEP for insertive sex |
| **Objectives** | **Primary objective:**  To compare the effect of different PrEP drugs (FTC-TDF and FTC-TAF),  doses and timing of doses on p24 antigen level in resected foreskin tissue  following HIV exposure ex vivo challenge.  **Secondary objectives:**   1. Investigate the timing and dose of FTC-TDF and FTC-TAF as oral   PrEP and in vitro PEP (applied directly to removed foreskin tissue)  required to prevent ex vivo HIV infection in foreskin tissue and  blood using the explant model   1. Determine blood, rectal fluid and foreskin tissue concentrations of   FTC, TFV, TAF and their active metabolites that are required for ex  vivo HIV protection   1. Evaluate inflammation, cellular activation, foreskin mucosal   integrity, gene expression and microbiome in foreskin tissue  following oral in vivo PrEP and in vitro PEP   1. Evaluate the efficacy of in vitro post exposure dosing with PrEP in   protection against ex vivo HIV infection using the explant model   1. To investigate sexual behaviour, PrEP acceptability and feedback on   HIV prevention trials implementation |
| **Trial Design** | Open-label, randomised controlled trial (RCT) |
| **Primary endpoints** | Non-infection of foreskin tissue with HIV (in the lab) at 15 days following  e*x vivo* challenge. This is because we wish to demonstrate the potential  protective effect of the drugs |

| **Sample Size** | 144 evaluable individuals - 72 each in Uganda and South Africa |
| --- | --- |
| **Summary of eligibility criteria** | **Inclusion Criteria**  Participants must satisfy all the following criteria within 21 days  prior to their circumcision visit:  1. Clinically eligible for either forceps guided, or dorsal slit  circumcision  2. The ability to understand and sign a written informed  consent form by participant (**and** participant's legal guardian  if younger than 18 years) prior to participation in any study  procedures and to comply with all trial requirements  3. Male sex at birth  4. Age 13- 24 years  5. Haemoglobin >9g/dL  6. Weight >35Kg  7. Two negative rapid HIV antibody tests results (manufactured  by different companies), dating from 21 days or less prior to  VMMC  8. Two locator information details (including physical address,  telephone contacts, email) for contacting of either patient or  their parent  **Exclusion Criteria**  1. Any significant acute or chronic medical illness and current  therapy that in the opinion of the site investigator would  preclude receipt either of investigational products, or VMMC  2. Any evidence that participant is not suitable for VMMC |
| **IMP, dosage and route of** | Oral emtricitabine and tenofovir disoproxil fumarate (FTC-TDF) or oral |
| **administration** | Emtricitabine/Tenofovir Alafenamide (FTC-TAF): different daily dosing  schedules. Maximum study dose will be 3 tablets taken on two  consecutive days prior to VMMC |
| **Maximum duration of**  **treatment of a participant** | Two days |

1. Glossary of Terms

AE - adverse event

AIDS - acquired immunodeficiency syndrome

AR - adverse drug reaction

ARV - antiretroviral

AUC- area under the curve

BMI - body mass index

CAB - community advisory board

CRF - case report form

DOT- directly observed therapy

DSUR - development safety update report

eCRF - electronic case report form

EDTA - ethylenediaminetetraacetic acid

FDA - US food & drug administration

FTC-TAF - Emtricitabine Tenofovir Alafenamide

FTC - Emtricitabine

FTC-TP - Emtricitabine triphosphate

GCP - good clinical practice

HIV - Human Immunodeficiency Virus

IDMC- independent data monitoring committee

IMP - investigational medicinal product

KCL - King's College London

LSHTM - The London School of Hygiene & Tropical Medicine

MSM - men who have sex with men

PBMC - peripheral blood mononuclear cell

PD - pharmacodynamics

PEP - post exposure prophylaxis

PHRU - Perinatal HIV Research Unit

PK - pharmacokinetics

PrEP - pre-exposure prophylaxis

RCT - randomised controlled trial

REC - research ethics committee

RMPRU- Respiratory and Meningeal Pathogens Research Unit SAE - serious adverse event SAR - serious adverse reaction SAS - Statistical Analysis Software

SmPC - summary of product characteristics

SOP - standard operating procedure

STI - sexually transmitted infection

SUSAR - suspected unexpected serious adverse reaction TAF - Tenofovir Alafenamide

TDF - Tenofovir disoproxil fumarate

TFV - Tenofovir

TFV-DP - Tenofovir diphosphate

TMG - trial management group

UAR - unexpected adverse reaction

USAR - unexpected serious adverse reaction

VMMC - Voluntary male medical circumcision

WHO - World Health Organisation

Table of Contents

***1***[***.Study Synopsis***](#bookmark7)

1. [***Glossary of Terms***](#bookmark9)
2. [***Background & Rationale***](#bookmark11)
3. [***Trial Objectives and Design***](#bookmark13)
   1. [**Trial Objectives**](#bookmark14)
      1. [Primary endpoint](#bookmark19)
      2. [Secondary endpoints](#bookmark20)

[**4.2. Trial Design**](#bookmark21)

1. [***Trial Medication***](#bookmark27)
   1. [**Investigational Medicinal Product (IMP)**](#bookmark29)
   2. [**Dosing Regimen**](#bookmark31)
   3. [**IMP Risks**](#bookmark33)
   4. [**Drug Accountability**](#bookmark35)
   5. [**Storage of IMP**](#bookmark37)
   6. [**Dosing Schedule**](#bookmark39)
   7. [**Concomitant Medication**](#bookmark41)
2. [***Selection and Withdrawal of Participants***](#bookmark43)
   1. [**Inclusion Criteria**](#bookmark45)
   2. [**Exclusion Criteria**](#bookmark47)
   3. [**Selection of Participants**](#bookmark49)
   4. [**Randomisation Procedure**](#bookmark51)
   5. [**Withdrawal of Participants**](#bookmark53)
   6. [**Expected Duration of Trial**](#bookmark55)
3. ***Trial Procedures***

[**7.1 By Visit**](#bookmark57)

[7.1.1 Screening Visit](#bookmark59)

1. [Randomisation visit](#bookmark60)
2. [DOT PrEP dosing visits +/-1 hour of expected next dose](#bookmark61)
3. [VMMC sample collection visit](#bookmark62)
4. [Exit safety visit after VMMC](#bookmark63)
5. [**Laboratory Tests**](#bookmark65)
6. [**Clinical Research sample processing**](#bookmark70)

[Laboratory Research Sample analysis](#bookmark72)

1. [**End point Sample Analysis**](#bookmark80)
2. [***Assessment of Safety***](#bookmark89)

***3***

***5***

***8***

***10***

**10**

10

11

**11**

***23***

**23**

**23**

**23**

**24**

**25**

**25**

**25**

***26***

**26**

**26**

**26**

**27**

**27**

**28**

**28**

**28**

28

30

31

31

33

**33**

**34**

34

**35**

***39***

- 1. [Specification, Timing and Recording of Safety Parameters 39](#bookmark92)
  2. [Management of potential adverse outcomes after VMMC 39](#bookmark94)
  3. [Management of HIV-1 diagnosis 39](#bookmark96)
  4. [Procedures for Recording and Reporting Adverse Events 39](#bookmark98)
  5. [Treatment Stopping Rules 41](#bookmark102)

9. Statistics …………………………………..…..41

[9.1 Sample Size 41](#bookmark105)

[9.2 Analysis 41](#bookmark107)

1. [Criteria for termination of the trial 43](#bookmark109)
2. [Trial Management Group (TMG) 43](#bookmark111)
3. [Trial Steering Committee (TSC) 44](#bookmark113)
4. [Independent Data Monitoring Committee (IDMC) 44](#bookmark115)
5. [Direct Access to Source Data and Documents 44](#bookmark117)
6. [Ethics & Regulatory Approvals 45](#bookmark119)
7. [Quality Assurance 45](#bookmark121)
8. [Data Handling 46](#bookmark123)
9. [Data Management 46](#bookmark125)
10. [Publication Policy 47](#bookmark127)
11. [Insurance / Indemnity 47](#bookmark129)
12. [Financial Aspects 48](#bookmark131)

[References 49](#bookmark133)

1. Background & Rationale

Pre-exposure prophylaxis (PrEP) is a useful prevention tool to offer young men and women.

Daily emtricitabine and tenofovir disoproxil fumarate (FTC-TDF) shows high efficacy in men who have sex with men (MSM)^1,2^ and heterosexual HIV-serodiscordant couples^3^, and is recommended by the World Health Organisation (WHO) for high-risk individuals.^4^ To be effective, daily PrEP requires good adherence^1^ as PrEP failure can lead to the selection of drug resistant HIV^2,5^ in the presence of intermittent dosing. Drug wastage can occur if taken during periods of low sexual risk, whilst bone and/or renal toxicity related to Tenofovir use can occur from long-term use as observed in HIV infected individuals.^6^ Bone toxicity is a particular concern for adolescents, as bone toxicity at this time may prevent achievement of peak bone mass predisposing to low bone density in adulthood^7^. In sub-Saharan Africa the background prevalence of low bone mineral density is 7% in the young and it is this very population where PrEP is proposed to play a key role in HIV prevention.^7^

Tenofovir alafenamide (TAF), a newer pro-drug of tenofovir with rapid and sustained cellular loading, has reduced renal and bone toxicity when compared to tenofovir and therefore can be used in established renal disease and requires less frequent monitoring.^6^ The enhanced safety profile and less frequent monitoring may reduce costs whilst allowing uptake in more diverse settings will, it is hoped, facilitate wider global coverage. Oral TAF PrEP protects monkeys from rectal infection, and efficacy studies of daily FTC-TAF in humans are underway.^8^

Oral FTC-TDF has been used as a daily formulation for HV treatment for over 15 years and for HIV prevention (daily and on demand PrEP) for 6 years. It is extremely safe to use and has over

1. million per years of follow up to support this.^9^ Oral FTC-TAF is better tolerated than FTC- TDF and has been licenced for two years for HIV treatment. The CHAPS study is evaluating a maximum of three tablets (of either FTC-TDF or FTC-TAF) per participant of these safe, and well tolerated drugs.

On-demand PrEP is when individuals anticipate their risk for HIV infection, and rather than taking PrEP continuously, only start a course of PrEP prior to times of sexual activity. This strategy is highly effective in MSM^10^ but widespread use is hindered by lack of data on efficacy in other populations, dosing and duration of protection, and regime complexity.^11^ Advantages of on-demand PrEP over daily PrEP include reduced toxicity, reduced drug wastage and improved cost effectiveness. Furthermore, on-demand PrEP has not led to drug resistance

which is highly relevant to sub-Saharan Africa and South/Southeast Asia where tenofovir resistance is emerging.^12^ The regime recommended for MSM involves dosing before and after sex with continuation of dosing if repeated exposures occur. Such regime complexity has raised concerns that it is not a feasible option for a wider population than highly educated MSM who were studied in France as part of the IPERGAY study.^10^ Regime simplification is thus desirable and we believe highly possible, particularly for insertive sex which is associated with a 10 fold less risk of HIV acquisition compared to those who practice receptive sex.^13^ The dosing requirement for on-demand PrEP for insertive sex is not known.

Many Phase 3 trials of daily PrEP have failed due to lack of adherence in the study population^14,15^, which has limited the ability to assess biological efficacy. Establishing biological efficacy and determining dose requirements before embarking on further large studies are essential to PrEP trial success. Ex-vivo challenge models provide a good measure of biological efficacy, are used to prioritize HIV-1 prevention candidates for Phase 3 trials^16-18^ and uniquely allow correlation of findings between animal and human models.

Post-exposure prophylaxis (PEP) is a further way to prevent HIV transmission and involves taking antiretroviral (ARV) drugs for 28 days after a potential exposure to HIV. Critically, it is not known how long after HIV exposure, through insertive sex, that PEP can be initiated effectively. Clearly placebo-controlled studies are not ethical and comparative treatment studies are prohibitively large. Evidence for the timing of PEP is based on cohort^19^ and non­human primate studies^20,21^ which define the window period for PEP to be started effectively for receptive anal sex to be within 24 hours^20^ and for receptive vaginal sex within 72 hours^21,22^. No data are available for insertive sex and given the differences in risk per exposure it is possible that window periods may differ between insertive and receptive sex. For both PrEP and PEP the relationship between tissue drug levels and HIV protection is poorly understood yet essential for optimizing therapies, adherence advice and safety.

This proposal aims to collect critical data from the foreskin explant HIV infection model to

optimize on-demand PrEP and PEP regimes for insertive sex, and to compare FTC-TAF with FTC- TDF (standard-of-care). It is the first study to evaluate PrEP dosing requirements specifically for

insertive sex.

1. Trial Objectives and Design
   1. Trial Objectives

**Overall aim**: Using the foreskin explant model, to investigate the optimum drug, dose and schedule for on-demand PrEP for insertive sex.

Primary objective:

To compare the effect of different PrEP drugs (FTC-TDF and FTC-TAF), doses and timing of doses on p24 antigen level in resected foreskin tissue following HIV exposure ex vivo challenge.

Secondary objectives

1. Investigate the timing and dose of FTC-TDF and FTC-TAF as oral PrEP and in vitro post exposure prophylaxis (PEP) (applied directly to removed foreskin tissue) required to prevent ex vivo HIV infection in foreskin tissue and blood using the explant model
2. Determine blood, rectal fluid and foreskin tissue concentrations of FTC, TFV, TAF and their active metabolites that are required for ex vivo HIV protection
3. Evaluate inflammation, cellular activation, foreskin mucosal integrity, gene expression and microbiome in foreskin tissue following oral in vivo PrEP and in vitro PEP
4. Evaluate the efficacy of in vitro post exposure dosing with PrEP in protection against ex vivo HIV infection using the explant model
5. To investigate sexual behaviour, PrEP acceptability and feedback on HIV prevention trials implementation
6. Primary endpoint

The primary endpoint is **non-infection of foreskin tissue with HIV** (in the lab) **at 15 days** following e*x vivo* challenge. This is because we wish to demonstrate the potential protective effect of the drugs.

1. Secondary endpoints

a. The mean p24 antigen level area under the curve (AUC) and p24 antigen slope from day 3 to day 15

b. Drug levels in plasma (TFV, FTC, TAF), peripheral blood mononuclear cells CD4 T cells (TFV-DP and FTC-TP), rectal fluid and foreskin Inflammation and cellular activation of foreskin tissue following PrEP initiation

4.2. Trial Design

This is an open-label, randomised control trial involving HIV negative men 13-24 years of age who are uncircumcised but are requesting voluntary male medical circumcision (VMMC) for HIV prevention. An unusual aspect of the trial is that foreskin tissue, rather than people will be used to ascertain protection against HIV infection. Young men will be randomised to different duration and doses of study drug prior to circumcision. Immediately following circumcision their foreskins will be collected, prepared and, in the explant HIV-infection model, foreskin tissue will be exposed to HIV. We will compare three durations of FTC-TDF or FTC-TAF respectively simulating on demand PrEP. The trial will take place in Entebbe, Uganda and in Soweto, South Africa.

Ex vivo challenge validation

Part of the purpose of this study is to empower research sites. Therefore, after appropriate training, mentorship and site preparation, the ex vivo model will have to be validated at each site in order to progress to the main study. Three men will be recruited at a time. These individuals will not be randomised and will not receive PrEP prior to VMMC. Their foreskin tissue will be exposed to HIV through the ex vivo challenge. Successful infection of the excised foreskin tissue of at least three consecutive men using ex vivo challenge is required to validate the site. A maximum total sample size of 12 men will be recruited for validating the methods.

Randomised trial

A total of 144 eligible individuals (72 per country: South Africa and Uganda) will be initially approached and consented as described in Section 6.3. If eligible, they will be randomised in equal numbers to one of 9 treatment arms, each comprising a total of 16 evaluable participants, 8 per country. Four treatment arms will receive FTC-TDF (two dosing schedules, two timing schedules), four arms will receive FTC-TAF (schedules the same as for FTC-TDF) and one group will be the control group who will receive no PrEP (Figure 1, Table 1).

According to randomisation arm, circumcision will either be five or 21 hours after the last dose of PrEP has been given. Participants will take tablets at each clinic site under directly observed therapy (DOT).

Participants in the control arm will have their circumcision soon after randomisation, they will not have to wait for five hours. At the appropriate time after dosing, all other participants receiving PrEP will undergo VMMC. Their foreskins will be collected, and transferred to the local lab within two hours of circumcision. At the lab, foreskins will be processed and used for the explant HIV-infection models. Local scientists trained by Imperial College staff from the UK will conduct experiments. Protection from HIV infection will be determined by ex vivo challenge using wild type HIV virus on freshly resected foreskins.^16-19^ Participants who do not take their full course of treatment, or choose to delay circumcision will be deemed to be un-evaluable and will be replaced. We will randomise a maximum of 100 consenting men to obtain 72 evaluable participants.

Social science

On the day of randomisation, all participants will complete a socio-behavioural survey, including demographics and sexual behaviour as well as a short willingness to participate survey. In addition, user feedback, using a short exit survey to gain insights on the clinical trial implementation and future use/perceptions of PrEP, will be conducted at the VMMC visit or the final visit (post VMMC/final study visit) by all participants. To gain qualitative feedback on the clinical trial implementation and use/perceptions of PrEP, a sub set of participants (n=20-25 per site, respectively) and healthcare providers (n=3-5 per site, respectively) will be asked to participate in an In-depth Interview (IDI) on either the VMMC visit or the final study visit (post VMMC/final study visit). The participant will be purposively sampled to capture respondents at different ages and from all the dosing regimen.

Table 1: Summary of randomisation arms

| **Randomised**  **arm** | **Drug** | **N** | **Dose 1** | **Dose 2 (24 hours later;**  (+/-1hr) | **Interval between last PrEP dose and surgery (hours)**  (+/-1hr) | **Analysis Group (Figure 1)** |
| --- | --- | --- | --- | --- | --- | --- |
| 1 | Control | 16 | - | - | - | X |
| 2 | FTC-TDF | 16 | 2 tablets | - | 5 | A |
| 3 | FTC-TDF | 16 | 2 tablets | - | 21 | A |
| 4 | FTC-TDF | 16 | 2 tablets | 1 tablet | 5 | B |
| 5 | FTC-TDF | 16 | 2 tablets | 1 tablet | 21 | B |
| 6 | FTC-TAF | 16 | 2 tablets | - | 5 | C |
| 7 | FTC-TAF | 16 | 2 tablets | - | 21 | C |
| 8 | FTC-TAF | 16 | 2 tablets | 1 tablet | 5 | D |
| 9 | FTC-TAF | 16 | 2 tablets | 1 tablet | 21 | D |


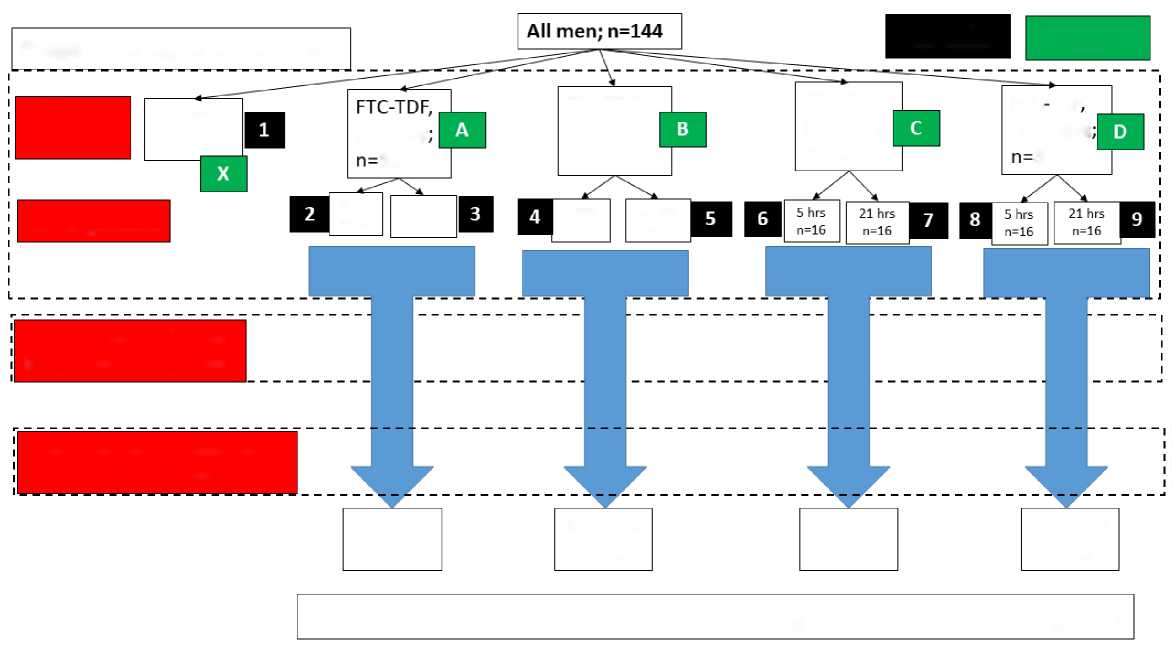


**Figure 1. Flow diagram of participant and foreskin journey for primary analysis (oral PrEP only).**

**Oral PrEP dosing**

**Circumcision followed by *ex vivo* HIV infection**

5 hrs n=16

21 hrs n=16

21 hrs n=16

5 hrs n=16

FTC-TDF,

two days; n=32

**Primary analysis: Four active treatment groups; n=32 in each group**

**Lab analysis to determine HIV infection status**

**Stage 1 - drug taken by men**

**Randomisation Group identifier**

**Analysis Group identifier**

**Interval between last dose and circumcision**

Group A n=32

Group C n=32

Group B n=32

Group D n=32

FTC

TAF

days

two

32

FTC-TAF,

one day;

n=32

day

one

32

No PrEP; n=16

Figure 1. Flow diagram of participant and foreskin journey for primary analysis (oral PrEP only). Numbers in the black boxes indicate the randomisation

groups - there are nine, with each man allocated to exactly one group, with each group equally likely. This figure shows recruitment from both sites;

recruitment will be split equally between South Africa (n=72) and Uganda (n=72). Numbers in green boxes refer to analysis groups. The main comparison will be between groups A-D. Group X, men who do not receive PrEP, will be used as a comparison and to ensure that the ex-vivo challenge model is successful.

Figure 2. Flow diagram of participant and foreskin journey for oral PrEP, in vitro drug exposure and ex vivo HIV challenge.

**Analysis Group identifier**

**Randomisation Group identifier**

**Oral PrEP dosing**

**Circumcision followed by ex *vivo* HIV infection**

***in vitro***

**drug**

**A-5-TDF**

**B-5-0**

**X-0**

**X-TDF**

**X-TAF**

**A-5-0**

**C-5-TAF**

**D-5-TAF**

**D-5-0**

**B-21-0**

**A-21-TDF**

**A-21-0**

**C-21-TAF**

**D-21-0**

**D-21-TAF**

No PrEP

**FTC-TDF**

**S hrs n=16**

**21 hrs n=16**

**5 hrs n=16**

**21 hrs n=16**

**5 hrs n=16**

**21 hrs n=16**

**F/TAF**

No PrEP

**FTC-TDF**

FTC-TAF

No PrEP; n=16

**5 hrs n=16**

**21 hrs n=16**

**Stage 2 - drug given to foreskin ex *vivo***

**Lab analysis to determine HIV infection status**

**Stage 1 - drug taken by men**

**Interval between last dose and circumcision**

**All men; n=144**

**B-5-TDF**

**B-21-TDF**

**C-5-0**

**C-21-0**

**FTC-TDF**

No PrEP

No PrEP

No

PrEP

F/TAF

FTC

TAF

day

one

32

FTC

TAF

days

two

32

day

one

32

FTC

TDF

days

two

32

Figure 2. Flow diagram of participant and foreskin journey for oral PrEP, in vitro drug exposure and ex vivo HIV challenge. The Analysis Group Identifiers in Stage 2 comprise three components: A letter indicating the Stage 1 group; a number (5 or 21) indicating the interval between last oral PrEP dose and circumcision; and an item indicating in vitro dosing (where 0=no dose). The exceptions are the group of men who did not receive oral PrEP (Group X):

these groups do not include the middle component.


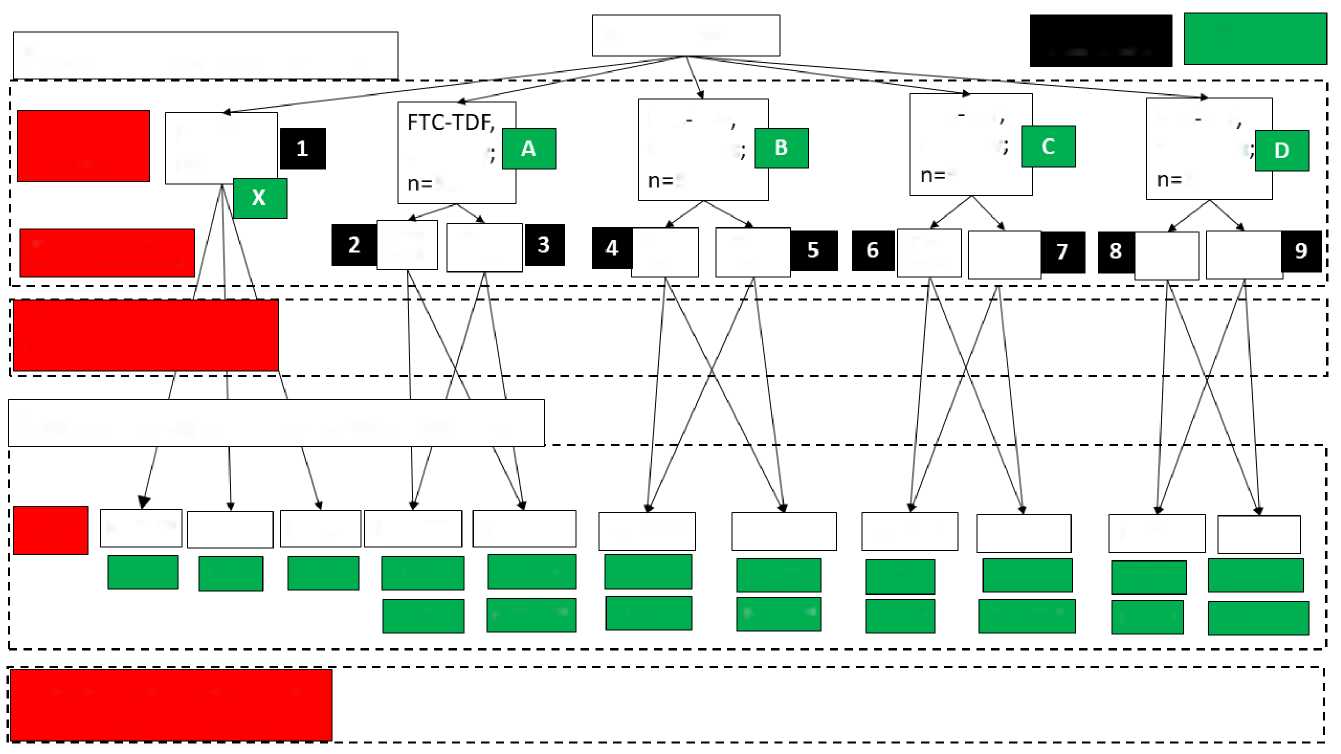


Table 2.1: Table of participant randomisation groups and laboratory analysis of foreskins

| **Stage 1: Oral PrEP taken by men** | | | | | | **Foreskin tissue divided in the lab** | **Stage 2: *In vitro* dosing of tissue in t** | | **he laboratory** |
| --- | --- | --- | --- | --- | --- | --- | --- | --- | --- |
| **Rand**  **arm** | **N** | **Oral PrEP analysis group** | **Oral drug** | **Number of PrEP doses** | **Interval between last oral PrEP and VMMC (hours)** |  | ***in vitro* analysis group** | ***in vitro* drug** | **Interval between *ex vivo* HIV exposure and *in vitro* dose (hours)** |
| 1 | 16 | X | No PrEP | - | - |  | X-0 | No PrEP | - |
|  |  |  |  |  |  |  | X-TDF | FTC-TDF | 18 |
|  |  |  |  |  |  |  | X-TAF | FTC-TAF | 18 |
| 2 | 16 | A | FTC-TDF | 1 | 5 |  | A-5-0 | No PrEP | - |
|  |  |  |  |  |  |  | A-5-TDF | FTC-TDF | 18 |
| 3 | 16 |  | FTC-TDF | 1 | 21 |  | A-21-0 | No PrEP | - |
|  |  |  |  |  |  |  | A-21-TDF | FTC-TDF | 18 |
| 4 | 16 | B | FTC-TDF | 2 | 5 |  | B-5-0 | No PrEP | - |
|  |  |  |  |  |  |  | B-5-TDF | FTC-TDF | 18 |
| 5 | 16 |  | FTC-TDF | 2 | 21 |  | B-21-0 | No PrEP | - |
|  |  |  |  |  |  |  | B-21-TDF | FTC-TDF | 18 |
| 6 | 16 | C | FTC-TAF | 1 | 5 |  | C-5-0 | No PrEP | - |
|  |  |  |  |  |  |  | C-5-TAF | FTC-TAF | 18 |
| 7 | 16 |  | FTC-TAF | 1 | 21 |  | C-21-0 | No PrEP | - |
|  |  |  |  |  |  |  | C-21-TAF | FTC-TAF | 18 |
| 8 | 16 | D | FTC-TAF | 2 | 5 |  | D-5-0 | No PrEP | - |
|  |  |  |  |  |  |  | D-5-TAF | FTC-TAF | 18 |
| 9 | 16 |  | FTC-TAF | 2 | 21 |  | D-21-0 | No PrEP | - |
|  |  |  |  |  |  |  | D-21-TAF | FTC-TAF | 18 |
| Note: T | he in vitro Analysis Groups comprise three components: A letter ind | | | | | icating the Stage 1 group; a number (5 or 21) indicating th | | | e interval between |

last oral PrEP dose and circumcision; and an item indicating in vitro dosing (where 0=no dose). The exceptions are the group of men who did not receive oral PrEP (Group X): these groups do not include the middle component.

Table 2.2 Additional laboratory analysis of foreskin tissue in men who did not receive PrEP

| **In vitro dosing of HIV drug** | |  |
| --- | --- | --- |
| **Drug** | **Hours after ex vivo HIV exposure** | **_N_1** |
| No PrEP | No PrEP | 16 |
| FTC-TDF | 2 hrs | 16 |
|  | 24 hrs | 16 |
|  | 48 hrs | 16 |
|  | 72 hrs | 16 |
| FTC-TAF | 2 hrs | 16 |
|  | 24 hrs | 16 |
|  | 48 hrs | 16 |
|  | 72 hrs | 16 |

1. Note that N=16 for all groups, as this is how many men did not receive oral PrEP. Each man's foreskin will be divided as necessary to allow *in vitro* dosing experiments.

Table 3.1: Visit schedule randomisation arm 1 control

| **Visit number** | **0** | **1** | **2** | **3** |
| --- | --- | --- | --- | --- |
|  | **Screening** | **Randomisation** | **VMMC** | **Post**  **VMMC** |
| Day | -1 | 0 | 1 | 7-21 |
| Assessment of understanding | X |  |  |  |
| Medical history | X |  |  |  |
| Haemoglobin | X |  |  |  |
| HIV test | X |  |  |  |
| Informed consent | X |  |  |  |
| Conmeds | X |  |  | X |
| Physical examination | X |  | X | X |
| Randomisation* |  | X |  |  |
| Willingness to participate survey |  | X |  |  |
| Socio-behavioural survey |  | X |  |  |
| SAE monitoring |  |  | X | X |
| RPR |  |  | X |  |
| Urine STI screen (first-catch sample) |  |  |  |  |
| Research Blood:  - PBMCs for PK/PD  - Plasma for cytokines |  | X | X |  |
| Research urethral swab x 1 |  |  | X |  |
| Research rectal swab x 1 |  |  | X |  |
| Research glans microbiome swab x 1 |  |  | X |  |
| Post - operative review |  |  |  | X |
| Exit Survey |  |  |  | X |
| Exit In-depth Interview |  |  |  | X |

Table 3.2: Visit schedule for randomisation arms 2 and 6 (double dose PrEP with VMMC 5 hours later)

| **Visit number** | **0** | **1** | **2** | **3** | **4** |
| --- | --- | --- | --- | --- | --- |
|  | **Screening** | **Randomisation** | **PrEP dosing** | **VMMC 5 hours after last PrEP dose** | **Post**  **VMMC** |
| Day | -1 | 0 | 1 | 2 | 7-21 |
| Medical history | X |  |  |  |  |
| Haemoglobin | X |  |  |  |  |
| HIV test | X |  |  |  |  |
| Consent | X |  |  |  |  |
| Assessment of understanding | X |  |  |  |  |
| Physical examination | X |  |  | X | X |
| Willingness to participate survey |  | X |  |  |  |
| Randomisation |  | X |  |  |  |
| Socio-behavioural survey |  | X |  |  |  |
| Directly observed PrEP dosing* |  |  | X |  |  |
| SAE monitoring |  |  | X | X | X |
| Conmeds | X |  | X | X | X |
| RPR |  |  |  | X |  |
| Urine  - STI screen (first-catch sample  - Research (mid stream |  |  |  | X |  |
| Research Blood - PBMCs for PK/PD - Plasma for cytokines  and PK |  | X |  | X |  |
| Research rectal swab x1 |  |  |  | X |  |
| Research urethral swab x1 |  |  |  | X |  |
| Research glans microbiome swab x 1 |  |  |  | X |  |
| Post - operative review |  |  |  |  | X |
| Exit survey |  |  |  |  | X |
| Exit in-depth interview |  |  |  |  | X |

Table 3.3: Visit schedule for randomisation arms 3 and 7 (double dose PrEP with VMMC 21 hours later)

| **Visit number** | **0** | **1** | **2** | **3** | **4** |
| --- | --- | --- | --- | --- | --- |
|  | **Screening** | **Randomisation** | **PrEP**  **dosing** | **VMMC 21 hours after last PrEP dose** | **Post**  **VMMC** |
| Day | -1 | 0 | 1 | 2 | 7-21 |
| Medical history | X |  |  |  |  |
| HIV test | X |  |  |  |  |
| Haemoglobin | X |  |  |  |  |
| Consent | X |  |  |  |  |
| Assessment of understanding | X |  |  |  |  |
| Physical examination | X |  |  | X | X |
| Willingness to participate survey |  | X |  |  |  |
| Socio-behavioural survey |  | X |  |  |  |
| Randomisation* |  | X |  |  |  |
| Directly observed PrEP dosing |  |  | X |  |  |
| SAE monitoring |  |  | X | X | X |
| Conmeds | X |  | X | X | X |
| RPR |  |  |  | X |  |
| Urine  - STI screen (first-catch sample  - Research (mid stream) |  |  |  | X |  |
| Research Blood - PBMCs for PK/PD - Plasma for cytokines and  PK |  | x |  | X |  |
| Research rectal swab x1 |  |  |  | X |  |
| Research urethral swab x 1 |  |  |  | X |  |
| Research glans microbiome swab x 1 |  |  |  | X |  |
| Post - operative review |  |  |  |  | X |
| Exit survey |  |  |  |  | X |
| Exit in-depth interview |  |  |  |  | X |

*randomisation will take place after eligibility has been confirmed

Table 3.4: Visit schedule for randomisation arms 4 and 8 (2+1 dosing with VMMC 5 hours later)

| **Visit number** | **0** | **1** | **2** | **3** | **4** | **5** |
| --- | --- | --- | --- | --- | --- | --- |
|  | **Screening** | **Randomisation** | **1^st^ PrEP dosing visit** | **2^nd^ PrEP dosing visit** | **VMMC 5 hours after last PrEP dose** | **Post**  **VMMC** |
| Day | -1 | 0 | 1 | 2 | 2 | 7-21 |
| Medical history | X |  |  |  |  |  |
| Physical examination | X |  |  |  | X | X |
| HIV test | X |  |  |  |  |  |
| Haemoglobin | X |  |  |  |  |  |
| Consent | X |  |  |  |  |  |
| Assessment of understanding | X |  |  |  |  |  |
| Socio-behavioural  survey |  | X |  |  |  |  |
| Willingness to participate survey |  | X |  |  |  |  |
| Randomisation* |  | X |  |  |  |  |
| Directly observed PrEP |  |  | X | X |  |  |
| SAE monitoring |  |  | X | X | X | X |
| Conmeds | X |  | X | X | X | X |
| RPR |  |  |  |  | X |  |
| Urine  - STI screen (first- catch sample  - Research (mid stream) |  |  |  |  | X |  |
| Research Blood - PBMCs for PK/PD - Plasma for cytokines and PK |  | X |  |  | X |  |
| Research rectal swab x1 |  |  |  |  | X |  |
| Research urethral swab x 1 |  |  |  |  | X |  |
| Research glans microbiome swab x 1 |  |  |  |  | X |  |
| Post - operative review |  |  |  |  |  | X |
| Exit survey |  |  |  |  |  | X |
| Exit in-depth interview |  |  |  |  |  | X |

Table 3.5 Visit schedule for randomisation arms 5 and 9 (2+1 dosing with VMMC 21 hours later)

| **Visit number** | **0** | **1** | **2** | **3** | **4** | **5** |
| --- | --- | --- | --- | --- | --- | --- |
|  | **Screening** | **Randomisation** | **First**  **dosing**  **visit** | **Second**  **dosing**  **visit** | **VMMC 21 hours after last PrEP dose** | **Post**  **VMMC** |
| Day (Acceptable window) | -1 | 0 | 1  (0-21) | 2 | 3 | 7 (6-21) |
| Consent | X |  |  |  |  |  |
| Risk assessment | X |  |  |  |  |  |
| Physical examination | X |  |  |  | X | X |
| Medical history | X |  |  |  |  |  |
| HIV test | X |  |  |  |  |  |
| Haemoglobin | X |  |  |  |  |  |
| Assessment of understanding | X |  |  |  |  | X |
| Willingness to  participate survey |  | X |  |  |  |  |
| Socio-behavioural  survey |  | X |  |  |  |  |
| Randomisation* |  | X |  |  |  |  |
| Directly observed PrEP dosing |  |  | X | X |  |  |
| SAE monitoring |  |  | X | X | X | X |
| Conmeds | X |  | X | X | X | X |
| RPR |  |  |  |  | X |  |
| Urine  - STI screen (first- catch sample  - Research (mid stream ) |  |  |  |  | X |  |
| Research Blood - PBMCs for PK/PD - Plasma for  cytokines and PK |  | X |  |  | X |  |
| Rectal swab x1 |  |  |  |  | X |  |
| Urethral swab x1 |  |  |  |  | X |  |
| Glans swab x 1 |  |  |  |  | X |  |
| Post - operative review |  |  |  |  |  | X |
| Exit survey |  |  |  |  |  | X |
| Exit in-depth interview |  |  |  |  |  | X |

1. Trial Medication
   1. Investigational Medicinal Product (IMP)

Truvada® (Emtricitabine 200mg/Tenofovir disoproxil fumarate 300mg). A blue, capsule-shaped, film-coated tablet, debossed on one side with “GILEAD” and on the other side with “701. Supplied in a high density polyethylene (HDPE) bottle with a polypropylene child-resistant closure containing film-coated tablets and a silica gel desiccant.

Descovy® or FTC-TAF (Emtricitabine 200mg/ Tenofovir Alafenamide 25mg. A blue, rectangular­shaped, film-coated tablet debossed with “GSI” on one side and “225” on the other side of the tablet. Supplied in a high-density polyethylene (HDPE) bottle with a polypropylene child-resistant cap containing film-coated tablets and a silica gel desiccant and polyester coil.

Gilead Sciences will provide commercially available product directly to clincal trial sites. Drug receipt and site dispensing will be recorded. All study drugs will be dispensed as Clinical Trial stock by the pharmacist located in the research pharmacy, against a Trial Prescription Form maintaining full drug accountability.

- 1. Dosing Regimen

FTC-TDF and FTC-TAF will be evaluated according to the daily dosing schedules outlined previously (Table 1 and 2). All regimes are taken once a day as directly observed therapy (DOT) doses administered by research staff. Those randomised to two doses in one day will receive both tablets at the same time as a double dose.

- 1. IMP Risks

The Summary of Product Characteristics (SmPC) for FTAF^®^ and FTC-TDF^®^ provided by the manufacturer will act as the reference documents. We do not anticipate any interaction concerns - participant with co-morbidities are not usually candidates for outpatient circumcision. Participants will be excluded if they have a medical history which precludes either of these two IMPs. the IMP are both extremely well tolerated during long term use for HIV treatment and HIV prevention as daily therapy. For this study, a participant will receive a

maximum of three tablets. The following safety data refers to long term daily use and not such short usage:

Emtricitabine TAF and tenofovir are primarily excreted by the kidneys. TAF is a pro-drug of TDF with less renal toxicity. Renal failure, renal impairment, elevated creatinine, hypophosphataemia and proximal tubulopathy have been reported with the use of tenofovir dispoproxil fumarate. Avoid concurrent use of nephrotoxic medicinal products and medicines that reduce renal function or compete for active tubular secretion.

TDF/FTC Adverse reactions reported from daily use:

Common: headache, dizziness, diarrhoea, vomiting, nausea, rash, increased creatine kinase and asthenia.

Uncommon: neutropenia, allergic reactions (including pruritis and urticaria), skin discolouration (increased pigmentation), hyperglycaemia, insomnia, abnormal dreams, elevated AST/ALT, pain, anaemia, pancreatitis, angioedema, increased creatinine, proteinuria.

Rare: hepatic steatosis, hepatitis, osteomalacia, renal failure, nephritis, acute tubular necrosis and nephrogenic diabetes insipidus.

Descovy Adverse reactions:

Common: nausea. abnormal dreams, headache, dizziness, diarrhoea, vomiting, abdominal pain, flatulence, rash and fatigue.

Uncommon: anaemia, dyspepsia, angioedema, pruritis and arthralgia.

- 1. Drug Accountability

Each local pharmacy will keep accountability records for reconciliation purposes; This will be used to record the identification of the participant to whom the investigational product was dispensed, the date, batch number, expiry date and quantity of the investigational product dispensed and the quantity of the investigational product unused/returned. Study staff doing DOT will be asked to return empty packaging to pharmacy for accountability. All drug accountability records will be available for verification by the study monitor.

- 1. Storage of IMP

As detailed in the summary of product characteristics (SmPC) for FTC-TDF® and FTAF® tablets do not require special storage conditions. All study drugs for oral use will be kept in a secure pharmacy until they are dispensed for DOT. Ex vivo dosing will be carried out using base compound (FTC-TDF and FTAF powder)- these drugs will be dissolved in PBS, aliquoted and stored at -20C.

- 1. Dosing Schedule

The administration of PrEP and timing of VMMC will be planned between the participant and research team according to the randomisation arm. The DOT PrEP dose should be given within +/-1 hour of expected next dose. VMMC should be done either 5h or 21h (both with windows of +/- 1 hour) after the last PrEP dose. If an individual is outside the window for VMMC then the individual is either removed from the study or the dosing and procedures are repeated after a minimum of a one-week washout (section 6.5).

- 1. Concomitant Medication

All concomitant medication will be recorded. Concomitant therapies will be managed in line with the Summary of Product Characteristics guidance for FTC-TDF^®^ and FTC-TAF.^®^

1. Selection and Withdrawal of Participants
   1. Inclusion Criteria

Participants must satisfy all the following criteria within 21 days prior to their circumcision

visit:

1. Clinically eligible for either forceps guided, or dorsal slit circumcision
2. The ability to understand and sign a written informed consent form by participant (**and**

participant's legal guardian if younger than 18 years) prior to participation in any study procedures and to comply with all trial requirements

1. Male sex at birth
2. Age 13- 24 years
3. Haemoglobin >9g/dL
4. Weight >35Kg
5. Two negative rapid HIV antibody tests results (manufactured by different companies), dating from 21 days or less prior to VMMC.
6. Two locator information details (including physical address, telephone contacts, email) for contacting of either patient or their parent
   1. Exclusion Criteria

1. Any significant acute or chronic medical illness and current therapy that in the opinion of the site investigator would preclude receipt either of: investigational products, or VMMC

2. Any evidence participant is not suitable for VMMC

- 1. Selection of Participants

Recruitment will take place from the community as well as those attending VMMC clinics. The trial sites are the Chris Hani Baragwanath Academic Hospital, Soweto, South Africa and the Entebbe Regional referral Hospital and the communities around Entebbe, Uganda.. Potentially eligible participants will be provided with an information sheet, detailing the trial aims, its procedures and medication to be investigated, and given time to consider their participation prior to arranging an appointment for a screening visit. At this visit, before undertaking any screening procedures, volunteers will be able to discuss the trial to ensure they have enough information to consider whether they would like to participate or not. An important part of the screening visit will be HIV testing (described in Section 7.1). All trial staff will be experienced healthcare practitioners, trained to conduct HIV pre and post-test counselling. Once individuals feel ready to participate in the trial they will provide full written consent to participate. The teams will endeavour to recruit approximately 25% 13-17 year olds and ~75% aged 18-24 by recruiting from a range of different settings.

- 1. Randomisation Procedure

Enrolled participants will be randomized prospectively into one of 9 groups in equal ratios, stratified by country (n=144; 8 participants per group per country). Randomisation will be performed separately in each country, each using blocks of size 9 and 18, so that one or two men in each block will be assigned to each of the nine groups. The randomisation schedule will be assigned in London by the trial statistician. The trial statistician will send lists to each country to be pre-prepared into consecutive sealed randomisation envelopes with country and study ID's printed on the outside and containing the allocated trial arm. After a participant is successfully screened, the next envelope will be opened, and the man allocated to the appropriate trial arm. This is not a blinded study therefore participants and research team will know which medications the patient is receiving.

When the intended number of randomisations have been performed, some groups may be incomplete due to withdrawals, lost to follow-up, or laboratory need. At this stage a new randomisation list will be drawn up (by the trial statistician) and men will be recruited to reach the intended sample size for each arm. By adding men at this stage, it will be possible to maintain allocation concealment.

- 1. Withdrawal of Participants

Participants have the right to withdraw from the trial at any time for any reason without facing any penalty, and without compromising their usual standard of care at the health care facility. The investigator may also withdraw participants from the trial in the event of inter-current illness, adverse events, protocol violations, administrative or other reasons. Those withdrawing prior to VMMC will be replaced.

Participants can be withdrawn from the study in any of the following circumstances:

- More than +/-1 hour minutes late for a PrEP dosing visit - Outside the window period for VMMC.

If this occurs, then the participant will be replaced through a second randomisation procedure as above (section 6.4) OR they will have a one week wash out period and the PrEP dosing commenced again.

In all cases the date and reasons for withdrawal or withholding the dose of medication will be clearly stated on the participant's CRF. Prior to withdrawal from the study participants will be asked whether they give permission for data collected prior to their withdrawal to be used. If the reason for removal of a participant from the trial is an adverse event, the principal specific event will be recorded on the CRF.

- 1. Expected Duration of Trial

The trial is expected to last 12 months from the first participant's first visit to the last participant's last follow up visit. From signing consent to completing participation in the trial, each participant will be on study for approximately three weeks.

1. By Visit

Participants will attend for a maximum of 6 study visits, depending on randomisation arm. Once screened, the remaining four visits will take place over a one-two week period. The schedule of assessments is summarised in separate visit schedules for each group. Reimbursement for time and transport will occur at each visit. A description of the visits is listed below.

1. Screening Visit

Prior to the screening visit participants will be provided with written information about the trial in the form of a participant information sheet and will be allowed adequate time for questions and to consider the trial before agreeing to participate. It will be the responsibility of the investigator or designee to obtain written informed consent and assent where applicable, prior to undertaking any procedures detailed in the protocol.

The investigator or designee must provide adequate explanation of the aims, methods, objectives and potential risks and benefits of the trial. It must also be explained to the participant that they are free to refuse or withdraw from the trial for any reason without detriment to their future care or treatment.

Willing participants will be required to read, sign and date an informed consent document prior to any trial related procedures being performed. All willing participants will have to demonstrate that they have understood the information before they sign. In the case of those who are illiterate, the informed consent document will be read to the participant in the presence of an independent witness. The participant thumbprints will then be documented as consent while the witness and staff will countersign. Participants will be given the opportunity to ask questions regarding the trial at any time during the trial period.

All participants less than 18 years old will be required to provide parental consent for participation in clinical research. All participants will be reimbursed for their travel expenses for all scheduled visits.

Screening evaluations will be used to determine the eligibility. Following informed consent, the screening visit will evaluate:

- Physical examination including measurement of weight, height, and vital signs (temperature, blood pressure)
- Assessment of concomitant medications
- Prior medical history
- Current symptoms - both systemic and those related to the genito-urinary tract
- Blood drawn for haemoglobin levels
- HIV testing using two point of care HIV tests (POCT). Any volunteers found to be infected with HIV- 1 at screening will be referred for management according to local standard of care (section 7.2)
- Clinical eligibility for circumcision assessed using local VMMC guidelines.

For subsequent visits, participants will be requested to attend the research unit at a specified time based on their randomisation arms and dosing schedules.

1. Randomisation visit

Once eligibility has been confirmed randomisation will take place. Consecutive sealed randomisation envelopes with study ID's printed and containing the allocated arm will be used (Section 6.4). After each randomisation, the participant ID and the allocated trial arm will be entered into a Log. This will be checked each week against the randomisation list prepared by the Trial Statistician.

For men allocated to randomisation arm 1, the control arm (no PrEP dosing), VMMC can occur on that day or up to 21 days following randomisation. For men allocated to randomisation arms 2-9 (PrEP dosing), PrEP dosing can occur up to 21 days following randomisation.

For men allocated to Arms 2-9 (PrEP dosing), DOT PrEP will be administered at the clinic. DOT entails a nurse overseeing the receipt and ingestion of study medication and documenting in the source document the time, the dose, and that the dose was ingested by the participant. The VMMC must occur up to 21 days following randomisation. Subsequent visits will be arranged according to randomisation group.

At the day of randomisation, all participants will also complete a self-or interviewer administered socio-behavioural survey and a short willingness to participate survey, which will include the following sections: socio-demographics, sexual behaviour, reasons to participate in the trial, attitudes towards PrEP and HIV prevention preferences. The survey will be translated into the main local languages of each country (South Africa: Zulu, Sesotho; Uganda: Luganda). All participants will complete this interviewer-administered and structured survey, using the electronic database, iDatafax. The survey will last approximately 20 minutes and will be accessed using a tablet, desktop computer or laptop. Surveys will be completed as part of the study procedures on the day of randomisation in a private room at the research sites.

A 9ml blood sample will be taken for research

1. DOT PrEP dosing visits +/-1 hour of expected next dose

The following evaluations will take place prior to next dose:

- AE review. If significant adverse events have been reported, these will be clinically followed in accordance to the instruction of the study physician.
- Concomitant medications review

1. VMMC sample collection visit

The following evaluations will take place prior to VMMC and the time of sample collection will be accurately recorded:

- Physical / genital exam if clinically indicated
- Concomitant medications review
- AE review. If significant adverse events have been reported, these will be clinically followed in accordance to the instruction of the study physician
- Samples taken +/- 40 mins before or after VMMC as follows:
- Urine for chlamydia and gonorrhoea assay (self-taken)
- Urine (5 mLs) for PK analysis (self-taken)
- Blood sample (60mls) for RPR, PK and PD analysis
- Optional rectal swab for PK analysis (self-taken or researcher taken)
- Samples taken immediately before VMMC as follows:
- One urethral swab for cytokine analysis will be done immediately prior to cleaning and prepartion of the penis (researcher-taken)
- One Diagene swab of the Coronal Sulcus at MMC for microbiome analysis (researcher-taken)

VMMC will be carried out according to local guidelines and the time of removal of foreskin will be accurately recorded. In brief, a sterile surgical pack containing all the necessary consumables is opened for each participant, after physical examination of the penis to ensure that no anatomical abnormalities or evidence of STI are found. A nurse instils local anaesthetic around the base of the penis and using povidone iodine solution, cleans and prepares the penis for surgery. Approximately 10-15 minutes later, the operator (either a doctor or clinical associate) will test anaesthesia, and if complete, will clamp the foreskin just above the glans and excise of

the distal foreskin immediately thereafter. Four stay sutures at each quadrant of the wound are placed by the operator after using diathermy to stop any bleeding points; the intervening surgical wound is then sutured. If the forceps guided method is not indicated, a dorsal slit circumcision will be done. The wound is dressed and once the participant feels up to it, he walks to the recovery room and waits there for an hour. Analgesics are dispensed together with wound care instructions. Men will be not advised to resume intercourse until six weeks after circumcision and to use condoms. The resected foreskin will be transferred on wet ice or a cool pack to the laboratory immediately. The foreskin must arrive at the laboratory within two hours of VMMC.

*Exit survey and procedures*

At the VMMC visit or the final study visit (post VMMC), all participants will complete a self-or interviewer administered short exit survey using the electronic database, ODK and/or iDatafax. Questions will focus on motivation and concerns for trial participation, sexual behaviour, PrEP acceptability (for participants who took PrEP) and clinical trial experience. The survey will take approximately 5 minutes to complete. The exit survey will be translated into the main local languages of each country (South Africa: Zulu, Sesotho; Uganda: Luganda).

*In-Depth Interview procedures*

In those consenting, IDIs will be conducted to explore in-depth perspectives and attitudes towards HIV risk, acceptability of young people in a PrEP clinical trial and use/perceptions of PrEP. These will take place at an arranged time 1 to 4 weeks following VMMC. Young men aged 13-24 years (n=20-25 per site), who participated in the clinical trial and clinical trial health care providers (n=3-5 per site) will be asked to take part in a once-off IDI. IDIs will take place on the day of the VMMC visit or on the day of the final study visit (post VMMC). Interviews will be conducted face-to-face by trained research assistants in a private room at the research sites. Interviews will be audio-recorded and conducted in the country's local languages and English, as preferred by participants. IDIs will last for approximately 30 minutes and separate participant consent or assent for IDIs and audio-recording will be obtained prior to the interview. Parental/guardian consent will be obtained for participants younger than 18 years. Additionally, parent/guardian consent for audio recording will be completed.

*Semi-structured interview guide for IDIs*

A semi-structured interview guide will be used for IDIs with trial participants and health care providers to investigate a number of questions including adolescents' attitudes and experience of the clinical trial conduct, experience/perceptions of PrEP use, sexual behaviour, and to explore how prevention interventions can be improved to recruit and retain participants in clinical trials. The interview guide will be pilot tested among the adolescent and adult community advisory boards in each country.

1. Exit safety visit after VMMC

- Physical / genital exam if clinically indicated
- Concomitant medications review
- AE review. If adverse events have been reported, these will be clinically followed in accordance to the instruction of the study physician.

1. Laboratory Tests

Safety and eligibility samples

All results will be recorded on the eCRF. The investigator must review, sign and date the laboratory results/reports, comment whether any abnormal values are clinically significant and record any clinically relevant changes occurring during the study in the adverse event section of the eCRF.

- HIV-1 risk assessment and HIV-1 testing

Site personnel will assess volunteers for past and current risk of HIV infection. Additionally, site personnel will perform pre-HIV test counselling. Two rapid HIV tests performed according to manufactures specifications, that both are clearly negative will be used as evidence of absence of HIV infection. In the event of a volunteer being diagnosed HIV-1 positive at screening they will immediately be referred to the nearest facility with a copy of their results and a referral letter for further counselling and care.

- STI screening

First-catch urine samples (5ml) will be analysed locally for gonorrhoea and chlamydia. In the event of an infection is detected, participants will be recalled for treatment according to local guidelines and contact tracing carried out.

- Serum haemoglobin

A blood sample will be taken and sent to the routine laboratory for analysis.

1. Clinical Research sample processing

Clinical samples taken at VMMC visit must be transferred immediately to the local laboratory so that they are received within 2 hours of sample collection. The methods for sample collection and packaging for transfer are described in the operations manual.

Laboratory Research Sample analysis

It is not possible for laboratories to be blinded to the participants' arm allocation due to the

different laboratory experiments carried out on controls versus cases as well in vitro dosing of

FTC-TDF versus FTC-TAF. The time of day a sample is received will also be dictated by study arm.

1. Blood sample

Plasma, CD4+ expressing cells and PBMCs will be extracted from 50mls whole blood. Ex vivo challenge of PBMCs will occur immediately. The remaining PBMCs and Plasma samples will be stored for drug level and cytokine analysis. Should there be sufficient sample remaining after the other analyses, we will also test for the presence of SARS-CoV-2 antibodies using samples taken prior to circumcision. This was requested because of concerns relating to inflammation related to COVID-19 which may impact our primary outcome, and also high rates of asymptomatic SARS-CoV-2 infections reported in South Africa and elsewhere which may be detected on antibody testing.

1. Foreskin

Immediately upon arrival in the laboratory, foreskin tissue will be cut and divided into sections to investigate ex vivo challenge, pharmacokinetics, inflammation and microbiome.

1. Urethral swab

Samples will be transported to the University of Cape Town after 50% recruitment and at the end of the study. These samples will be analysed for markers of inflammation.

1. Corona sulcus swab

Samples will be transported to the University of Cape town after 50% recruitment and at the end of the study. These samples will be sequenced, and microbiome species quantified.

1. Rectal swab

Samples will be transported to the University of Liverpool after 50% recruitment and at the end of the study. These samples will have drug levels measured.

1. Urine sample

Mid-stream urine samples (5ml) will be transported to the University of Liverpool after 50% recruitment and at the end of the study. These samples will have drug levels measured.

1. End point Sample Analysis
2. Ex vivo challenge of foreskin tissue and PBMCs

Quantification of protection from HIV-1 infection in foreskin tissue and PBMCs will be initially carried out in local laboratories. In brief, foreskin explants will be challenged or not (for negative background control tests) *ex vivo* with HIV-1BaL at two titers; a high titer normally used in pre­clinical assays in the laboratory and a low titer trying to mimic *in vivo* levels. *Ex vivo* challenge will be performed using a non-polarized system by submerging the tissue explant in 200 pl of virus diluted at the appropriate titer. Following 2 h of incubation, explants will be washed with PBS to remove unbound virus and transferred to a fresh culture plate. Tissue explants will then be cultured for 15 days and maintained by harvesting approximately two-thirds of culture supernatant at days 3, 7, 11 and 15, and refeeding the cultures with fresh medium. The extent of virus replication in tissue explants will be determined by measuring the p24 antigen concentration for HIV-1 in supernatants at each harvest time point with antigen-specific ELISA. The supernatant fluid collected from the culture supernatants will be stored and subsequently analysed for HIV infection at Imperial College London.^16-18^ Interim analyses after 30% and 60% recruitment will confirm continued validation of assays and whether recruitment needs to be extended in any arm.

*Ex vivo* dosing of foreskin explants with FTC-TDF or FTC-TAF will be used to i) mimic the post­coitus dosing in the IPERGAY trial; and ii) evaluate the time frame for initiating PEP with FTC- TDF or FTC-TAF. In the first case, to mimic IPERGAY, foreskin samples obtained from CHAPS participant who will have received one or two pills of FTC-TDF or FTC-TAF, will be dosed by submerging the explant in 200ul of drug diluted at the appropriate concentration, 18hrs after *ex vivo* viral challenge.

To pre-clinically evaluate the potential of FTC-TDF and FTC-TAF as PEP drugs and determine the maximum time after HIV exposure that PEP can be initiated effectively, we will use foreskin specimens from participants in the control arm of CHAPS who will not have taken FTC-TDF nor FTC-TAF prior to circumcision. Foreskin explants will be dosed in a non-polarized manner as described above either 1 h, 24h, 48 h or 72 h post-viral *ex vivo* challenge. During *in vivo* dosing, formulations contain drug levels in excess of the known *in vitro* inhibitory concentration. Hence, for both objectives [i) and ii)], a fully *ex vivo* inhibitory dose of drug (1 mg/ml)^22^ will be used.

1. Drug level measurement

Concentrations of FTC, TFV and TAF, including intracellular triphosphates (TFV-DP, FTC-TP) will be measured in the plasma, urine, rectal secretions and foreskin (tissue homogenate as well as isolated CD4+ cells from the foreskin) of volunteers, as shown in Table 3, at the Liverpool GCP Bioanalytical Facility, UK, UK. PBMCs and CD4+ expressing cells isolated from whole blood will be stored for further determination of intracellular TFV-DP/FTC-TP which is not included in the costs of the study.

1. Microbiome

The University of Cape Town will determine the microbiome of skin the penis, using sterile moistened HydroFlock® swabs. The microbiome will be analysed using 16S rRNA sequencing to identify the diversity, relative and differential abundance of microbes of the coronal sulcus.

1. Cytokines:

An array of cytokines/chemokines will be measured using an in house Luminex multi-bead array designed by Imperial College and use culture supernatants derived from the PBMC and foreskin tissue which have been exposed to HIV using ex vivo challenge.

1. Gene expression

The Karolinska Institute will analyse genes that are involved in inflammation processes in foreskin tissue and which may be potentially affected by treatment. The analyses will be conducted with commercially available microarrays which assemble genes related to inflammation.

1. Immune cells in foreskin tissue

The UCT will analyse cut blocks of tissue using a combination of immunohistochemistry immunoflourescence microscopy for the numbers of activated HIV target cells (Langerhan's cells, CD4+CCR5+ T cells and dendritic cells) and their location in the inner and outer foreskin. The analysis will be performed on a subset of foreskin tissues from trial participants who show either no ex-vivo protection or >75% ex vivo protection.

1. Proteomics

UCT will analyse stored foreskin tissue for targeted tight junction proteins as a measure of

tissue and barrier integrity

Table 3: Sample collection and sample analysis

| **Specimen type** | **Volume/amount of sample** | **analysis** |
| --- | --- | --- |
| Safety blood | 4ml EDTA  5ml SSAT | Haemoglobin  HIV |
| Research blood | 51mls ACD | Ex vivo challenge, drug levels, cytokines |
| Rectal swab | 1 swab | Drug levels |
| Urethral swab | 1 swab | Inflammation |
| Coronal sulcus swab | 1 swab | Microbiome |
| Foreskin | Circumcised foreskin | ex vivo challenge, drug levels, inflammation and microbiome |
| Urine | 5ml  5ml | Drug level  STI screen |

Table 4: Drug levels to be measured at VMMC visit

|  | **Parent drug:**  **TAF/TFV/FTC** | **Metabolite:**  **TFV-DP/FTC-TP** |
| --- | --- | --- |
| Blood: |  |  |
| - Plasma * | X |  |
| - CD4+ expressing cells in PBMC * |  | X |
| Urine* | X |  |
| Rectal fluid (optional)* | X |  |
| Foreskin: |  |  |
| - CD4 expressing cells |  | X |
| - non CD4+ population |  | X |
| - Total tissue homogenate | X | X |

*Blood, urine and rectal samples must be taken 0 to 40 mins prior to VMMC

1. Assessment of Safety
   1. Specification, Timing and Recording of Safety Parameters

Participant safety will be determined by physical examination, blood tests and adverse event

reporting.

- 1. Management of potential adverse outcomes after VMMC

Study participants will be given the locally approved routine information regarding which symptoms should alert them to the possibility of infection or a major bleed following their VMMC. In the event of these symptoms they will be advised to attend their local Hospital and to take with them their VMMC Patient Information sheet and inform the medical staff that they have recently undergone VMMC.

- 1. Management of HIV-1 diagnosis

Volunteers who are found to be HIV-1 infected at screening will be provided with HIV counselling and referred to HIV care according to local guidelines.

- 1. Procedures for Recording and Reporting Adverse Events

**Adverse Events**

An AE is defined as any untoward medical occurrence (including an abnormal laboratory finding) in a clinical research participant which may or may not have a causal relationship with the study product. Study product refers to TDF and FTAF. Recording of AEs for this study beginning from the time of randomisation.

Adverse Event Reporting

Symptoms or conditions that occur prior to randomisation will be recorded in the participant's medical history as pre-existing conditions. All new or worsening symptoms or conditions that occur following randomisation will be considered AEs and will be recorded on the AE CRF regardless of severity and relationship to study product. AEs that are ongoing at the time of study exit will be followed up for up to 30 days after study exit and then, if not resolved, will

be referred to a health care provider for further follow-up. After this period sites must report serious, unexpected, clinical suspected adverse drug reactions if the study site becomes aware of the event on a passive basis, i.e., from publicly available information.

Study staff will document in source documents and the appropriate CRF AEs reported by or observed in enrolled study participants regardless of severity and presumed relationship to study product. Relatedness is an assessment made by a study clinician of whether or not the event is related to the study agent. The relationship of all AEs to study product will be assessed as either related or not related.

AE severity will be graded according to the grading table. (eg. DAIDS grading table) This version will be used for the entire duration of the study. For this study, all Grade 3 or above events or reactions will be reported as adverse events.

Sites may be required to submit AE and SAE information to local regulatory agencies or other local authorities per in country guidelines.

Study participants will be provided with contact telephone numbers to report any AEs they may experience, except for life-threatening events, for which they will be instructed to seek immediate emergency care.

Serious Adverse Event (SAE) Reporting

An SAE includes any experience that is:

- fatal or life-threatening,
- results in persistent or significant disability/incapacity,
- requires or prolongs hospitalization,
- is a congenital anomaly, or is an important medical event that, based upon medical judgment, may jeopardize the participant and may require intervention to prevent one of the previous four outcomes listed above.

A life-threatening AE means that the participant was, in the view of the designated study staff, at immediate risk of death from the condition as it occurred. Notification of deaths will be

recorded by reflecting the medical condition that led to the death on the AE CRF and also reported on the SAE report

Reporting SAEs may require additional reports and follow-up, depending upon the study clinician's assessment of a causal relationship between the study product and the AE(s), and whether the AE(s) is identified in nature, severity, and frequency in the Investigator's Brochure or other risk information supplied to the Investigator.

- 1. Treatment Stopping Rules

The trial may be prematurely discontinued by the Sponsor, Chief Investigator, ethics committee or Regulatory Authority based on new safety information. If the study is prematurely discontinued, active participants will be informed and no further participant data will be collected.

We do not expect any serious adverse events from ARV received during the study- participants receive a maximum of three doses of PrEP only. However, if a participant experiences side effects the drug can be withdrawn at any time. If the drug is stopped prematurely then the participant will be withdrawn from the study and replaced.

1. Statistics

9.1 Sample Size

The total evaluable sample size will be 144 men; 72 in each country. For the primary comparison, data from both countries will be combined, as will the two groups where men receive the same PrEP drug and dose and differ only in timing of PrEP dose prior to circumcision. Comparisons will therefore be between 32 individuals in each treatment group. The study is not powered to detect a difference between these four treatment groups.

9.2 Analysis

Only men who provided a foreskin sample will be included in analysis. Men who withdraw prior to circumcision will be replaced (see section 6.5). Primary groups for comparison will be men who received the same PrEP drug and dose, with no post-exposure drug, as indicated in Figure 1 (Groups A, B, C, D), pooled across the two sites. This means that data from men who received the same PrEP drug and dose, but differed in time between last dose and circumcision, will be

combined into a single group for analysis. If there is a cause to suspect that one of the timings was not effective (i.e. that the timing fell outside the effective window of the treatment regimen) then the groups will also be analysed according to randomisation groups (2-9).

Analyses will be descriptive. The primary analysis will use the result from a p24 antigen assay; p24 antigen level at day 15 after ex vivo HIV exposure; slope of p24 antigen level (day 3 to day 15) and area under the curve (day 3 to day 15).

To evaluate in vitro dosing FTC-TDF and FTC-TAF, analysis will be within randomisation groups - that is, men who received the same oral PrEP dosing. The results from foreskin tissue exposed to in vitro drug will be compared to tissue which was not exposed within an individual. All analysis will be descriptive only; we will report results in each group, and 95% confidence intervals.

The analysis plan for estimating the pharmacodynamic dose-response curves will follow previously described methodology.^24^ The dose-response relationships between percentage protection and time since drug initiation and drug discontinuation following steady state will be generated for each drug. In addition, the relationship between pharmacokinetic parameters (concentrations of drug in rectal fluid, foreskin and plasma), pharmacodynamic outcomes, cellular activation and microbiome will be evaluated.

All demographic (age, height, weight, BMI, ethnic origin, etc.), clinical (physical examination, medical history, concomitant diseases) characteristics and survey responses will be tabulated and analysed descriptively by country.

Quantitative data generated from the socio-behavioural, willingness to participate and exit survey will be analysed using Statistical Analysis Software (SAS), Statistical Package for the Social Sciences (SPSS) or Stata. Preliminary analyses will include checks for missing values, data range, and outliers. Normality will be examined using Q-Q plots and continuous data will be assessed for transformations or categorizations. Bivariate analyses will include Chi-square and Fisher's Exact tests to compare categorical variables, and T-tests or Wilcoxon tests for continuous variables. Descriptive statistics by site will be used to describe the study sample, and assess factors associated sexual behaviour, PrEP acceptability and clinical trial feedback/experience. The precise definition of outcomes will be defined during the formative research.

Qualitative data will be organised and managed using a common method of analyses using software or manual methods, depending on the site. Data analysis from the IDIs will be conducted through the Framework Analysis approach.^25^ Framework analysis is becoming a popular approach in qualitative research within clinical trials.^26^ It was developed in the 1980's by UK social science researchers and has been used extensively for applied or policy relevant qualitative research.^27^ Framework analysis provides a highly systematic method of categorizing and organizing data according to key themes, concepts and emergent categories in grids or matrices. The best aspect of using framework analysis is that it provides an opportunity to link qualitative findings with those of quantitative research. This will be relevant in comparing, contrasting and enhancing findings collected quantitatively, thus increase the validity of findings by using triangulation methods.^28^ Data analyses will be undertaken by trained research team members experienced in qualitative data analyses. The research team will be trained on the process of framework analysis by a qualified social science researcher, to ensure a consistent approach to analysis. Each transcript will be coded by at least two researchers at each research site and assessed for consistency in coding. Any disagreements about the codes will be brought to the research team and discussed until agreement is reached.^29^

1. Criteria for termination of the trial

The Sponsor or Investigator may terminate either part of, or the entire trial for safety or administrative reasons. A written statement fully documenting the reasons for such a termination will be provided to the Ethics Committee and the Regulatory Authorities as appropriate.

1. Trial Management Group (TMG)

A Trial Management Group (TMG) will be formed comprising the Chief Investigator, other lead Investigators (clinical and non-clinical) and Trial Management Team. The TMG will be responsible for the day-to-day running and management of the trial. It will hold monthly meetings throughout the trial.

1. Trial Steering Committee (TSC)

The Trial Steering Committee (TSC) has membership from the Trial Management Group (TMG) plus independent members, including the Chair. The role of the TSC is to provide overall supervision for the study and provide advice through its independent Chair. The ultimate decision for the continuation of the study lies with the TSC.

1. Independent Data Monitoring Committee (IDMC)

An Independent Data Monitoring Committee (IDMC) will be formed. The IDMC will be the only group which sees the confidential, accumulating data for the study separately by randomised group. The IDMC will review study data on recruitment, safety, adherence to randomised strategies and efficacy, as well as consider findings from any other relevant studies. The IDMC will meet after the first 10 participant have completed the study the frequency of meetings will be dictated in the IDMC charter. The IDMC will consider data using the statistical analysis plan and will advise the TSC. The IDMC can recommend premature stopping, reporting or modification.

1. Direct Access to Source Data and Documents

Every effort will be made to protect participant privacy and confidentiality. Study-related information will be stored securely at the study site. All participant information will be stored in lockable file cabinets in areas with access limited to study staff. Data collection, administrative forms, laboratory specimens, and other reports will be identified by a coded number only to maintain participant confidentiality.

Databases will be secured with password-protected access systems. Forms, lists, logbooks, appointment books, and any other listings that link PID numbers to other identifying information will be stored in a separate, locked file in an area with limited access. Clinical information will not be released without written permission of the participant or participant's parent/legal guardian, except as necessary for monitoring by:

- Authorized study representatives
- In country Health Regulatory Authorities
- Relevant Research Ethics Committees
- Other Regulatory Agencies
- Monitors and Auditors

1. Ethics & Regulatory Approvals

This study will be with oversight by the South African Health Products Regulatory Authority (SAHPRA) in accordance with South African standards of Good Clinical Practice (GCP); for sites in other countries, additional oversight will be provided by the relevant national regulatory authorities and local GCP guidelines will also apply.

The study also will be conducted under the oversight of site specific RECs. The study will only be initiated after it has been approved by all relevant ethics committees and will be conducted in accordance with all conditions of approval by the ethics committees.

All participating ethics committees will be properly constituted and provide oversight in accordance with the applicable provisions of the International Conference on Harmonisation Tripartite Guideline for Good Clinical Practice, ICH E6, and South African and other applicable national Good Clinical Practice Guidelines. The study will be registered on the South African National Clinical Trials Register, and any other mandatory national registries.

1. Quality Assurance

Monitoring visits will occur in accordance with a sponsor-approved monitoring plan which will stipulate visit timelines, activities and reporting structures.

The Investigators will allow study monitors to inspect study facilities and documentation (e.g., informed consent forms, clinic and laboratory records, other source documents, CRFs to verify that the study is being conducted in accordance with the approved protocol, regulations and guidelines, and that the rights, safety and well-being of trial participants is protected. The monitor will visit staff at study centres at intervals determined in the monitoring plan, and also communicate telephonically and in writing as necessary.

Activities that will be conducted at visits include, but are not limited to, verifying that:

- Patient informed consent forms were signed before any study specific procedures were conducted
- Source document data have been completely and accurately transcribed
- Reporting of adverse events and serious adverse events is complete and in compliance with the protocol and applicable regulations and guidelines
- Investigational product has been correctly dispensed and accountability checks have been correctly performed
- Site facilities remain adequate
- The investigational team is adhering to the protocol as well as ICH and applicable national GCP guidelines

1. Data Handling

The Chief Investigator will act as custodian for the trial data. Study and data staff will receive training site and study-specific standard operating procedures relating to data collection, data entry, and data cleaning. The following guidelines will be strictly adhered to:

1. All patient data will be anonymised. No dates of birth, names or other identifiers will be collected on CRFs or entered into study databases.
2. All anonymised data will be stored on a password protected computer. All trial data will be stored in in accordance with GCP and applicable local regulatory guidelines and policies.
3. A final locked database will be created by the data management team in conjunction with the trial statistician once all queries, inconsistencies and missing variables have been corrected and or attended to. All hard copy source documents will be retained per local guidelines after the end of the clinical trial.
4. Data Management

*Record Keeping*

Participants will not be identified by name on any CRFs. Participants will be identified by a unique study number provided by the Data Management Center (SDMC) upon randomisation.

*Central Data Management*

Data Management will be centralized within the PHRU DMC. Responsibilities include: coordinating the CRF development process; formatting the finalized CRFs; designing and validating the study database; validating the DataFax system; developing a Data Quality Management Plan; implementing central data quality management (i.e., centralized data quality control and data quality assurance procedures); quality control reporting to the sites; other reporting to the sites (such as participant accrual, CRF generation metrics, QC resolution time); exporting data from DataFax for database locks (for interim and final analyses, safety reporting); providing final databases in specified format to designated investigators and sponsors; training site personnel on the sites' data management responsibilities; and providing other project- related assistance and support to site data management, as necessary.

*Data Storage*

Study records will be kept in participant specific binders at each clinic/site. The binders will be stored by each clinic in a secure and hazard free data storage environment, with double lock. Access will be restricted to study personnel authorized to handle study CRF documents. Database files will be password-protected and access to the files will be limited to authorised study staff members only. All data will be backed up at regular intervals, and backups will be stored in secure areas with limited access.

1. Publication Policy

A whole or part of this trial results will be communicated, orally presented, and/or published in appropriate scientific journals. Full anonymity of participant's details will be maintained throughout. Participants wanting to see the results of the trial can request a copy of the article from the investigators once it has been published.

1. Insurance / Indemnity

Insurance for this trial will be obtained by the Perinatal HIV Research Unit. Additionally, all

doctors are required to have evidence of current individual medical liability insurance.

1. Financial Aspects

Funding to conduct the trial is provided by The European & Developing Countries Clinical Trials Partnership (EDCTP).

References

1. Grant RM, Lama JR, Anderson PL, et al. Preexposure chemoprophylaxis for HIV

prevention in men who have sex with men. N Engl J Med 2010; 363:2587-99.

1. McCormack S, Dunn DT, Desai M et al. Pre-exposure prophylaxis to prevent the acquisition of HIV-1 infection (PROUD): effectiveness results from the pilot phase of a pragmatic open-label randomised trial. Lancet 2016 Jan 2;387(10013):53-60.
2. Baeten JM, Donnell D, Ndase P, et al. Antiretroviral prophylaxis for HIV prevention in heterosexual men and women. N Engl J Med 2012; 367:399-410.
3. Getting to Zero: HIV in Eastern and Southern Africa. UNAIDS, 2013
4. Liegler T, Abdel-Mohsen M, Bentley Let al. HIV-1 Drug Resistance in the iPrEx Preexposure Prophylaxis Trial, *The Journal of Infectious Diseases* 210(8) 8; 1217-1227
5. Sax PE, Zolopa A, Brar I, Elion R, Ortiz R, Post F, et al. Tenofovir alafenamide vs. tenofovir disoproxil fumarate in single tablet regimens for initial HIV-1 therapy: a randomized phase 2 study. JAIDS. 2014;67(1):52-8.
6. Kasonde M, Niska RW, Rose C et al. Bone mineral density changes among HIV-uninfected young adults in a randomised trial of pre-exposure prophylaxis with tenofovir- emtricitabine or placebo in Botswana. PLoS One. 2014;9(3):e90111.
7. Massud I, Mitchell J, Babusis D et al. Chemoprophylaxis With Oral Emtricitabine and Tenofovir Alafenamide Combination Protects Macaques From Rectal Simian/Human Immunodeficiency Virus Infection. J Infect Dis. 2016 Oct 1;214(7):1058-62.
8. Ustianowski A, Arends JE. Tenofovir: What We Have Learnt After 7.5 Million Person-Years of Use. *Infectious Diseases and Therapy*. 2015;4(2):145-157.
9. Molina JM, Capitant C, Spire B, et al. ANRS IPERGAY Study Group. [On-Demand](http://www.ncbi.nlm.nih.gov/pubmed/26624850) [Preexposure Prophylaxis in Men at High Risk for HIV-1 Infection.](http://www.ncbi.nlm.nih.gov/pubmed/26624850) N Engl J Med. 2015 Dec 3;373(23):2237-46
10. World Health Organization Guideline on when to start ART and on PrEP for HIV, 2015. Available at<http://www.who.int/hiv/pub/guidelines/earlyrelease-arv/en/>Accessed 19 August 2017.
11. The TenoRes Study Group. Global epidemiology of drug resistance after failure of WHO recommended first-line regimens for adult HIV-1 infection: a multicentre retrospective cohort study. The Lancet Infectious Disease. 2016;16(5):565-75.
12. Cresswell F, Waters L, Briggs E, Fox J, Harbottle J, Hawkins D, et al. UK guideline for the use of HIV Post-Exposure Prophylaxis Following Sexual Exposure, 2015. International Journal of STD and AIDS. 2016;27(9):713-38.
13. Van Damme L, Corneli A, Ahmed K. Preexposure Prophylaxis for HIV Infection among African Women. N Engl J Med. 2012;367:411-422.
14. [Marrazzo JM,](http://www.ncbi.nlm.nih.gov/pubmed/?term=Marrazzo%20JM%5BAuthor%5D&cauthor=true&cauthor_uid=25651245) [Ramjee G,](http://www.ncbi.nlm.nih.gov/pubmed/?term=Ramjee%20G%5BAuthor%5D&cauthor=true&cauthor_uid=25651245) [Richardson BA](http://www.ncbi.nlm.nih.gov/pubmed/?term=Richardson%20BA%5BAuthor%5D&cauthor=true&cauthor_uid=25651245) et al. Tenofovir-based preexposure prophylaxis for HIV infection among African women. [N Engl J Med.](http://www.ncbi.nlm.nih.gov/pubmed/25651245) 2015 Feb 5;372(6):509-18.
15. Cranage, M., Sharpe, S., Herrera et al. Prevention of SIV rectal transmission and priming of T cell responses in macaques after local pre-exposure application of tenofovir gel. PLoS Med. 5(8), e157; discussion e157 (2008)
16. Herrera, C., Cranage, M., McGowan, I., Anton, P., & Shattock, R. J. Reverse transcriptase inhibitors as potential colorectal microbicides. Antimicrob Agents Chemother. 53(5), 1797-1807 (2009).
17. Herrera, C., Cranage, M., McGowan, I., Anton, P., & Shattock, R. J. Colorectal microbicide design: triple combinations of reverse transcriptase inhibitors are optimal against HIV-1 in tissue explants. AIDS. 25(16), 1971-1979 (2011)
18. Schechter M, do Lago RF, Mendelsohn AB et al. Behavioral impact, acceptability, and HIV incidence among homosexual men with access to postexposure chemoprophylaxis for HIV. J Acquir Immune Defic Syndr 2004;35:519-25.
19. [Cardo DM^1^](http://www.ncbi.nlm.nih.gov/pubmed/?term=Cardo%20DM%5BAuthor%5D&cauthor=true&cauthor_uid=9366579), [Culver DH,](http://www.ncbi.nlm.nih.gov/pubmed/?term=Culver%20DH%5BAuthor%5D&cauthor=true&cauthor_uid=9366579) [Ciesielski CA,](http://www.ncbi.nlm.nih.gov/pubmed/?term=Ciesielski%20CA%5BAuthor%5D&cauthor=true&cauthor_uid=9366579) et al. A case-control study of HIV seroconversion in health care workers after percutaneous exposure. Centers for Disease Control and Prevention Needlestick Surveillance Group. [N Engl J Med.](http://www.ncbi.nlm.nih.gov/pubmed/?term=%22cardo%22+A+Case%E2%80%93Control+Study+of+HIV+Seroconversion+in+Health+Care+Workers+after+Percutaneous+Exposure) 1997 Nov 20;337(21):1485-90.
20. [Tsai CC^1^](http://www.ncbi.nlm.nih.gov/pubmed/?term=Tsai%20CC%5BAuthor%5D&cauthor=true&cauthor_uid=9557716), [Emau P,](http://www.ncbi.nlm.nih.gov/pubmed/?term=Emau%20P%5BAuthor%5D&cauthor=true&cauthor_uid=9557716) [Follis KE,](http://www.ncbi.nlm.nih.gov/pubmed/?term=Follis%20KE%5BAuthor%5D&cauthor=true&cauthor_uid=9557716) [Beck TW](http://www.ncbi.nlm.nih.gov/pubmed/?term=Beck%20TW%5BAuthor%5D&cauthor=true&cauthor_uid=9557716) et al. Effectiveness of post-inoculation (R)-9-(2- phosphonylmethoxypropyl) adenine treatment for prevention of persistent simian immunodeficiency virus SIVmne infection depends critically on timing of initiation and duration of treatment. [J Virol.](http://www.ncbi.nlm.nih.gov/pubmed/?term=%22Tsai+CC%22+simian+1998) 1998 May;72(5):4265-73.
21. Otten RA, Smith DK, Adams DR, et al. Efficacy of postexposure prophylaxis after

intravaginal exposure of pig-tailed macaques to a human-derived retrovirus (human

immunodeficiency virus type 2) J Virol. 2000;74(20):9771-9775

1. Fischetti L, Barry SM, Hope TJ, Shattock RJ. HIV-1 infection of human penile explant tissue

and protection by candidate microbicides. AIDS. 2009;23(3):319-28.

1. O'Quigley J and Zohar S. Experimental designs for phase I and phase I/II dose-finding studies. Br J Cancer. 2006 Mar 13;94(5):609-13
2. Ritchie J, Lewis J. Qualitative research practice: a guide for social science students and researchers. London, United Kingdom: Sage; 2010.
3. Galea JT, Kinsler JJ, Salazar X, Lee S-J, Giron M, Sayles JN, et al. Acceptability of Pre­Exposure Prophylaxis (PrEP) as an HIV prevention strategy: Barriers and facilitators to PrEP uptake among at-risk Peruvian populations. International Journal of STD & AIDS. 2011;22(5):256-62.
4. Pope C, Ziebland S, Mays N. Qualitative research in health care. Analysing qualitative data. BMJ. 2000;320:114-6.
5. Mays N, Pope C. Qualitative research: observational methods in health care settings. BMJ. 1995;311:182-4.
6. Rapley T. Qualitative Data Analysis: Some Pragmatics of Qualitative Data Analysis. In: Silverman DE, editor. Qualitative Research: Issues of Theory, Method and Practice. Los Angeles, Unites States: SAGE; 2011. p. 274-90.
