# Supplementary material for: Dose finding study for on-demand HIV pre-exposure prophylaxis for insertive sex in sub-Saharan Africa: results from the CHAPS open label randomised controlled trial
Source: eBioMedicine. 2023 Jun 14;93:104648. doi: 10.1016/j.ebiom.2023.104648 (PMC10275696; doi:10.1016/j.ebiom.2023.104648)
Supplement: CHAPS Study Team list [file mmc3.docx]

**CHAPS Study Team**

In alphabetical order:

Nadia Ahmed, Berenice Alinde, Amara Alieu, Millicent Atujuna, Esther Awino, Linda-Gail Bekker, Christian Callebaut, Francesca Chiodi, Mike Chirenje, Janan Dietrich, Jeffrey Dorfman, Laura Else, Julie Fox, Clive Gray, Christian Holm Hansen, Carolina Herrera, Stefanie Hornschuh, Ayoub Kakande, Pontiano Kaleebu, Charles Kelly, Saye Khoo, Mamkiri Khunwane, Limaktso Lebina, Joseph Makhura, Nomvuyo Mangxilana, Neil Martinson, Susan Mugaba, Richard Muhumuza, Freddie Mukasa Kibengo, Gertrude Mutonyi, Lucia Mungate, Winnie Nabukeera, Rehema Nagawa, Phiona Nalubega, Patricia Namubiru, Stephen Nash, Denis Ndekezi, Teacler Nematadzira, Lumka Nobula, Kyle O’Hagan, Geoffrey Odoch, Daniel Opoka, Sujan Dilly Penchala, Stefan Petkov, Azure-Dee Pillay, Jim Rooney, Elzette Rousseau, Eugene Ruzagira, Alison Sango, Ntombexolo Seatlholo, Janet Seeley, Thabiso Seiphetlo, Jennifer Serwanga, Robin Shattock, Andrew S Ssemata, Lynda Stranix-Chibanda, Gugulethu Tshabalala, Emily Webb, Helen Weiss

| **First name** | **Family name** |
| --- | --- |
| Nadia | Ahmed |
| Berenice | Alinde |
| Amara | Alieu |
| Millicent | Atujuna |
| Esther | Awino |
| Linda-Gail | Bekker |
| Christian | Callebaut |
| Francesca | Chiodi |
| Mike | Chirenje |
| Janan | Dietrich |
| Jeffrey | Dorfman |
| Laura | Else |
| Julie | Fox |
| Clive | Gray |
| Christian | Holm Hansen |
| Carolina | Herrera |
| Stefanie | Hornschuh |
| Ayoub | Kakande |
| Pontiano | Kaleebu |
| Charles | Kelly |
| Saye | Khoo |
| Mamkiri | Khunwane |
| Limakatso | Lebina |
| Joseph | Makhura |
| Nomvuyo | Mangxilana |
| Neil | Martinson |
| Susan | Mugaba |
| Richard | Muhumuza |
| Freddie Mukasa | Kibengo |
| Gertrude | Mutonyi |
| Lucia | Mungate |
| Winnie | Nabukeera |
| Rehema | Nagawa |
| Phiona | Nalubega |
| Patricia | Namubiru |
| Stephen | Nash |
| Denis | Ndekezi |
| Teacler | Nematadzira |
| Lumka | Nobula |
| Kyle | O’Hagan |
| Geoffrey | Odoch |
| Daniel | Opoka |
| Sujan | Dilly Penchala |
| Stefan | Petkov |
| Azure-Dee | Pillay |
| Jim | Rooney |
| Elzette | Rousseau |
| Eugene | Ruzagira |
| Alison | Sango |
| Ntombexolo | Seatlholo |
| Janet | Seeley |
| Thabiso | Seiphetlo |
| Jennifer | Serwanga |
| Robin | Shattock |
| Andrew | Ssemata |
| Lynda | Stranix-Chibanda |
| Gugulethu | Tshabalala |
| Emily | Webb |
| Helen | Weiss |
